# Meeting Abstracts from the 5th National Big Data Health Science Conference

**DOI:** 10.1186/s12919-024-00292-3

**Published:** 2024-05-16

## I1: Introduction: Proceedings of the 5^th^ National Big Data Health Science Conference

### Bankole Olatosi^1,2^, Miranda Nixon^1,3^, Xiaoming Li^1,3^

#### ^1^Big Data Health Science Center, University of South Carolina, Columbia, SC, USA; ^2^Department of Health Services Policy and Management, Arnold School of Public Health, University of South Carolina, Columbia, SC, USA; ^3^Department of Health Promotion, Education, and Behavior, Arnold School of Public Health, University of South Carolina, Columbia, SC, USA

*BMC Proceedings 2024*, **18(8):**I1

We are excited to present this proceeding from the 5th National Big Data Health Science Conference that took place during February 2-3, 2024, in Columbia, SC. This conference once again marks a pivotal moment in the intersection of technology, public health, healthcare, and scientific innovation.

This year’s theme, “Unlocking the Power of Big Data in Health: Empowering Scientific and Healthcare Communities with Data Analytics,” is appropriate for this time and space, considering the astronomical growth in data and computing technologies we have experienced in the last decade. We want to recap and highlight a few things that have happened since our last conference (February 11-12, 2023, Columbia, SC):

- Words like Artificial Intelligence, ChatGPT, and Machine Learning have become ubiquitous for the US population [1,2],
- Digitization of healthcare Big Data is growing rapidly,
- The healthcare industry continues to generate “petabytes” of data representing about 30% of the world’s data, with significant increases in data volume experienced in areas like,
  - ◦ Genomics, molecular research,
  - ◦ Medical image mining,
  - ◦ Population Health and much more [3,4],
- The healthcare big data market is expected to reach $70 billion by 2025 which would represent an almost 600% increase over 10 years [5],
- Big Data analytics has enhanced Public Health at the research, surveillance and intervention levels with widespread use in clinical trials to predict future risk, conduct active surveillance, understand disease, and better target interventions [6].

The growth of healthcare data is influenced by several factors, including increased computing power, storage size, use of electronic health records (EHRs), the adoption of wearable devices, and the expansion of telemedicine, among other factors.

The University of South Carolina (USC) BDHSC is an interdisciplinary enterprise that conducts cutting-edge research and discovery, offers professional development and academic training, and provides service to the community and industry. The Center is comprised of five content cores including, Artificial Intelligence for Sensing and Diagnosis, Electronic Health Records, Genomics, Geospatial, and Social Media, as well as two functional hubs, Business/Entrepreneurship and Technology that collectively promote the utilization of Big Data Analytics in healthcare research, services improvement, and academic training. Since its establishment in 2019 through the USC Excellence Initiative, the BDHSC has made significant progress in enhancing collaborative Big Data health science research across South Carolina and beyond.

Our signature programs include this national conference and a national student case competition. Since the inaugural national conference in 2020, today’s conference has grown in leaps and bounds,

- The previous four conferences attracted 1,500 attendees with 284 presenters,
- The conference is now supported in part by the National Library of Medicine, NIH (R13LM014347)*,
- This recurring national conference is now a symbol of USC’s leadership and commitment to Big Data health science research.

We are poised to explore groundbreaking developments, foster collaboration, and pave the way for transformative advancements in Big Data health science research.

In the era of Big Data, where information flows abundantly and technology evolves at an unprecedented pace, our ability to harness data for the betterment of healthcare has never been more promising. The theme of this conference reflects our commitment to understanding and utilizing the immense potential that lies within the vast datasets at our disposal, while engaging our communities as partners in this process.

Throughout the two days of the 2024 conference, we had a stellar lineup of over 100 presenters, including keynote speakers, experts, and thought leaders who have shared their insights, experiences, and research findings. We delved into topics ranging from precision medicine and predictive analytics to data privacy, ethical considerations, and the role of artificial intelligence in shaping the future of healthcare.

The 2024 conference was not merely a platform for exchanging ideas but a nexus for collaboration. We were proud to host speakers from around the nation and the world, with notable highlights including:

- Speakers and presenters from 38 universities and organizations from 15 states and 5 other nations (China, Italy, Japan, Nigeria, and South Africa).
- Presenters included leaders and experts from 5 NIH Institutes/Offices, SC Department of Health and Environmental Control (DHEC), SAS statistical software, and other government, academia, industry, and healthcare organizations.
- NIH Leadership and Officers hosted a panel discussion on NIH’s priorities and interests in data science research.
- The Conference also showcased the work by current trainees of three NIH funded Big Data training programs (R25 for junior faculty, R25 for community scholars, and T35 for predoctoral students) at USC.

In line with a focus on our community engagement, the conference hosted its second Professional and Career Development Luncheon for Underrepresented Students across South Carolina, an example of USC’s strong commitment to access and opportunity as we help prepare the pipeline for underrepresented future data scientists in healthcare.

A recent Goldman Sach’s report states that *“AI is expected to grow increasingly pervasive as technology develops, revolutionizing sectors including healthcare, banking, and transportation. As a result, the work market will change and necessitate new positions and skills*” [7].

Here are five predictions of how AI will shake healthcare in 2024 [8].

1. Generative AI will help align providers and patients for improved health outcomes (Armed with AI, healthcare providers will be better equipped to allocate more time and expertise to diagnosis and treatment, ultimately raising the bar for patient care and satisfaction).
2. AI will reduce payer friction across the healthcare ecosystem (AI will enhance member experiences across plan selection, provider choice, healthcare financing. By minimizing friction within the payer ecosystem, AI is set to be a driving force behind the creation of more efficient and patient-focused healthcare systems.
3. AI copilots will help combat healthcare workforce shortage (COVID worsened healthcare workforce shortages). In 2024 and beyond, the health system will be forced to rely upon and use AI copilots. For example, community health workers (CHW) can use AI copilots to deliver home-based care. AI can automate patient reminders, arrange transportation, and/or analyze voice markers to detect potential health issues, such as depression and anxiety. The data collected by AI can alert the medical team to follow-up with patients automatically.
4. Greater demand for AI transparency. To avoid harm, transparency will be a focus for AI. Healthcare organizations will demand, track and understand AI models to ensure accurate decision-making, protect patient data, and maintain full transparency and accountability.
5. Compliance and fiscal pressures will force greater reliance on AI for healthcare Administration. AI will optimize administrative workflows in healthcare in 2024 and beyond.

To prepare for AI in the workforce, everyone including industry, government and academia must work together to,

- Champion continuous learning
- Prioritize AI literacy
- Cultivate creativity and innovation
- Establish psychological safety
- Implement flexible work models
- Promote collaborative synergy
- Tailor employee experiences
- Stay at the forefront of AI trends

As the conference attendees engaged in discussions, attended workshops, and networked with fellow participants, many of them seized the opportunity to form connections that could spark the next wave of groundbreaking collaborations that can help us overcome these challenges. Many of the abstracts in this proceeding reflect our ability and ambition in pushing the boundaries of what is possible and accelerate the pace of discovery and innovation as we work to improve population health in the US and globally.

We want to thank all the organizing committee members, sponsors, volunteers, and all those who have worked tirelessly to make this event a reality. Your dedication and commitment to advancing the field of Big Data health science are truly commendable.

In closing, let us embrace the challenges and opportunities that lie ahead. May this (and future) conference be a source of inspiration, knowledge, and collaboration that propels us toward a future where the fusion of Big Data and health science transforms lives and reshapes the healthcare landscape as we know it. We look forward to seeing more participants and more abstracts at the 6^th^ National Big Data Health Science Conference that will take place during February 13-14, 2025, in Columbia, SC.

*Funding for this conference was made possible (in part) by R13LM014347 from the National Library of Medicine. The views expressed in written conference materials or publications and by speakers and moderators do not necessarily reflect the official policies of the Department of Health and Human Services; nor does mention by trade names, commercial practices, or organizations imply endorsement by the U.S. Government.

**References**

1. Johnson, D., Goodman, R., Patrinely, J., Stone, C., Zimmerman, E., Donald, R., ... & Wheless, L. (2023). Assessing the accuracy and reliability of AI-generated medical responses: an evaluation of the Chat-GPT model. *Research square*.
2. Wu, T., He, S., Liu, J., Sun, S., Liu, K., Han, Q. L., & Tang, Y. (2023). A brief overview of ChatGPT: The history, status quo and potential future development. *IEEE/CAA Journal of Automatica Sinica*, *10*(5), 1122-1136.
3. Atasoy, H., Greenwood, B. N., & McCullough, J. S. (2019). The digitization of patient care: a review of the effects of electronic health records on health care quality and utilization. *Annual review of public health*, *40*, 487-500.
4. Galetsi, P., & Katsaliaki, K. (2020). A review of the literature on big data analytics in healthcare. *Journal of the Operational Research Society*, *71*(10), 1511-1529.
5. Healthcare Analytics Market by Type (Descriptive, Predictive, Cognitive), Application (Financial, RCM, Fraud, Clinical, Operational), Component (Services, Software), Deployment (On-Premise, Cloud), End User (Hospitals, Payer) & Region - Global Forecast to 2027. Healthcare Analytics Market. MarketsandMarkets Research Report, Dec 2022 ID: 5439282
6. Cozzoli, N., Salvatore, F. P., Faccilongo, N., & Milone, M. (2022). How can big data analytics be used for healthcare organization management? Literary framework and future research from a systematic review. *BMC health services research*, *22*(1), 1-14.
7. Jared Cohen and George Lee (14 Dec, 2023). The generative world order: AI, geopolitics, and power- Goldman Sachs Intelligence Available at https://www.goldmansachs.com/intelligence/pages/the-generative-world-order-ai-geopolitics-and-power.html
8. AI to take center stage in healthcare in 2024 Robert Connely, Global Market Leader for Healthcare, Pegasystems available at https://www.healthcaredive.com/spons/ai-to-take-center-stage-in-healthcare-in-2024/700098/

## O1: Unveiling the benefits of case competitions for students: the National Big Data Health Science Case Competition

### Dilek Akgun^1,2^, Audrey Auen^1,3^, Bankole Olatosi^1,3^

#### ^1^Big Data Health Science Center, University of South Carolina, Columbia, SC, USA; ^2^Department of Integrated Information Technology, College of Engineering and Computing, University of South Carolina, Columbia, SC, USA; ^3^Department of Health Services Policy and Management, Arnold School of Public Health, University of South Carolina, Columbia, SC, USA

##### **Correspondence:** Dilek Akgun (Akgun@mailbox.sc.edu)

*BMC Proceedings 2024,* **18(8):**O1

**Introduction:** In the ever-evolving landscape of data-driven decision-making, data science has become a staple across industries. Recognizing the importance of practical experience in this field, data science case competitions can serve as invaluable platforms for undergraduate and graduate students to cultivate their analytical acumen, problem-solving abilities, and teamwork skills to tackle real-world challenges [1] applied to healthcare. Furthermore, case competitions represent a dynamic pedagogical approach [2] that transcends traditional classroom boundaries, providing students with a platform to apply a team science [3,4] approach to real-world problems.

**Exploring the Benefits of Data Science Case Competitions in Healthcare**

The astronomical growth of different kinds and modes of healthcare data requires data scientists to possess the necessary competencies to analyze and extract relevant insights and intelligences from multimodal healthcare data. Proficiency in data science is becoming increasingly important in today’s digital health economy. In this regard, case competitions offer myriad benefits to students. This paper examines the benefits of data science case competitions applied to healthcare for undergraduate and graduate students, elucidating how these experiential learning opportunities empower students to thrive in the healthcare data-driven landscape.

*Enhanced Learning Experience*: Participation in data science case competitions offers students a hands-on learning experience that complements and enriches their academic training. This experiential learning approach fosters a deeper understanding of theoretical concepts as students witness firsthand the impact of data-driven decision-making applied to healthcare.

*Development of Critical Thinking and Analytical Skills:* Case Competitions demand critical thinking and creativity to solve multifaceted problems within constraints, fostering analytical skills and resilience in uncertain environments.

*Practical Application of Classroom Knowledge*: Data science case competitions allow students to apply theoretical concepts learned in the classroom to real-world scenarios. By tackling complex, real-life problems, students develop a deeper understanding of analysis, analytical techniques, statistical methodologies, and problem-solving strategies. This hands-on experience reinforces classroom learning and enhances students’ ability to translate theory into practice.

*Innovation and Creativity*: Participants are challenged to devise novel approaches, explore unconventional methodologies, and push the boundaries of traditional data analysis techniques. This fosters a culture of innovation and a mindset of continuous learning and adaptation—a skill set highly valued in today’s rapidly evolving digital landscape.

*Professional Development*: Participation hones project management, time management, teamwork, and presentation skills, building confidence and a competitive edge for the workforce as they work towards analyzing data, formulating insights, and presenting their findings to judges.

*Networking Opportunities*: Data science case competitions provide a platform for students to network with industry professionals, academics, and fellow participants. Interacting with judges exposes students to valuable mentorship from seasoned professionals in the field.

**Use Case: The 2024 Big Data Health Science Case Competition**

The Big Data Health Science (BDHS) Case Competition [5] has been hosted by the University of South Carolina Big Data Health Science Center (BDHSC) since 2020. It occurred before the BDHSC’s other signature event, the National Annual Big Data Health Science Conference [6]. Undergraduate and graduate students from US colleges and universities are eligible to compete in the national BDHS Student Case Competition. The BDHS Case Competition aims to give enthusiastic teams of graduate and undergraduate students the chance to use their analytical skills to tackle a significant healthcare problem using large datasets. As exemplified by the participant testimonials and feedback during the competition, the BDHS Case Competition is a strong use case for realizing the benefits described above. The case competition has attracted 124 teams, including 369 students, from 41 US universities and 1 Chinese university since it launched five years ago. The overall number of students who participated in the BDHS case competition and the number of competing teams and institutions, are shown in Table 1. Figure 1 shows the percentage distribution of participating students by degree.

**Conclusion:** In summary, data science case competitions applied to healthcare represent a transformative educational experience that empowers undergraduate and graduate students to excel in the data-driven economy. By bridging the gap between theory and practice, fostering collaboration, a team science approach, and preparing students as the future workforce and scientists, these competitions serve as a cornerstone of experiential learning in the application of data science to healthcare. This approach could help reduce workforce shortages in data science applied to healthcare by incentivizing non-healthcare majors to consider healthcare as a career.

**References**

1. Baba, Y., Takase, T., Atarashi, K., Oyama, S., & Kashima, H. (2018). Data Analysis Competition Platform for Educational Purposes: Lessons Learned and Future Challenges. Proceedings of the AAAI Conference on Artificial Intelligence, 32(1). 10.1609/aaai.v32i1.11391
2. Burke, M. G., Carter, J. D., & Hughey, A. W. (2013). The Use of Case Study Competitions to Prepare Students for the World of Work. *Industry and Higher Education*, *27*(3), 157-162. 10.5367/ihe.2013.0156
3. Gamble, E., & Jelley, R. (2014). The case for competition: Learning about evidence-based management through case competition. Academy of Management Learning & Education, 433-445.
4. Hall, K. L., Vogel, A. L., Huang, G. C., Serrano, K. J., Rice, E. L., Tsakraklides, S. P., & Fiore, S. M. (2018). The science of team science: A review of the empirical evidence and research gaps on collaboration in science. American psychologist, 73(4), 532.
5. *Big Data Student Case Competition | USC Big Data Health Science Center*. (n.d.). Retrieved February 26, 2024, from https://bigdata.sc.edu/events/student-case-competition/
6. *National Big Data Health Science Center Conference |*. (n.d.). Retrieved February 26, 2024, from https://www.sc-bdhs-conference.org/

**Table 1 (Abstract O1):** Competing teams in the annual BDHS case competition, 2020 – 2024

Year	The Number of		
	Participating Institutions	Teams	Students
2020	8	19	56
2021	14	22	64
2022	10	16	48
2023	15	37	111
2024	17	30	90

**Fig. 1 (Abstract O1):**
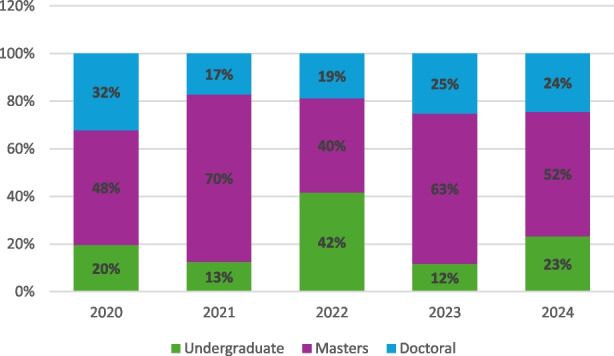
The distribution of participants’ degrees

## O2: Identifying disparities in the relationship between neighborhood walkability and active transportation crashes within South Carolina

### Anna L. Chupak, Shirelle H. Hallum, Farnaz Hesam Shariati, Erin N. Looney, Andrew T. Kaczynski

#### Department of Health Promotion, Education, and Behavior, Arnold School of Public Health, University of South Carolina, Columbia, SC, USA

##### **Correspondence:** Anna L. Chupak (alchupak@email.sc.edu)

*BMC Proceedings 2024,* **18(8):**O2

**Study Objectives:** Pedestrian and bicyclist crashes are of great concern in the Southeast U.S., especially within disadvantaged neighborhoods with poor infrastructure. Little research has evaluated how characteristics of the built environment are associated with active transportation safety, or how this association may differ by neighborhood disadvantage. This study examined the relationship between neighborhood walkability and pedestrian and bicyclist crashes, including variations by social vulnerability across census tracts in South Carolina (SC).

**Methods:** Four key variables were collected for each census tract in SC (*N*=1,103): walkability, pedestrian crashes, bicyclist crashes, and social vulnerability (SV). The EPA’s National Walkability Index (NWI) scores block groups from 1-20 (low to high walkability) and includes key indicators for intersection density, proximity to transit, and land-use diversity; scores were averaged across block groups to determine walkability per tract. Pedestrian (*N*=10,689) and bicyclist (*N*=4,802) crash count and severity were obtained from SCDOT [2011-2021], summed per tract, and adjusted for average trips per day. SV, the degree of susceptibility of a community to hazards, was measured by the CDC SV Index (SVI); a higher percentile rank indicates greater SV. Stepwise linear regressions were conducted to determine the relationship between pedestrian and bicyclist crashes and neighborhood walkability, including interactions between NWI and SVI.

**Results:** Across all SC census tracts, there was a significant, negative relationship between neighborhood walkability and pedestrian crash count and severity per trip (B=-0.005, SE=0.001; B=-1.011, SE=0.192), and for bicyclist crash count and severity per trip (B=-0.019, SE=0.003; B=-2.681, SE=0.489). Significant interactions between NWI and SVI occurred, demonstrating that crashes vary significantly by social vulnerability.

**Discussion:** As neighborhood walkability worsened, pedestrian and bicyclist crash count and severity increased, with even stronger relationships among census tracts with greater SV. These findings help identify neighborhoods in SC in need of infrastructure improvements to address active transportation safety and create equitable environments for all; thereby, facilitating greater physical activity and reducing chronic diseases.

## O3: Black maternal care: a call to action for engineering, data science, electronic health records and justice

### Fay Cobb Payton^1,2^, Michelle Rogers^3^

#### ^1^School of Arts and Sciences, Rutgers University, Newark, NJ, USA; ^2^Department of Business Management, North Carolina State University, Raleigh, NC, USA; ^3^School of Computing and Informatics, Drexel University, Philadelphia, PA, USA

##### **Correspondence:** Fay Cobb Payton (fcpayton@ncsu.edu)

*BMC Proceedings 2024,* **18(8):**O3

**Background:** With the emergence of mHealth and health information technology, engineers and clinicians have come together to capitalize on the possibilities that mobile devices, electronic health records (EHRs) and other information and communications technologies (ICTs) bring to any decision-making context [1]. ICTs have demonstrated beneficial use cases as tools and resources to facilitate communication, creation, dissemination, storage and management of clinical data. Given the disheartening statistics about adverse events in black women’s childbirth experiences, however, the success of ICTs should be brought to bear and not be exclusionary of Black mothers’ knowledge of their bodies, care treatment via midwifery and doulas [2] and more community-based care pre-, during and post-pregnancy.

**Materials and Methods:** There continues to be a dearth of studies of Black women’s preferences regarding labor and delivery and birth outcomes. The pregnancy-related mortality ratios for Black women is 39.9 deaths per 100,000 live births among non-Hispanic Black women and compares to 14.1, 12.8 and 11.6 for non-Hispanic White, Asian and Hispanic mothers, respectively [3]. Recent works published in *American Public Health Association Press* [4] captures the complexities associated with Black women’s reproductive health and examines more holistic approaches, including the use of midwifery and doulas, to provide more equity in birthing experiences.

We will build a corpus of academic literature to identify Black women’s maternal birthing health and use natural language processing methods to uncover themes. We will triangulate these findings via focus groups with clinicians, doulas, midwives and potentially Black mothers to determine the gaps in birthing experience.

**Results:** Our work is in-progress. We will be prepared to report on the results from the data corpus as we triangulate with those noted above. We, however, hypothesize that small data [5, 6] relative to the birthing experience, culturally-centered birthing alternatives, and alternative modes of delivery can critically improve EHRs and health portals [7], if they are to alter the experiences of Black women pre-post-and during the process [8].

**Conclusions:** We offer that the over-reliance on big data fails to capture the expertise and nuisances of Black women’s birthing experiences while heightening biases in care delivery [5]. EHRs should re-innovate to account for the ingenuity of community-based experts, such as doulas, to enable improved medical outcomes and amplify the advocacy of Black maternal care [7]. This work is critical given the need for health policy to address these dire outcomes which are often independent of socio-economic class, education levels and even residential zip codes.

*This abstract was selected as the Best Oral Presentation in the Electronic Health Records Core Breakout Session 1*

**References**

1. Otieno, G. O., et al. Measuring effectiveness of electronic medical records systems: Towards building a composite index for benchmarking hospitals. International Journal of Medical Informatics. 2008; 77:10, 657-669.
2. Baillie, L., Chadwick, S., Mann, R., and Brooke-Read, M., (2013). A survey of student nurses’ and midwives’ experiences of learning to use electronic health record systems in practice, Nurse Education in Practice. 2013; 13:5, 437-441.
3. Division of Reproductive Health, National Center for Chronic Disease Prevention and Health Promotion. https://www.cdc.gov/reproductivehealth/maternal-mortality/pregnancy-mortality-surveillance-system.htm#race-ethnicity, Last updated March 23, 2023.
4. Faustin, Y.F., Black, K. Z. and Hussey. We Are Not a Monolith: Nativity, Racial Discrimination, and Maternal/Infant Health Across the Black Diaspora. In Moss, R. D. (Ed.). Black Women’s Reproductive Health and Sexuality: A Holistic Public Health Approach, 2023. 203-213.
5. Payton, F.C. Without Small Data, AI in Health Care Contributes to Disparities, Scientific American, September 2023.
6. Hicklin, K., Ivy, J., Payton, F.C., Kulkarni, V., Viswanathan, M., and Myers, E. A Bayesian Approach to Value of Information: The Value of Waiting During a Trial of Labor, *Service Science – Special Issue on* Advancing *Health Services*, 2018.
7. Rogers, M. L., & Jeanty, J. Understanding Patient Web Portal Use: An Exploration of Evaluation and Usability Studies. Proceedings of the Human Factors and Ergonomics Society Annual Meeting, 61(1), 603–603, 2017.
8. Gebel C, Hodin S. Expanding Access to Doula Care: State of the Union. 2020. https://www.mhtf.org/2020/01/08/expanding-access-to-doula-care/#:~:text=State%20Doula%20Legislation,%3A%20Indiana%2C%20Oregon%20and%20Minnesota

## O4: Big data and nursing wisdom: the key to unlock healthcare innovation

### Ramya Govindarajan, Judy Katz, Roy Simpson

#### School of Nursing, Emory University, Atlanta, GA, USA

##### **Correspondence:** Ramya Govindarajan (Rgovind@emory.edu)

*BMC Proceedings 2024,* **18(8):**O4

**Issues:** Nurses spend 35%+ of their time entering data into electronic health records (EHRs); yet most do not know how to leverage EHR data to advance nursing knowledge, improve care, or demonstrate nursing’s cost value. While national professional organizations have called for informatics competencies in nursing education for 10+ years (e.g., 2021 *AACN Essentials*), most academic programs have not successfully implemented such curricula. While a comprehensive dataset is an essential tool to teach data science, the cost, complexity, accessibility of such big data sets—as well as faculty time/knowledge—remain prevalent barriers.

**Project:** Project NeLL^TM^ is a suite of applications for teaching and learning nursing data science and its diverse applications in informatics. Designed by nurses for nurses, it systematically addresses the long-standing barriers mentioned above. NeLL contains healthcare data from the Emory healthcare system, one of the largest healthcare systems in Georgia. NeLL includes many component such as a searchable big database for students to explore/download big data, a user friendly front end, an e-learning course introducing data science for nursing students, a proprietary data dictionary, some basic data visualization tools, custom-made curricula for various levels of nursing students, fun animated videos covering a range of topics. The database includes 32T+ deidentified data points representing 1M+ patients across care settings. NeLL resources help faculty to teach data science, even if they are new to the subject themselves. Currently nursing students at Emory University, GA and Rutgers University, NJ use NeLL in their informatics courses to expand their healthcare data knowledge.

**Lessons Learned:** Developing a suite of applications to kick start data science and informatics education in an academic institution requires plenty of time and financial resources. Massive data dictionaries from various divisions of the healthcare system were analyzed in detail to extract relevant fields for educational purposes using focus groups from practicing nurses, nursing students, faculty as well as researchers. Catering to a population with limited exposure to data required innovative ideas to make the query process simple without losing the complexity of real healthcare data. Cutting edge technology was used for safe storage and quick retrieval. All structured data as well as clinical notes were deidentified to avoid complications with IRB clearance to use healthcare data. On the whole, working with various groups of nursing students has been a very rewarding experience. We look forward to many more years of educating nursing students with real healthcare data.

*This abstract was selected as the Best Oral Presentation in the Electronic Health Records Core Breakout Session 2*

## O5: A two-pronged big data approach to critically analyze *Strongyloides stercoralis* infections among rural, impoverished South Carolina residents

### Matthew S. Haldeman^1^, Melissa Nolan^2^, Salomé-Joëlle Gass^3^, Henry Heidt^2^

#### ^1^Department of Family and Preventive Medicine, School of Medicine Columbia, University of South Carolina, Columbia, SC, USA; ^2^Department of Epidemiology and Biostatistics, Arnold School of Public Health, University of South Carolina, Columbia, SC, USA; ^3^Department of Health Service, Policy, and Management, Arnold School of Public Health, University of South Carolina, Columbia, SC, USA

##### **Correspondence:** Matthew S. Haldeman (matthew.haldeman2@prismahealth.org)

*BMC Proceedings 2024,* **18(8):**O5

**Study Objectives:** Soil-transmitted helminths (STHs), a group of human parasitic nematodes, affect up to one third of the global population, persisting primarily among impoverished populations in low-resource settings [1,2]. *Strongyloides stercoralis*, a species of STH, is known to persist throughout the rural, impoverished southeastern United States, including South Carolina, but high-quality prevalence data is lacking due to absence of ongoing surveillance [2–4]. This project aimed to (1) elucidate the estimated current prevalence of human *Strongyloides* infections, and (2) assess clinical epidemiologic risk factors associated with *Strongyloides* infections, among residents of South Carolina.

**Methods:** Two complementary approaches were employed to elucidate the prevalence of human *Strongyloides* infections in South Carolina. First, to estimate prevalence, active surveillance was performed using *Strongyloides* serology testing via strategic sampling of a subset of banked serum samples from the ALL-IN COVID-19 study. Demographic, socioeconomic, and exposure data were collated for those from questionnaires for those ALL-IN study participants. Second, passive surveillance was conducted via electronic health records query at Prisma Health system for *Strongyloides* cases over the time period 8/16/17-8/15/22. Demographic, socioeconomic, risk factor, and health outcomes data were collected for all positive cases and two matched negative controls. For both, participant characteristics were compared between seropositive and seronegative groups, using ANOVA and Fisher’s exact statistics. Lastly, geospatial statistics and were employed to create an infectious disease forecast model for public health intervention.

**Results:** Over the course of the study, we tested a total of 1,458 serum samples for serological evidence of *Strongyloides*, of which 78 (5.35%) were positive. 48 (61.5%) of the positives were female and 30 (38.5%) were male. Age distribution of seropositive participants showed a bimodal distribution, with highest seropositivity in the >64yo age group, followed by the 55-64yo group and the 18-24yo group. Counties with greatest proportions of seropositivity included Chesterfield and Florence, but Spartanburg, Allendale, Hampton, Colleton, Orangeburg, York, and Union counties also had significant positivity. Review of electronic health records at Prisma Health revealed a total of 26 patients diagnosed with *Strongyloides*, of which 20 (76.9%) had significant international travel documented and 7 (26.9%) had refugee status documents; 6 (23.1%) had no travel history documented. For both, statistical analysis of demographic, socioeconomic, and exposure factors, as well as geospatial analysis, are pending and will be completed by January 31, 2024.

**Discussion:** In this study, we found a small but non-negligible prevalence of *Strongyloides* among residents of South Carolina, among both those with international travel and those without, with full analysis still pending. In light of this, *Strongyloides* and perhaps other STHs may be more prevalent in South Carolina than previously expected. Given most clinicians are unaware of this disease—let alone how to test or treat for it—*Strongyloides* could be an unaddressed threat to public health in the state. Further study will be needed to better characterize the burden and geospatial distribution of Strongyloides in South Carolina, which may inform targeted public health interventions in the future.

**References**

1. James SL, Abate D, Abate KH, Abay SM, Abbafati C, Abbasi N, et al. Global, regional, and national incidence, prevalence, and years lived with disability for 354 diseases and injuries for 195 countries and territories, 1990–2017: a systematic analysis for the Global Burden of Disease Study 2017. The Lancet. 2018 Nov;392(10159):1789–858.
2. Montgomery SP, Starr MC. Soil-Transmitted Helminthiasis in the United States: A Systematic Review—1940–2010. Am J Trop Med Hyg. 2011 Oct 1;85(4):680–4.
3. Hotez PJ. Neglected Infections of Poverty in the United States of America. PLoS Negl Trop Dis. 2008 Jun 25;2(6):e256.
4. Lynn MK, Morrissey JA, Conserve DF. Soil-Transmitted Helminths in the USA: a Review of Five Common Parasites and Future Directions for Avenues of Enhanced Epidemiologic Inquiry. Curr Trop Med Rep. 2021 Mar 30;8(1):32–42.

## O6: Optimizing the monitoring of RMNCAH+N program implementation in Northern Nigeria using a data visualization dashboard: the Kaduna state experience

### Collins Imarhiagbe^1^, David Udanwojo^1^, Abimbola Phillips^1^, Ray-Desmond Umechinedu^1^, Terlumun Dyege^1^, Rose Ibanichuka^1^, Omowunmi Obisesan^1^, Obioma Azurunwa^1^, Evelyn Urueye^1^, Francis Ogirima^1^, Pius Christopher-Izere^1^, Bolanle Oyeledun^1^, Adetosoye Adebanjo^2^, Lilian Anomnachi^2^, Olayiwola Jaiyeola^2^, Damilola Olaniyan^2^

#### ^1^Centre for Integrated Health Programs, Abuja, Nigeria; ^2^Technical Advice Connect, Abuja, Nigeria

##### **Correspondence:** Collins Imarhiagbe (cimarhiagbe@cihpng.org)

*BMC Proceedings 2024,* **18(8):**O6

**Issues:** Kaduna State, with 2 million women of reproductive age, implements multiple donor-supported Reproductive Maternal Neonatal Child Adolescent Health Plus Nutrition (RMNCAH+N) interventions across its 23 Local Government Areas (LGA). This multiplicity of interventions posed a challenge for data collection, reporting, and easy visualization of key RMNCAH+N indicators through the official national District Health Information System (DHIS) platform necessary for process monitoring of key performance indicators (KPI) as critical data elements were often not available on the DHIS instance. Non-accessibility for rapid update by the state resulted to the use of multiple excel spreadsheets by health officials and other stakeholders leading to fragmented tabular data presentation, the tedious process of analysis, and minimal data visualization for trend analysis (Figure 1). To mitigate these challenges, Technical Advice Connect, with funding from Bill and Melinda Gates Foundation, supported Centre for Integrated Health Programs to provide technical assistance to the Kaduna State government to create a dashboard to centralize, and visualize data for well informed decision on RMNCAH+N performance as part of interventions to scale up uptake of RMNCAH+N interventions through the Group Antenatal Care model.

**Description of the Project:** Through a consultative process with RMNCAH+N technical working groups, the data needs for a dashboard creation were identified and reviewed in line with KPI. An electronic dashboard was designed to visualize and share routine service data on the state RMNCAH+N at the facility, LGA, and state levels. The development of the electronic dashboard commenced in January 2021 using Tableau Embedded Analytics integrated into a web portal with controlled access at varying user’s levels and was completed over a period of 45 days.

The dashboard identified over 250 indicators, using available data from 2017-2021, at state, LGA, ward, facility levels, and visualizes disaggregation on age, gender, and mode of intervention using an easily navigated filter. The dashboard was linked to DHIS2 via an Application Programming Interface (API) and had data analytics on RMNCAH+N program areas displayed as storyboards on one screen.

The design, development, and testing stages passed through three iterative review meetings involving key government health agencies with demonstration of the dashboard leading to its final publication in April 2022. To enhance sustainability, selected state officials skillful in information technology were trained in administration of the dashboard and to provide users with any needed support.

**Lessons Learned:** Post-RMNCAH+N dashboard implementation in April 2022, at least 250 plus indicators were reported successfully from all components of the RMNCAH+N interventions respectively. Access was provided to 27 state officials who were trained on its use. The dashboard was useful for near real-time review and automated analysis of data for improved program decision making, tracking, and monitoring of RMNCAH+N outcomes such as family planning uptake, hospital delivery by pregnant women, and immunization uptake. This has potential to significantly improve monitoring of the MNCH interventions by state officials and process ownership for overall improvement of maternal outcomes (Figure 2).

**Conclusion:** The RMNCAH+N dashboard was a successful digital intervention which has increased the visibility of and accessibility to data critical for improving decision-making among stakeholders responsible for the health of populations.

**Fig. 1 (Abstract O6):**
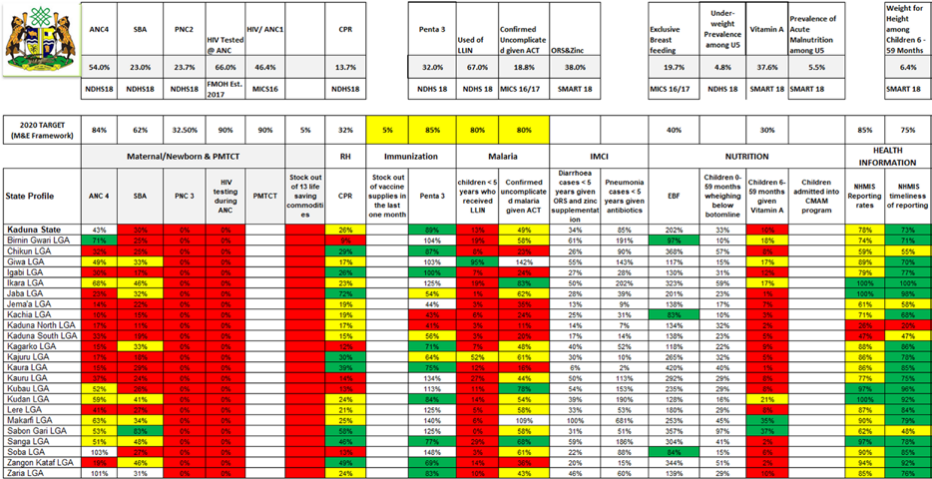
G-ANC monitoring using a manual excel, limiting stakeholders’ effective access to program performance

**Fig. 2 (Abstract O6):**
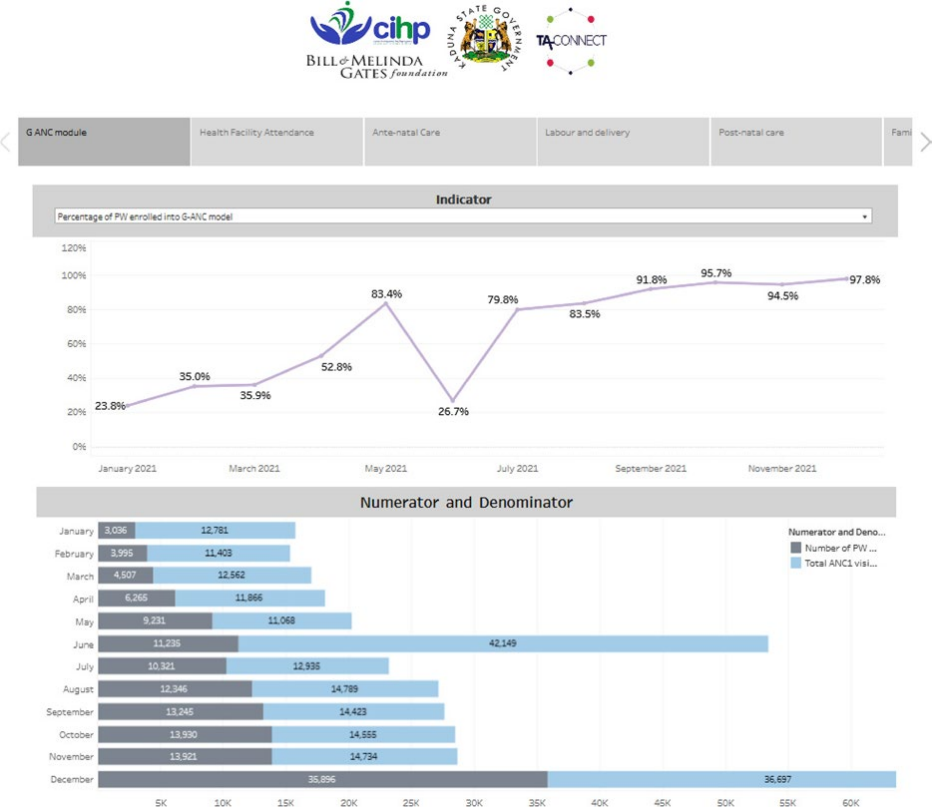
G-ANC monitoring using automated dashboard, aiding real-time access to stakeholders with access control to dashboards

## O7: Disparities in pedestrian and bicycle crashes by social vulnerability across South Carolina

### Andrew T. Kaczynski^1^, Shirelle H. Hallum^1^, Anna L. Chupak^1^, Erin N. Looney^1^, Kelsey M. Thomas^1^, Eleanor Witherspoon^1^, Nathan H. Huynh^2^

#### ^1^Department of Health Promotion, Education, and Behavior, Arnold School of Public Health, University of South Carolina, Columbia, SC, USA; ^2^Department of Civil and Environmental Engineering, College of Engineering, University of Nebraska-Lincoln, Lincoln, NE, USA

##### **Correspondence:** Andrew T. Kaczynski (atkaczyn@mailbox.sc.edu)

*BMC Proceedings 2024,* **18(8):**O7

**Objectives:** Little research about neighborhood disadvantage and pedestrian and bicyclist crashes has controlled for rates of active transportation, examined this relationship in the Southeastern US where active crashes and health disparities are egregious, or employed a comprehensive metric of multiple sociodemographic indicators. This study addresses these gaps by examining inequities in relative rates of pedestrian and bicyclist crashes according to level of social vulnerability (SV) across South Carolina (SC).

**Methods:** SV data and its four dimensions (socioeconomic status, household characteristics, racial/ethnic minority status, housing type and transportation), as measured by the CDC SV index, were compiled for all census tracts (*n*=1,103) within SC. Data for all crashes between 2011-2021 involving a pedestrian (*n*=10,688) and/or bicyclist (*n*=4,802) were obtained from the SC Department of Transportation and geocoded to the respective tract. Total average pedestrian and bicyclist crash severity (Equivalent Property Damage Only) were also calculated for each tract. Both crash frequency and severity scores were adjusted using the annual average number of walking and bicycling trips in the tract based on Streetlight data. Mixed model linear regression analyzed relationships between overall SV and its four dimensions and four crash measures – pedestrian crash frequency, bicyclist crash frequency, pedestrian crash severity, bicyclist crash severity. Stratified analyses were conducted for urban and rural tracts.

**Results:** Overall SV was positively and significantly associated with all four crash outcomes in urban, but not rural, areas: pedestrian crashes per trip (B=0.048,SE=0.012), pedestrian crash severity per trip (B=9.018,SE=2.516), bicyclist crashes per trip (B=0.093,SE=0.029), and bicyclist crash severity per trip (B=16.370,SE=5.482). Similar results were observed for the socioeconomic status and household composition and disability dimensions of SV.

**Discussion:** In urban areas, greater SV is associated with more severe pedestrian and cyclist crash outcomes. Targeted policy, programmatic, and infrastructure interventions are needed to improve active transportation safety and public health.

## O8: A robust rarefaction method for evaluating alpha diversities in TCR sequencing data

### Mo Li^1^, Xing Hua^2^, Michael C. Wu^2^, Ni Zhao^3^

#### ^1^Department of Mathematics, University of Louisiana at Lafayette, Lafayette, LA, USA; ^2^Public Health Sciences Division, Fred Hutchinson Cancer Center, Seattle, WA, USA; ^3^Department of Biostatistics, Johns Hopkins University, Baltimore, MD, USA

##### **Correspondence:** Michael C. Wu (mcwu@fredhutch.org); Ni Zhao (nzhao10@jhu.edu)

*BMC Proceedings 2024,* **18(8):**O8

**Background:** This study focuses on addressing the impact of library size variation on T cell receptor (TCR) diversity analysis, specifically evaluating the commonly used “overall rarefying” method. Library sizes, representing the total number of reads in sequencing experiments, can significantly differ across samples, complicating the accurate estimation and comparison of alpha diversities. While the “overall rarefying” approach is widely applied to address this issue, its effectiveness has not been rigorously assessed.

**Methods:** The research conducted simulations and analyzed real TCR data from 666 healthy bone marrow donors with known cytomegalovirus (CMV) serostatus. Systematic differences were observed in TCR sequence total reads between samples from patients testing positive and negative for CMV serostatus. The real TCR data revealed systematic differences in TCR sequence total reads between samples from patients testing CMV serostatus positive and negative. In response to this issue, we proposed an innovative and robust rarefaction approach to effectively control the confounding effect of library size on alpha diversities.

**Results:** Results from extensive simulations, utilizing both simulated data and real-world CMV data, demonstrated the inadequacy of the “overall rarefying” method in controlling the confounding effect of library size. In contrast, the proposed rarefaction approach exhibited superior performance by achieving better-controlled type-I error rates and enhanced statistical power in association tests. The method outperformed other normalization strategies and significantly reduced the loss of samples and sample sequence reads, particularly for samples with larger sequence total reads.

**Conclusion:** In conclusion, the study highlights the limitations of the “overall rarefying” method in addressing library size variation and introduces a novel rarefaction approach as a more effective solution for TCR diversity analysis. The proposed method demonstrates superior statistical power and provides a valuable tool for researchers in the field.

## O9: Deconstructing multi-institutional EHR data: experiences from a pediatric diabetes surveillance effort in South Carolina

### Angela D. Liese^1^, Rabins Wosti^2^, Jiaying Yi^1^, Caroline A. Rudisill^3^, Jihad S. Obeid^4^, Alex Ewing^5^, Bo Cai^1^

#### ^1^Department of Epidemiology and Biostatistics, Arnold School of Public Health, University of South Carolina, Columbia, SC, USA; ^2^Department of Computer Science and Engineering, College of Engineering and Computing, University of South Carolina, Columbia, SC, USA; ^3^Department of Health Promotion, Education, and Behavior, Arnold School of Public Health, University of South Carolina, Columbia, SC, USA; ^4^Department of Public Health Sciences, Medical University of South Carolina, Charleston, SC, USA; ^5^Prisma Health Data Support Core, Prisma Health, Greenville, SC, USA

##### **Correspondence:** Angela D. Liese (liese@mailbox.sc.edu)

*BMC Proceedings 2024,* **18(8):**O9

**Issues:** The increasing availability of data from electronic health records (EHR) has been touted as the harbinger of a new era of public health surveillance, one that is more cost-effective, timely, and sustainable. Pediatric diabetes mellitus inherently lends itself to EHR-based research because, particularly for type 1 diabetes, the condition is associated with acute complications that require medical treatment soon after onset. After 20 years of conducting traditional active pediatric diabetes surveillance in the state of South Carolina (SC), a new pediatric surveillance effort was initiated in SC, relying exclusively on EHR data.

**Project:** A protocol was developed to determine prevalence of pediatric diabetes using EHR-based surveillance, which governed data extraction, transformation into a national common data model (CDM), transmission, and analyses. Ascertainment of cases of pediatric diabetes mellitus was predicated on the presence of specific ICD-codes in inpatient and outpatient encounters throughout qualifying health care sites statewide regardless of payer. Other data used to define the phenotype included diabetes-relevant diagnostic tests (and associated values, e.g., glucose and HbA1c) and medications. Here, we aim to describe the process-related experience and lessons learned.

**Lessons Learned:** The SC data ascertainment network included providers and institutions previously engaged in the SEARCH for Diabetes Study, including all pediatric endocrinology practices and all major hospital systems in the state. Of the 10 proposed institutions, only one held data coded to the national PCORnet CDM, which resulted in significant efforts to harmonize the ascertained data. The time between request of data to receipt ranged from two to six months and initially received data generally had to be updated. Unfortunately, several institutions declined participation in the EHR-based project, citing lack of data management resources and lack of personnel trained in the protection of human subject in research. Transition of one large institution’s EHR to a different EHR vendor created unforeseen access barriers and ultimately prevented access to legacy data prior to the transition.

Experience to date suggests that relying exclusively on EHR data, i.e., data collected for medical documentation and billing codes, for the surveillance of pediatric diabetes in SC comes with several challenges. The lack of adoption of one CDM by all major health care systems in SC or the availability of a statewide EHR data warehouse that includes all encounter types regardless of payer, is a hurdle for EHR-based research in SC may well result in a shortfall in future nationally funded developments.

## O10: Barriers to unlocking big data to explore LGBTQ+ populations in South Carolina

### Jennifer T. May^1^, Swann Arp Adams^1,2^

#### ^1^Department of Biobehavioral Health and Nursing Science, College of Nursing, University of South Carolina, Columbia, SC, USA; ^2^Department of Epidemiology and Biostatistics, Arnold School of Public Health, University of South Carolina, Columbia, SC, USA

##### **Correspondence:** Jennifer T. May (jm293@mailbox.sc.edu)

*BMC Proceedings 2024,* **18(8):**O10

**Study Objective:** Lesbian, gay, bisexual, transgender, queer, and other sexual and gender minority (LGBTQ+) adults experience greater health disparities compared to non-LGBTQ+ adults (1, 2). There are 20 million LGBTQ+ adults in the United States(3) and of those 1.4 million transgender adults (2). LGBTQ+ adults are more likely to show elevated rates of poor general health, mental distress, and higher likelihood of disability. More than 80% of older LGBTQ+ adults have experienced discrimination or victimization at least once because of their perceived sexual orientation or gender identity (SOGI). Experiences of discrimination and victimization are linked with difficulties in accessing healthcare and poor health outcomes. As a result, an increased interest in improving the clinical care of LGBTQ+ populations have emerged in recent years. A significant barrier in this field is the lack of data identifying these individuals. This is further complicated by the potential to utilize this information to stigmatize and cause harm to this vulnerable group. Consequently, the purpose of this study was to assess the availability of data on LGBTQ+ adults in South Carolina using the South Carolina Integrated Data Warehouse (SCIDW).

**Methods:** We conducted a sequential mixed methods study to examine SOGI data availability. Key informant interviews were used to gauge potential availability and sources of SOGI data in the South Carolina Integrated Data Warehouse. With this information, we conducted descriptive data analysis of 2000 to 2022 data for adults (18 and older) with specific transgender ICD-9 or ICD-10 codes from the sources designated in the interviews.

**Results:** The data available in the SCIDW does not include SOGI data from the state agencies and organizations who are members of the warehouse. The transgender adult population can be explored through specific ICD-9, ICD-10, and DSM-5 codes. Record counts and unique individuals identified were computed from each data source in the SCIDW.

**Discussion:** While it cannot be ruled out that the state agencies and organizations that are members of the SCIDW may be individually collecting SOGI data, we were only able to confirm limited data from specific sources. The lack of access to SOGI data (if it is collected) can lead to biases in findings when studying disparities and resource needs of the LGBTQ+ citizens of South Carolina.

The South Carolina Integrated Data Warehouse is robust and is integral in understanding disparities and resource needs throughout South Carolina. We recommend developing safe, protective methods of providing the option to request SOGI data if it is collected. If state organizations and agencies are not collecting SOGI data, we recommend mandating collection. Without complete SOGI data, policy makers, researchers, community organizations, and others who use the SCIDW will not fully understand the unique needs of LGBTQ+ citizens of South Carolina. There will be a gap in the development of tailored interventions and programs to reduce health disparities among these populations.

## O11: Toward defining a taxonomy for ostomy nursing care using natural language processing

### LaToya McDonald^1^, Melinda Harman^1^, Casey Hopkins^2^, Yuliya Yurko^3^

#### ^1^Department of Bioengineering, Clemson University, Clemson, SC, USA; ^2^School of Nursing, Clemson University, Clemson, SC, USA; ^3^Division of Colon and Rectal Surgery, Prisma Health Upstate, Greenville, SC, USA

##### **Correspondence:** LaToya McDonald (latoyam@g.clemson.edu)

*BMC Proceedings 2024,* **18(8):**O11

**Introduction:** Patient safety and outcomes following ostomy surgery depend on multiple factors, including access to certified ostomy care nurses (COCN) [1,2]. However, there are few COCNs working in community care (<10%) or rural (<2%) settings [5,6]. Nurse-patient interactions (NPI) are documented in electronic health records (EHRs) as nursing care notes, which have a high level of helpful risk factor information and complexity in system and factor extraction in data analysis. This makes EHRs a pivotal resource for evidence-based practice [7,8].

Moreover, data in EHRs are structured into flowsheets (coded data) or free-text inputs (non-coded data), which require different digital tools to extract. Resources such as Knowledge Discovery in Databases (KDD) and the NIH Unified Medical Language System Metathesaurus (UMLSM) are valuable tools for identifying patterns and terminology in various platforms [3,4,9,10]. Machine learning techniques using NIH resources [10] such as the UMLSM through KDD approaches applied to EHRs can provide tools for analyzing NPIs and aid the development of a taxonomy for ostomy nursing care.

**Purpose:** To identify and apply machine learning natural language processing (NLP) methodologies for developing a taxonomy of ostomy nursing care from EHR data.

**Methods:** This IRB-approved retrospective study involved a cohort of *n*=14 patients with CPT codes for ileostomy or colostomy. Data extracted from EHRs included coded data (demographics, medical history, outcomes) and non-coded data (nurse credentials, unstructured nursing care notes within 120 days of ostomy surgery). Trained medical professionals manually extracted text from nursing care notes, identified ostomy-specific patient safety events, and categorized the types of ostomy nursing care provided by registered nurses (RN) and COCNs.

Usage frequency and term frequency-inverse document frequency (TFIDF) of independent words were defined as the number of times a word was included among the separate words in the notes. The UMLS databases were used to identify alternative names and applications. NLP classification was used to identify and categorize data. Tokenization and lemmatization were used to extract key terms from the EHR non-coded data. Word mapping was also used to understand potential semantic and syntactic relationships.

**Results:** In this preliminary analysis, a total of 513 NPIs were analyzed. Approximately 55% of the interactions were from RNs and 45% from COCNs, with variation in the word frequency between RN and COCN notes (Figure 1). Based on word frequency, 12 high-frequency headers and 10 TFIDF headers were identified based on their co-occurrences within text and usage frequencies. (Figure 2)

**Conclusions:** This study supports machine learning EHR analysis to identify different languages nurses use in ostomy care. Based on the preliminary results, NLP applications can be used to identify language differences between RNs and COCNs. Keywords, key terms, and primary words can be placed through NLP applications such as high-frequency and TF-IDF. (Figure 1, Figure 2). The future work for this project aims to define, map, and weigh terms applicable to ostomy nursing care through machine learning methodologies for determining a helpful taxonomy in ostomy care.

*This abstract was selected as the Best Oral Presentation in the AI for Sensing and Diagnosis Core Breakout Session*

**References**

1. Coca C, Fernández de Larrinoa I, Serrano R, García-Llana H. The Impact of Specialty Practice Nursing Care on Health-Related Quality of Life in Persons with Ostomies. J Wound Ostomy Continence Nurs. 2015;42(3):257-263. 10.1097/WON.0000000000000126.
2. Miller LR. Ostomy Care During Hospital Stay for Ostomy Surgery and the United Ostomy Associations of America Patient Bill of Rights: A Cross-sectional Study. J Wound Ostomy Continence Nurs. 2020;47(6):589-593. 10.1097/WON.0000000000000709.
3. Saaty TL. How to Make a Decision: The Analytic Hierarchy Process. J Operational Res Soc. 1973;24(6):19-43. Available from: https://www.jstor.org/stable/25061950.
4. Saaty TL, Katz JM. How to make a decision: The Analytic Hierarchy Process. Eur J Operational Res. 1990;48(1):9-26. 10.1016/0377-2217(90)90057-I.
5. Berti-Hearn L, Elliott B. A Resource Guide to Improve Nursing Care and Transition to Self-Care for Patients With Ostomies. Home Healthc Now. 2018;36(1):43-49.  10.1097/NHH.0000000000000643. PMID: 29298196.
6. Boyle DK, Bergquist-Beringer S, Cramer E. Relationship of Wound, Ostomy, and Continence Certified Nurses and Healthcare-Acquired Conditions in Acute Care Hospitals. J Wound Ostomy Continence Nurs. 2017;44(3):283-292. 10.1097/WON.0000000000000327.
7. Uslu A. JValue of the Electronic Medical Record for Hospital Care. J Med Syst. 2021;23(12):e26323. 10.2196/26323.
8. World Health Organization. Global Strategy on Human Resources for Health: Workforce 2030. 2016. Available from: https://www.who.int/hrh/resources/globstrathrh-2030/en/.
9. Schott LL, Eaves D, Inglese G, Sinha M. Characteristics, Hospital Length of Stay, and Readmissions Among Individuals Undergoing Abdominal Ostomy Surgery: Review of a Large US Healthcare Database. J Wound Ostomy Continence Nurs. 2022;49(6):529-539. 10.1097/WON.0000000000000922.
10. Turner M, Ive J, Velupillai S. Linguistic Uncertainty in Clinical NLP: A Taxonomy, Dataset, and Approach. In: Lecture Notes in Computer Science. LNCS, Volume 12880. Conference paper. First Online: 14 September 2021. DOI: [Add DOI if available].

**Fig. 1 (Abstract O11):**
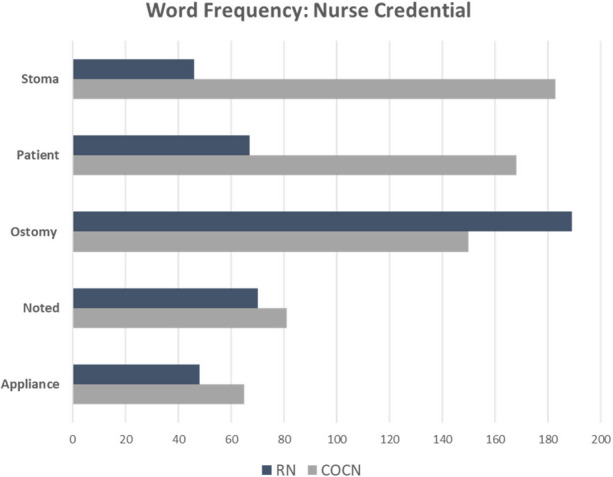
The usage of words identified through tokenization and term frequency indicating how frequently specific terms are employed with RNs’ and COCNs’ clinical text

**Fig. 2 (Abstract O11):**
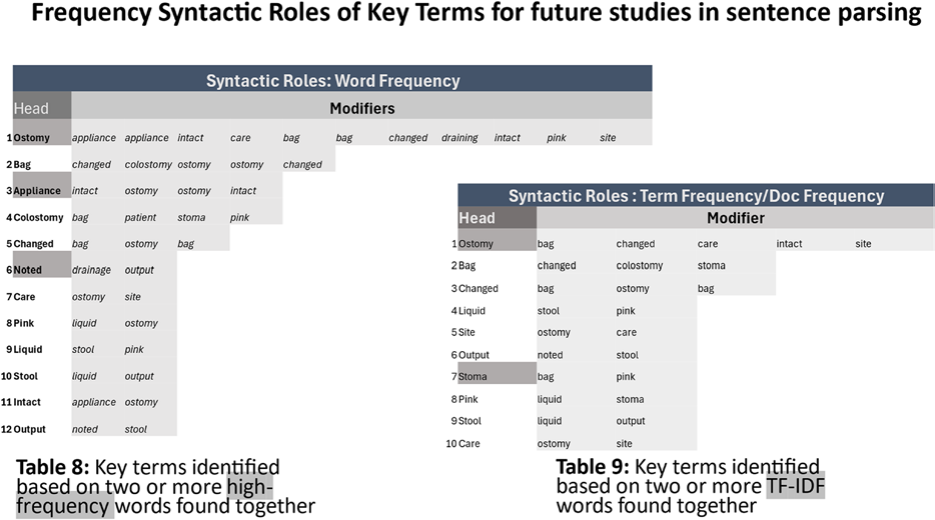
Tables showcasing semantic clusters, identified through grouping high-frequency terms (Table 8) and term frequency-inverse document frequency (Table 9) based on their co-occurrence within the clinical text. The highest frequency words identified between both RN and COCN are highlighted

## O12: Estimating hourly neighborhood population using mobile phone data

### Huan Ning^1^, Zhenlong Li^2,3,4^, Manzhu Yu^1^, Shiyan Zhang^1^

#### ^1^Department of Geography, Pennsylvania State University, State College, PA, USA; ^2^South Carolina SmartState Center for Healthcare Quality, University of South Carolina, Columbia, SC, USA; ^3^Big Data Health Science Center, University of South Carolina, Columbia, SC, USA; ^4^Geoinformation and Big Data Research Laboratory, Department of Geography, College of Arts and Sciences, University of South Carolina, Columbia, SC, USA

##### **Correspondence:** Huan Ning (hmn5304@psu.edu)

*BMC Proceedings 2024,* **18(8):**O12

**Study Objectives**: This research focuses on creating a high-resolution dynamic population map, a fundamental dataset for socio-economic analysis and research, such as hazard exposure estimation [1], and infectious disease transmission [2,3]. In the United States, the Census Bureau provides static population estimates, with the most detailed level being the neighborhood (block group) level. Each year, the LandScan [4] dataset offers gridded population maps that include daytime and nighttime estimates at approximately 90-meter resolution. However, few population maps provide high temporal resolution, like daily or hourly, to facilitate fine-scale and dynamic analyses, such as estimating air pollution exposure in urban areas during weekdays. In this study, we propose a method using smartphone-based human mobility data (Advan Patterns) to reconstruct the hourly population for each neighborhood across the U.S. This is one of the first hourly population maps, contributing to various studies that involve dynamic populations at precise spatiotemporal scales.

**Methods**: We collected the human mobility dataset, Neighborhood Patterns, from Advan (advanresearch.com). This dataset is derived from smart device location data, containing the tracked device counts from home Census block groups (CBG) to other CBGs each month and the hourly device stop counts in each CBG. This study adopted iterative proportional fitting (IPF) to restore the hourly inbound and outbound flows for each CBG. Then, we computed the hourly CBG population according to the Census annual population. The study area is the United States, and the period is June 2023.

**Preliminary Results**: The preliminary results consist of the hourly population for each CBG. Compared with the LandScan daytime grided population, the difference is about 20% at the aggregated county level. For the New York County (Manhattan area), the daytime population from LandScan is 3.40 million, and the hourly population at weekday noon from our results is close (Figure 1, 2.66 million,79.2%), although without considering travelers to the city due to data limitations. The night population is about 2 million, aligning with the previous study [5]. At the CBG level, our result has a difference of about 80% compared to LandScan data.

**Discussion:** The hourly neighborhood population data is formative to public health studies involving rapid environmental changes, such as air pollution exposure and traffic simulation to healthcare facilities. We advocate comprehensive public health studies using dynamic population. It is worth noting the limitations of this study: 1) Multiple assumptions were adopted but are not necessarily true in reality, such as that the visitor’s home CBG distribution in each hour is the same as the monthly distribution. 2) The updated and reliable observation for the dynamic population is limited to comprehensively validate our hourly population estimates.

**References**

1. M. Yu, S. Zhang, K. Zhang, J. Yin, M. Varela, and J. Miao, “Developing high-resolution PM2.5 exposure models by integrating low-cost sensors, automated machine learning, and big human mobility data,” *Frontiers in Environmental Science*, vol. 11, 2023, Accessed: Dec. 04, 2023. [Online]. Available: https://www.frontiersin.org/articles/10.3389/fenvs.2023.1223160S.
2. Chang *et al.*, “Mobility network models of COVID-19 explain inequities and inform reopening,” *Nature*, vol. 589, no. 7840, Art. no. 7840, Jan. 2021, 10.1038/s41586-020-2923-3.
3. H. Ning *et al.*, “Revealing geographic transmission pattern of COVID-19 using neighborhood-level simulation with human mobility data and SEIR model: A case study of South Carolina,” *International Journal of Applied Earth Observation and Geoinformation*, vol. 118, p. 103246, Apr. 2023, 10.1016/j.jag.2023.103246.
4. E. Weber, J. Moehl, S. Weston, A. Rose, C. Brelsford, and T. Hauser, “LandScan USA 2021.” Oak Ridge National Laboratory, Oak Ridge, TN, 2022. 10.48690/1527701.
5. M. L. Moss and C. Qing, “The dynamic population of Manhattan,” *NYU Wagner School of Public Service Working Paper*, 2012, Accessed: Nov. 14, 2023. [Online]. Available: https://www.academia.edu/download/76316859/dynamic_pop_manhattan.pd

**Fig. 1 Abstract O12:**
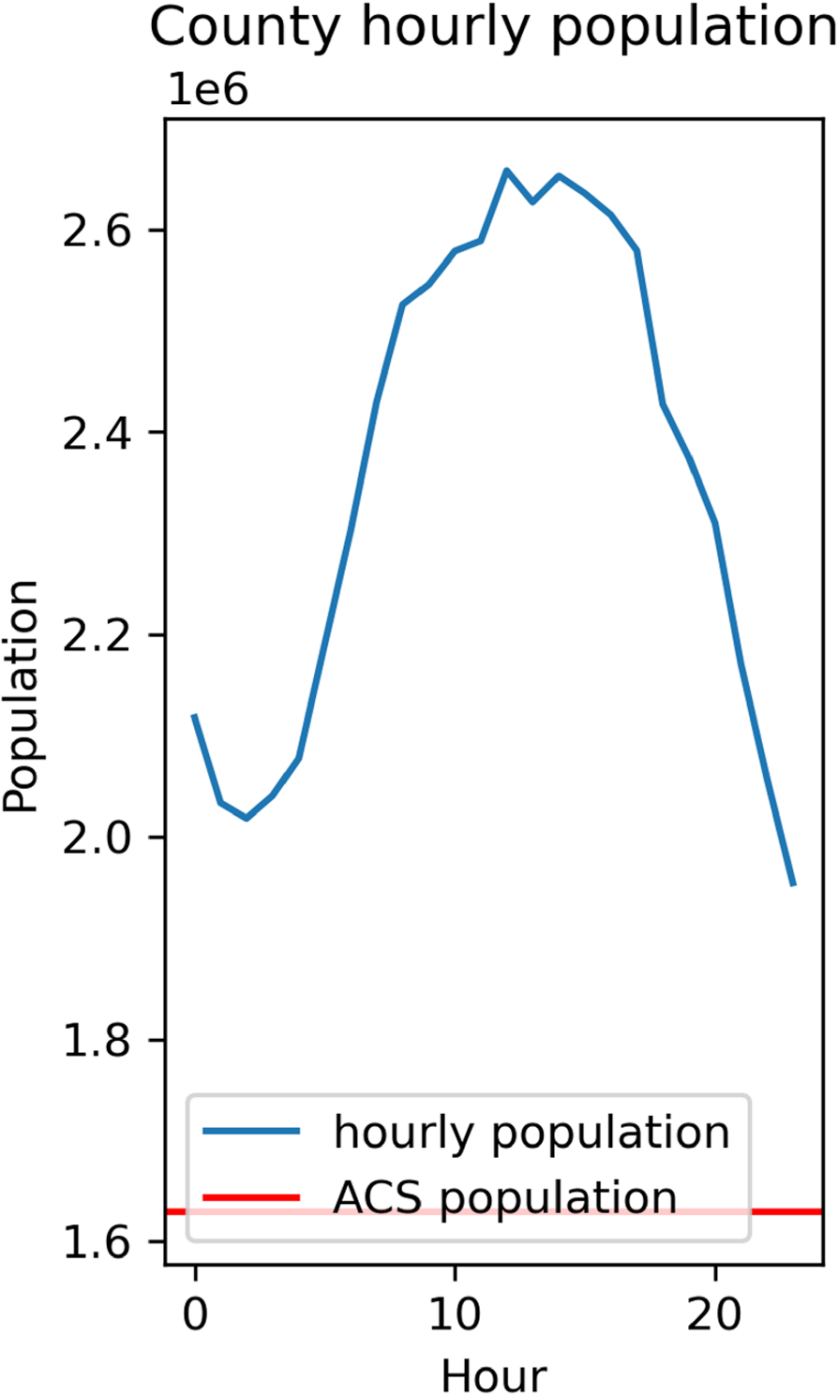
New York County (Manhattan area) hourly population on Monday, June 12, 2023

## O13: Improving healthcare delivery with artificial intelligence: a diagnostic and prescription recommender system

### Damilare Ogungbesan^1^, Misagh Faezipour^2^

#### ^1^Healthcare Informatics, College of Basic and Applied Sciences, Middle Tennessee State University, Murfreesboro, TN, USA; ^2^Department of Engineering Technology, Middle Tennessee State University, Murfreesboro, TN, USA

##### **Correspondence:** Misagh Faezipour (misagh.faezipour@mtsu.edu)

*BMC Proceedings 2024,* **18(8):**O13

**Introduction:** Artificial intelligence (AI) is a transformative force that is reshaping a wide range of industries through improvements in efficiency, decision-making, customer interactions, and overall competitiveness. All facets of contemporary life, including entertainment, business, and healthcare, are being impacted by big data and machine learning [1]. Global aging, rising chronic diseases, and health challenges have elevated the demand for medical care, leading to longer patient waiting times resulting in an urgent need for more healthcare professionals. AI such as clinical decision support systems can improve the uniformity and efficiency of healthcare delivery while lowering medical errors [2]. According to a keyword analysis, AI can help doctors diagnose patients, forecast how diseases will spread, and customize treatment plans [3]. This study aims to show how machine learning and deep learning can create a diagnostic and prescription recommender system to help healthcare professionals deliver more efficient services to patients.

**Objectives**

- Develop an AI-based diagnostic system
- Integrate electronic health record
- Validate and assess accuracy of the system.

**Methodology**

- Obtain representative and varied datasets to test and train the AI system. Incorporate patient demographics, past diagnoses, treatment results, electronic health records, and other pertinent clinical data as can be seen in Table 1.
- Using the prepared datasets, create and train a Random Forest Classifier model, optimizing for precision, sensitivity, and specificity.

**Results:** Approximately 88% of medicine predictive prescription accuracy was achieved with the model. A dataset of 287 rows was used with an 80-20 train-test split. The accuracy is expected to increase with a bigger dataset. The efficiency of healthcare delivery is also expected to improve with the integration of such AI systems into clinical workflows. This could show up as quicker prescription decisions, quicker diagnosis turnaround times, and more efficient use of available healthcare resources.

**Conclusion:** There is great potential for enhancing the delivery of healthcare using AI in the medical field, particularly through the creation and application of a diagnostic and prescription recommender system. The integration of artificial intelligence with healthcare delivery holds significant potential to transform the sector by providing enhanced patient outcomes, and improved diagnostic accuracy.

**References**

1. Secinaro S, Calandra D, Secinaro A, Muthurangu V, Biancone P. The role of artificial intelligence in healthcare: A structured literature review. BMC Med Inform Decis Mak. 2021; 21:1-23.
2. Tadiboina S. N. Benefits of artificial intelligence in healthcare. Webology. 2021; 18(5):3779-3785.
3. Reddy S, Fox J, Purohit M. P. Artificial intelligence-enabled healthcare delivery. J R Soc Med. 2019; 112(1):22-28.

**Table 1 (Abstract O13):** Sample electronic health record for training the AI model

Name	DateOfBirth	Gender	Symptoms	Causes	Disease	Medicine
John Doe	05-15-1980	Male	Fever, Cough	Viral infection	Common Cold	Ibuprofen, Rest
Jane Smith	08/10/1992	Female	Headache, fatigue	Stress	Migraine	Sumatriptan

## O14: Harmonization of multiple HIV related data sources in Sub Saharan Africa: lessons learned from the Boloka Project

### Refilwe Nancy Phaswana-Mafuya^1,2^, Edith Phalane^1,2^

#### ^1^South African Medical Research Council/University of Johannesburg (SAMRC/UJ) - Pan African Centre for Epidemics Research (PACER) Extramural Unit, Johannesburg, South Africa; ^2^Department of Environmental Health, University of Johannesburg, Johannesburg, South Africa

##### **Correspondence:** Refilwe Nancy Phaswana-Mafuya (refilwep@uj.ac.za)

*BMC Proceedings 2024,* **18(8):**O14

**Issues:** Currently, there is no centralized place where key population (KP) HIV surveillance and programming data are gathered and stored in Sub Saharan Africa. Data on KPs are being collected on a smaller scale by numerous stakeholders; there exists an opportunity to harness different data sources. The Boloka data repository, a digital data platform, is being developed as a centralized mechanism for data storage and subsequent analyses to improve our understanding of HIV among KPs as well as monitor program targets. This centralized and standardized database will assist in evaluating progress towards South Africa’s “National Strategic Plan for HIV, sexually transmitted infections, and tuberculosis (2023–2028)” which seeks to reduce new HIV infections, increase ART uptake, and minimize HIV-related deaths by 2030.

**Project:** This paper describes how data storage, management, and harmonization (Stages 3 and 4) for the Boloka Project have unfolded to date (See Figure 1). The data have been placed in a restricted and access-controlled staging area. REDCap is being used for data harmonization to enable provision of much needed timely information at a level and scale that will improve understanding of HIV heterogeneities. Data is pre-processed and converted into a standardized format to create a structured, but flexible and updatable data repository. This includes data cleaning, transformation, and integration to make the data complete for analysis. Various options are being explored for storing data of different types and sizes. To this end we have developed the Boloka Harmonisation Tool, which guides the data harmonization process.

**Lessons Learned:** It is important to predetermine the data storage capacity and versatility at the commencement of the project. There are nuances to be considered in the harmonization of data. Once such hurdles are addressed the potential for high level integrated analysis is formidable. Metadata quality control is needed as well as how to integrate in with the harnessed data. Data harmonization tools need to have essential features such as metadata control, data traceability and security. There is a need for training to address gaps in data storage, management and harmonisation.

**Fig. 1 (Abstract O14):**
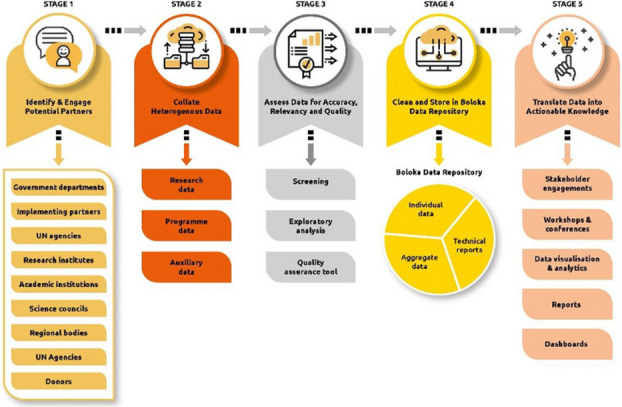
Five stages of the Boloka data repository

## O15: The impact of wildfires on mental health

### Tamara L. Sheldon, Crystal Zhan

#### Department of Economics, Darla Moore School of Business, University of South Carolina, Columbia, SC, USA

##### **Correspondence:** Tamara L. Sheldon (Tamara.Sheldon@moore.sc.edu)

*BMC Proceedings 2024,* **18(8):**O15

**Background:** Climate change is leading to increased drought and elevated temperatures, which are in turn causing more wildfires and wildfire smoke [1,2]. Research on mental health and wildfires is very limited [3], though there is some evidence that chronic exposure to fine particulate matter (PM_2.5_) may be a physiological driver of mental illness [4]. We evaluate the impact of wildfire exposure on mental health.

**Methods:** We combine high-resolution NASA satellite geodata on fire points with wildfire-driven PM_2.5_ concentrations [5] to precisely assess areas of exposure. From NielsenIQ we obtain individual-level data on self-reported ailments, including depression/anxiety, for a nationally representative panel (~50,000 respondents). We link two wildfire data sets to the NielsenIQ data by zip code to obtain an annual panel of households, their socio-demographic characteristics, county of residence, and various measures of wildfire and wildfire smoke exposure from 2011-2017, as well as indicators for whether a household member has experienced depression/anxiety in the prior 6 months. We limit this analysis to California, which is both subject to frequent wildfires and is population dense, having relatively larger sample sizes in the NielsenIQ data. Across the sample 14.5% of households report a householder experiencing depression/anxiety.

**Results:** We estimate two-way fixed effect logit and probit models to predict the probability a household member experiences depression following exposure to a wildfire, controlling for household characteristics and time trends. We test five measures of wildfire exposure: 1) indicator for whether there was a wildfire in a household’s zip code the prior calendar year 2) indicator for whether there was wildfire-related smoke, 3) the number of days wildfire-driven PM_2.5_ exceeded 35 μg/m^3^ the prior calendar year, 4) the number of days it exceeded 50 μg/m^3^, and 5) the number of days it exceeded 100 μg/m^3^.

**Discussion:** While we do not find statistically significant impacts of the first two fire exposure measures, we do find that experiencing more high PM_2.5_ days the prior calendar year significantly increases a household’s probably of experiencing depression/anxiety. Having experienced 5, 10, or 15 days with PM_2.5_ levels above 35μg/m^3^ is associated with probabilities of 17%, 22%, and 28%. Having experienced 5, 10, or 15 days with PM_2.5_ levels above 50μg/m^3^ is associated with probabilities of 19%, 26%, and 34%. In summary, we find exposure to 10 days of elevated PM_2.5_ levels nearly doubles a household’s probability of experiencing depression/anxiety the following year. Our findings can help policymakers understand the human costs of wildfires and can help emergency managers better communicate with communities when preparing for and responding to wildfires.

**References**

1. Mansoor, Sheikh, Iqra Farooq, M. Mubashir Kachroo, Alaa El Din Mahmoud, Manal Fawzy, Simona Mariana Popescu, M. N. Alyemeni, Christian Sonne, Jorg Rinklebe, and Parvaiz Ahmad. “Elevation in wildfire frequencies with respect to the climate change.” *Journal of Environmental Management* 301 (2022): 113769.
2. Liu, Jia Coco, Loretta J. Mickley, Melissa P. Sulprizio, Francesca Dominici, Xu Yue, Keita Ebisu, Georgiana Brooke Anderson, Rafi FA Khan, Mercedes A. Bravo, and Michelle L. Bell. “Particulate air pollution from wildfires in the Western US under climate change.” *Climatic Change* 138 (2016): 655-666.
3. To, P., Eboreime, E. and Agyapong, V.I., 2021. The impact of wildfires on mental health: a scoping review. *Behavioral Sciences*, *11*(9), p.126.
4. Braithwaite, Isobel, Shuo Zhang, James B. Kirkbride, David PJ Osborn, and Joseph F. Hayes. “Air pollution (particulate matter) exposure and associations with depression, anxiety, bipolar, psychosis and suicide risk: a systematic review and meta-analysis.” *Environmental Health Perspectives* 127, no. 12 (2019): 126002.
5. Childs, Marissa L., Jessica Li, Jeffrey Wen, Sam Heft-Neal, Anne Driscoll, Sherrie Wang, Carlos F. Gould, Minghao Qiu, Jennifer Burney, and Marshall Burke. “Daily local-level estimates of ambient wildfire smoke PM2. 5 for the contiguous US.” *Environmental Science & Technology* 56, no. 19 (2022): 13607-13621.

## O16: South Carolina breast and cervical cancer data in action

### Beth Williams^1^, Bezawit Kase^2^, Hallie Heffner^3^, Sonya Younger^3^, Jaron King^4^

#### ^1^Best Chance Network, South Carolina Department of Health and Environmental Control, Columbia, SC, USA; ^2^Cancer Registry, South Carolina Department of Health and Environmental Control, Columbia, SC, USA; ^3^SC Comprehensive Cancer Program, South Carolina Department of Health and Environmental Control, Columbia, SC, USA; ^4^Cancer Programs, South Carolina Department of Health and Environmental Control, Columbia, SC, USA

##### **Correspondence:** Beth Williams (williame@dhec.sc.gov)

*BMC Proceedings 2024,* **18(8):**O16

**Issues:** The South Carolina Central Cancer Registry (SCCCR) is a census of all cancer cases in the state of South Carolina (SC) that collects, processes, analyzes, and publishes cancer incidence data in SC. Operating as one of three arms of the Cancer Prevention and Control program, SCCCR informs all state-led cancer initiatives. The two complimentary arms of the Cancer Prevention and Control program in SC are a breast and cervical cancer screening program – known as the Best Chance Network (BCN) – and the Comprehensive Cancer Control and Prevention (CCCP) program. The issue addressed is how to utilize this vast dataset in public health programming.

**Project:** The South Carolina Central Cancer Registry (SCCCR) is a population-based cancer surveillance system for the state of South Carolina within the South Carolina Department of Health and Environmental Control. The SCCCR collects, processes, analyzes, and publishes cancer incidence and mortality for South Carolina. The data collected by the registry can be utilized to determine the frequency of cancer occurrence in defined areas (county level), to determine the frequency of occurrence in different populations (race, sex, age), and to compare South Carolina cancer statistics (incidence, mortality, and survival) to the United States’s national average. The registry works with internal and external partners to publish information on cancer occurrences and related deaths that can be a referenced by legislators, health professionals, researchers, and the public. The registry submits de-identified data on a yearly basis to the Centers for Disease Control and Prevention (CDC) as well as the North American Association of Central Cancer Registries (NAACCR). The registry has collected cancer data since 1996. Upon request the registry can provide aggregate statistics related to cancer, conduct cancer cluster analysis and provide de-identified raw data for researchers.

**Lessons Learned:** As two separate case studies, the BCN and CCCP program use SCCCR to improve cancer outcomes in SC. Twice a year the BCN program securely sends records of all invasive cervical and invasive and in-situ breast cancer records to the SCCCR for data linkage. The primary objective of the data linkage is to confirm final diagnosis and acquire standardized cancer stage data. Another benefit of this data linkage is a built-in quality assurance measure to ensure missing cancer cases in the SCCCR can be identified. Following this data linkage, geographic and demographic analyses are performed to identify outliers where specific populations are experiencing disparities in cancer outcomes: specifically, disparities in late-stage cancer diagnosis and cancer mortality. Health care providers who serve these populations experiencing disparities are identified and invited to participate in the BCN program to encourage earlier and more regular breast and cervical cancer screening. The BCN program, supported by SCCCR since 1996, has operated continuously in SC since 1991. Recently the CCCP used SCCCR analyses to identify other cancer disparities which led to the inaugural SC Men’s Health Institute in June 2023.

## O17: Heterogeneity of macrophages in staphylococcal enterotoxin B-induced acute respiratory distress syndrome

### Keisha Wilson

#### Department of Pathology, Microbiology, and Immunology, School of Medicine Columbia, University of South Carolina, Columbia, SC, USA

##### **Correspondence:** Keisha Wilson (kiesha.wilson@uscmed.sc.edu)

*BMC Proceedings 2024,* **18(8):**O17

**Background:** Acute Respiratory Distress Syndrome (ARDS) is a severe condition characterized by widespread lung inflammation, often triggered by bacterial toxins such as Staphylococcal Enterotoxin B (SEB). Macrophages play a pivotal role in the pathogenesis of ARDS, exhibiting remarkable heterogeneity in their phenotypic and functional characteristics.

**Methods and Results:** Using single-cell sequencing analysis, we investigated the heterogeneity of macrophages in a SEB-induced ARDS model. We identified five distinct subsets of macrophages, including resident alveolar macrophages and four additional populations designated as MafB, Ifitm6, Ace, and App macrophages. The latter subsets exhibited an M1 phenotype, expressing MHC2 and Ly6C, indicative of pro-inflammatory activation. Notably, MafB and App macrophages also displayed markers associated with an M2 phenotype, such as CD205 and Arg1, suggesting a mixed activation state. Furthermore, all macrophage subsets, except for resident alveolar macrophages, expressed chemokines, implicating their involvement in the recruitment and regulation of immune cells within the inflamed lung microenvironment. Additionally, ATAC data revealed increased accessibility to the promoter regions of interferon-stimulated genes across all macrophage subsets.

**Discussion:** Our findings elucidate the diverse functional states of macrophages during SEB-induced ARDS, highlighting potential targets for therapeutic intervention in this devastating response underlying this condition.

## O18: Utilizing pre-trained language models for identifying vaping-related discussions on Reddit during the EVALI outbreak

### Yang Ren^1^, Dezhi Wu^1^, Erin Kasson^2^, Li-Shiun Chen^2^, Patricia Cavazos-Rehg^2^, Ming Huang^3^

#### ^1^College of Engineering and Computing, University of South Carolina, Columbia, SC 29208; ^2^School of Medicine, Washington University, St. Louis, MO 63110; ^3^McWilliams School of Biomedical Informatics, UT Health, Houston, TX 77030

##### **Correspondence:** Dezhi Wu (dezhiwu@cec.sc.edu); Ming Huang (Ming.Huang@uth.tmc.edu)

*BMC Proceedings 2024,* **18(8):**O18

**Introduction**: The EVALI outbreak refers to the surge of e-cigarette or vaping product use-associated lung injury cases that occurred primarily in 2019 [1]. This outbreak drew significant attention to the potential health risks associated with vaping and e-cigarette use [2]. Online platforms, especially Reddit, serve as rich sources of public perceptions and experiences related to vaping [3]. However, the sheer volume and intricacy of these online posts and comments present a significant challenge for conventional data extraction and analysis methods [4]. This study seeks to address this challenge by employing advanced natural language processing (NLP) techniques. We aim to develop a resilient model capable of efficiently and accurately detecting vaping-related discussions, thereby offering valuable implications for public health monitoring and response strategies.

**Objectives**: The primary objective is to develop a robust NLP method for identifying vaping-related discussions on Reddit, differentiating them from unrelated discourse. This involves capturing the intricacies and context of such discussions, which is often challenging with conventional keyword-based search methods. Secondly, this research endeavors to fill a critical knowledge gap by effectively utilizing pre-trained large language models for the surveillance and analysis of health behavior-related posts on social media.

**Methods**: Our approach began with the creation of a robust gold standard dataset for training and testing an NLP model to identify vaping-related discussions. We identified a list of vaping-related keywords by search literature and then leveraged deep semantic search to expand the keyword list [5]. Sequentially, we extracted posts and comments containing the vaping-related keywords as positive cases from popular vaping subreddits during the EVALI outbreak. We also selected a similar number of posts from non-vaping related subreddits as a negative control group. A clinical team annotated a random sample of 1,000 posts from each of the case and the control groups as the gold standard dataset. After that, we fine-tuned seven different pre-trained language models based on Bidirectional Encoder Representations from Transformers (BERT) with this annotated corpus. These seven BERT models included two generic BERT models (BERT [6] and RoBERTa [7]), three domain-specific models (BioBERT [8], Bio_ClinicalBERT [9], and PubMedBERT[10]), and two source-specific models (BERTweet [11] and RedditBERT [12]). Finally, we combined these fine-tuned language models into an ensemble model to collectively detect vaping-related discussions.

**Results**: The ensemble BERT model showcased impressive performance, achieving an accuracy score of 0.97 and an F1 score of 0.96 in identifying vaping-related content on Reddit. These results underscore the proficiency of the ensemble pre-trained language model in accurately distinguishing vaping-specific discussions within extensive Reddit posts.

**Conclusion:** This study highlights the feasibility and effectiveness of using detection models based on ensemble pre-trained BERT models to identify vaping-related posts on Reddit. Leveraging the integrated power of multiple pre-trained BERT models, this method shows a great potential for screening large volumes of Reddit content. By detecting individuals discussing or networking about vaping, this approach has practical implications in identifying potential vaping users, especially, those at risk of vaping-related health issues. Moreover, it could facilitate potential outreach programs to offer patient support and intervention programs for reducing vaping harms and promoting cessation. This detection model may enable a fast-reacting venue to reach high risk individuals for targeted interventions as epidemics of new substance or health condition arise.

**Acknowledgements:** The authors would like to acknowledge the funding support provided by the University of South Carolina (USC), Columbia, South Carolina, United States (PI: Wu - grant 80002838) and National Institutes of Health (R34 DA054725). The content is solely the responsibility of the authors and does not necessarily represent the official views of the funding agencies.

**References**

1. Winnicka, Lydia, and Mangalore Amith Shenoy. “EVALI and the pulmonary toxicity of electronic cigarettes: a review.” *Journal of general internal medicine* 35 (2020): 2130-2135.
2. Cao, Dazhe James, et al. “Review of health consequences of electronic cigarettes and the outbreak of electronic cigarette, or vaping, product use-associated lung injury.” *Journal of medical toxicology* 16 (2020): 295-310.
3. Wu, Dezhi, et al. “Topics and Sentiment Surrounding Vaping on Twitter and Reddit During the 2019 e-Cigarette and Vaping Use–Associated Lung Injury Outbreak: Comparative Study.” *Journal of medical Internet research* 24.12 (2022): e39460.
4. Sebei, Hiba, Mohamed Ali Hadj Taieb, and Mohamed Ben Aouicha. “Review of social media analytics process and big data pipeline.” *Social Network Analysis and Mining* 8.1 (2018): 30.
5. Guha, Ramanathan, Rob McCool, and Eric Miller. “Semantic search.” *Proceedings of the 12th international conference on World Wide Web*. 2003.
6. Devlin, Jacob, et al. “Bert: Pre-training of deep bidirectional transformers for language understanding.” arXiv preprint arXiv:1810.04805 (2018).
7. Liu, Yinhan, et al. “Roberta: A robustly optimized bert pretraining approach.” arXiv preprint arXiv:1907.11692 (2019).
8. Lee, Jinhyuk, et al. “BioBERT: a pre-trained biomedical language representation model for biomedical text mining.” *Bioinformatics* 36.4 (2020): 1234-1240.
9. Schäfer H, Friedrich CM. Multilingual ICD-10 Code Assignment with Transformer Architectures using MIMIC-III Discharge Summaries. *Conference and Labs of the Evaluation Forum*. 2020.
10. Han, Qing, Shubo Tian, and Jinfeng Zhang. “A PubMedBERT-based classifier with data augmentation strategy for detecting medication mentions in tweets.” arXiv preprint arXiv:2112.02998 (2021).
11. Nguyen, Dat Quoc, Thanh Vu, and Anh Tuan Nguyen. “BERTweet: A pre-trained language model for English Tweets.” arXiv preprint arXiv:2005.10200 (2020).
12. Zhang F. Fan-S/reddit-TC-bert. Hugging Face. 2022 Feb 21. URL: https://huggingface.co/Fan-s/reddit-tc-bert [accessed 2023-12-10]

## O19: What do Foster Parent Associations communicate on Facebook? Analyses using unsupervised machine-learning method

### Anli Xiao^1^, Yanfeng Xu^2^, Linwan Wu^3^

#### ^1^School of Journalism and Mass Communications, University of South Carolina, Columbia, SC, USA; ^2^College of Social Work, University of South Carolina, Columbia, SC, USA; ^3^College of Information and Communications, University of South Carolina, Columbia, SC, USA

##### **Correspondence:** Anli Xiao (axiao@mailbox.sc.edu)

*BMC Proceedings 2024,* **18(8):**O19

**Study Objectives:** About 400,000 children are placed in foster care annually in the US [1]. Foster parents may rely on social media to look for information on raising foster children and seeking peer support [2]. Foster Parent Associations (FPAs) are an important source for foster parents to network with peers, share information, receive training and support, and advocate for themselves [3]. Yet little is known about how FPAs use social media to communicate and interact with them. This study aims to uncover the underlying patterns of FPAs’ communication on social media by answering the question of what FPAs communicate on social media.

**Methods:** A total of 38 state-level FPAs were selected as our study sample as the rest of 13 states’ FPAs’ Facebook pages were unidentifiable [2]. All Facebook posts from these FPAs for the year 2022 were scrapped by using CrowdTangle, and a total of 22,051 Facebook posts from these 38 FPAs’ Facebook pages were collected. To identify topics discussed by these FPAs, we used unsupervised machine learning algorithms of natural language processing in the Python environment. In particular, FPAs’ Facebook posts were analyzed using Latent Dirichlet Allocation, which uncovered hidden topics among a large number of texts.

**Results:** Modeling results indicated that having 3 topics (*k*=3) was the optimal number of topics. After discussing topic similarities and reviewing the literature, we named these three topics from FPAs’ Facebook posts. The first one was the announcement of trainings, conferences, and events, the second one was an expression of appreciation to the community and advocacy for foster families, and the third one was treating foster families as a community that supported each other.

**Discussion:** Our findings suggest that FPAs’ communication with foster parents on Facebook is centered on three topics. The three topics are in partial agreement with previous literature on organizations’ social media communications. Results from this study emphasize the importance of involvement strategy, since expression of appreciation and treating foster families as part of the community are communication tactics that strengthen involvement. Furthermore, this study has uncovered social media communication topic patterns of a highly specialized, yet extremely important area of services. The results of this study yield important theoretical and practical implications for enhancing FPAs’ communication with foster families.

**References**

1. Child Welfare. Key Facts and Statistics - National Foster Care Month - Child Welfare Information Gateway [Internet]. www.childwelfare.gov. 2022. Available from: https://www.childwelfare.gov/fostercaremonth/awareness/facts/
2. Lee JY, Chang OD, Ammari T. Using social media Reddit data to examine foster families’ concerns and needs during COVID-19. Child Abuse & Neglect. 2021 Nov;121:105262.
3. State Foster/Adoptive Family Associations/Coalitions - Child Welfare Information Gateway [Internet]. www.childwelfare.gov. [cited 2023 Dec 4]. Available from: https://www.childwelfare.gov/organizations/?CWIGFunctionsaction=rols:main.dspList&rolType=Custom&RS_ID=32.

## P1: A system dynamics view of patient’s perception of AI and Big Data adoption in healthcare

### Ashiat Adeogun^1^, Misagh Faezipour^2^

#### ^1^College of Basic and Applied Sciences, Healthcare Informatics, Middle Tennessee State University, TN, USA; ^2^Department of Engineering Technology, Middle Tennessee State University, TN, USA

##### **Correspondence:** Misagh Faezipour (misagh.faezipour@mtsu.edu)

*BMC Proceedings 2024,* **18(8):**P1

**Introduction:** The rapid integration of Artificial Intelligence (AI) and Big Data technologies in healthcare is reshaping patient experiences and treatment modalities. As these advancements unfold, understanding how patients perceive the adoption of AI and Big Data becomes important for ensuring patient-centric practices [1]. This extended abstract presents a unique exploration of patient perceptions regarding the adoption of AI and Big Data in healthcare, using a causal loop analysis. The integration of these technologies has profound implications for patient experiences, and this study aims to unravel the causal relationships that shape attitudes, concerns, and potential outcomes.

**Objectives:** The primary goal of this study is to conduct a comprehensive analysis of patient perceptions through the lens of a causal loop. We seek to understand the feedback mechanisms and interconnected variables influencing how patients perceive the integration of AI and Big Data in healthcare.

**Methods:** A causal loop diagram that visualizes the dynamic relationships between key variables is constructed. The diagram incorporates insights from an existing survey [1], emphasizing the benefits of Big Data and the efficiency of AI, along with concerns about data security and privacy. This qualitative analysis complements quantitative findings, providing a comprehensive understanding of the factors affecting patient’s perceptions.

**Results:** The causal loop diagram shown in Figure 1 unveils intricate patterns within patient perceptions of AI and Big Data. Positive experiences, driven by increased trust and higher satisfaction, form reinforcing loops leading to the wider adoption of AI and Big Data. Simultaneously, negative experiences, rooted in decreased trust and lower satisfaction, create balancing loops that may result in resistance to adoption. The incorporation of Big Data benefits and AI efficiency into the causal loop highlights their role as influencers and catalysts within the system. The interplay of these variables emphasizes the delicate balance between positive and negative feedback loops, offering insights into the complex dynamics of patient perceptions.

**Discussion:** This study emphasizes the significance of understanding causal relationships within the patient perception of AI and Big Data. The positive loops underscore the potential for improved patient experiences and increased acceptance of AI-driven solutions. Conversely, the balancing loops underscore the need to address data security and privacy concerns to maintain a patient-centric approach. The causal loop can provide valuable insights for healthcare practitioners, policymakers, and technology developers. Strategies for cultivating positive patient perceptions include education about AI benefits, transparent communication on data security measures, and a continued focus on ethical considerations.

**Conclusion:** This study leverages a causal loop analysis to delve into the complex combination of factors influencing patient perceptions of AI and Big Data adoption in healthcare. By focusing on the causal relationships depicted in the diagram, we gain a deeper understanding of the dynamics at play and unveil actionable insights for fostering a patient-centered approach in the era of evolving healthcare technologies.

**Reference**

1. Yiwei Z., Shiwei S. Exploring patients’ AI adoption intention in the context of healthcare. Springer. 2021. In Digital Health and Medical Analytics: Second International Conference, DHA 2020, Beijing, China, July 25, 2020, Revised Selected Papers 2, pages 27–39.

**Fig. 1 (Abstract P1):**
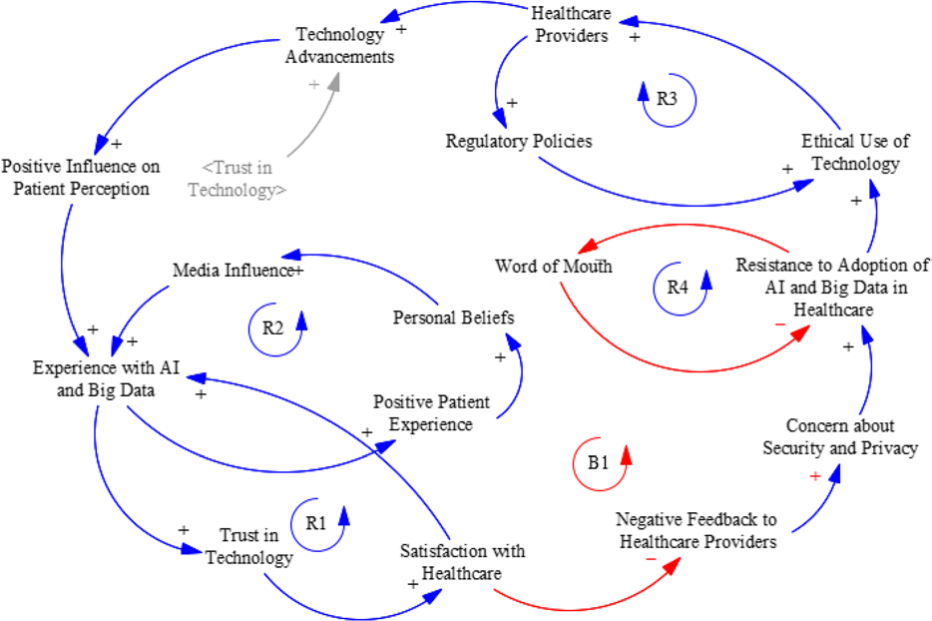
Causal loop diagram on factors influencing patient perception of AI and Big Data in healthcare

## P2: History of pregnancy loss and depression among women of reproductive age: evidence from NHANES 2007-2018

### Syeda Shehirbano Akhtar^1^, Ibitein Okeafor^1^, Fanli Yi^2^, Jihong Liu^2^

#### ^1^Department of Health Services Policy and Management, Arnold School of Public Health, University of South Carolina, Columbia, South Carolina, USA; ^2^ Department of Epidemiology and Biostatistics, Arnold School of Public Health, University of South Carolina, Columbia, South Carolina, USA

##### **Correspondence:** Syeda Shehirbano Akhtar (sakhtar@email.sc.edu)

*BMC Proceedings 2024,* **18(8):**P2

**Background:** Depression is a major public health burden disproportionately affecting the reproductive-aged women [1], with implications on fetal development, birth outcomes, and the overall health of mother and child [2,3]. The hormonal changes during reproductive phase of a woman’s life are associated with increased susceptibility for depression [4-6]. However, the contribution of other psychological stressors such as previous history of pregnancy loss are understudied [7-9].

**Objectives:** This study aimed to examine the association between previous history of pregnancy loss and depression among women of reproductive-age group, and whether the association is modified by the race/ethnicity.

**Methods:** This cross-sectional study utilized the data from the 2007-2018 National Health and Nutrition Examination Survey (NHANES), restricting to women aged 18-49 years old who were pregnant before (*n*=4,288). The presence of depression was defined as the Patient Health Questionnaire (PHQ-9) score ≥10. The previous loss of pregnancy was obtained from the difference between the total number of previous pregnancies and total number of live births and was categorized as 0 (no pregnancy loss), 1 (1 pregnancy loss) and 2 (2 or more pregnancy loss). Multivariable logistic regression models were used to examine association between the history of pregnancy loss and depression after controlling for demographic and maternal morbidity factors. Stratified analysis was performed to determine variations in the association by race/ethnicity.

**Results:** The overall prevalence of depression was 13.36%. The prevalence of depression was higher among women with those with 2 or more pregnancy loss (18.16%), followed by 12.35% among those with 1 pregnancy loss, and 12.27% among those with no pregnancy loss (*p*=0.0009). Women who experienced 2 or more previous pregnancy losses had higher odds for depression compared to those with no pregnancy loss (Adjusted Odds Ratio [AOR]: 1.54 [95% CI: 1.08-1.93]). Multivariable analysis (Table 1) showed significant interaction between maternal race and pregnancy loss (*p*=0.0360). Stratified analysis (Table 2) revealed that the odds of depression was higher among non-Hispanic Blacks who experienced 1 pregnancy loss (AOR: 1.68 [95% CI: 1.12-2.53] and non-Hispanic Whites with 2 or more pregnancy losses (AOR: 1.56 [95% CI: 1.02-2.38].

**Conclusion:** The prevalence of depression among reproductive-aged women increases with higher number of pregnancy loss. Interventions aimed at promoting mental health among reproductive-aged women with previous history of pregnancy loss are advocated especially among Non Hispanic Blacks and NH Whites. Recognizing and addressing the mental health needs of these specific ethnic groups is crucial for developing comprehensive and culturally sensitive strategies that effectively support women who have faced the emotional challenges associated with pregnancy loss.

**Keywords:** depression; pregnancy loss; mental health; maternal race

**References**

1. Accortt EE, Cheadle AC, Dunkel Schetter C. Prenatal depression and adverse birth outcomes: an updated systematic review. Maternal and child health journal. 2015 Jun;19:1306-37.
2. Smith MV, Shao L, Howell H, Lin H, Yonkers KA. Perinatal depression and birth outcomes in a Healthy Start project. Maternal and child health journal. 2011 Apr;15:401-9.
3. Smith KF, Huber LR, Issel LM, Warren-Findlow J. The association between maternal depression during pregnancy and adverse birth outcomes: a retrospective cohort study of PRAMS participants. Journal of community health. 2015 Oct;40:984-92.
4. Soares CN, Zitek B. Reproductive hormone sensitivity and risk for depression across the female life cycle: a continuum of vulnerability?. Journal of Psychiatry and Neuroscience. 2008 Jul 1;33(4):331-43.
5. Broen AN, Moum T, Bødtker AS, Ekeberg Ø. The course of mental health after miscarriage and induced abortion: a longitudinal, five-year follow-up study. BMC medicine. 2005 Dec;3:1-4.
6. Rossen LM, Ahrens KA, Branum AM. Trends in risk of pregnancy loss among US women, 1990–2011. Paediatric and perinatal epidemiology. 2018 Jan;32(1):19-29.
7. Stuart-Parrigon K, Stuart S. Perinatal depression: an update and overview. Current psychiatry reports. 2014 Sep;16:1-9.
8. Dagher RK, Bruckheim HE, Colpe LJ, Edwards E, White DB. Perinatal depression: Challenges and opportunities. Journal of Women’s Health. 2021 Feb 1;30(2):154-9.
9. Faleschini S, Aubuchon O, Champeau L, Matte-Gagné C. History of perinatal loss: A study of psychological outcomes in mothers and fathers after subsequent healthy birth. Journal of Affective Disorders. 2021 Feb 1;280:338-44.

**Table 1 (Abstract P2):** Multivariable logistic regression model with depression among reproductive aged women (18-49 years) ever reported pregnant, NHANES 2007-2018

Independent variables	Depression	
	Crude Odds Ratio (95% CI)	Adjusted Odds Ratio (95% CI)
**Previous history of pregnancy loss**		
No pregnancy loss	1	1
1 pregnancy loss	1.01 (0.79 – 1.28)	0.80 (0.43 – 1.16)
2 or more pregnancy losses	1.59 (1.20 – 2.10)^*^	1.54 (1.08 – 1.93)^*^
**Age category**		
18 – 24 years	1	1
25 – 29 years	0.50 (0.33 – 0.78)^*^	0.60 (0.39 – 0.91)^*^
30 – 34 years	0.74 (0.51 – 1.06)	1.02 (0.70 – 1.49)
35 – 39 years	0.72 (0.51 – 1.04)	0.96 (0.65 – 1.43)
40 – 44 years	0.63 (0.44 – 0.92)^*^	0.87 (0.58 – 1.35)
45 – 49 years	0.74 (0.51 – 1.06)	1.07 (0.71 – 1.60)
**Educational attainment**		
Less than high school	2.14 (1.64 – 2.79)^*^	1.19 (0.87 – 1.63)
High school graduate	1.58 (1.23 – 2.05)^*^	0.99 (0.74 – 1.31)
College graduate	1	1
Race-ethnicity^**^		
Non Hispanic White	1	1
Non Hispanic Black	1.23 (0.99 – 1.52)	0.92 (0.72 – 1.18)
Hispanic	0.95 (0.74 – 1.23)	0.91 (0.72 – 1.16)
Mixed/Other Non Hispanic	0.90 (0.65 – 1.26)	1.22 (0.91 – 1.64)
**Marital status**		
Married	1	1
Other	3.08 (2.45 – 3.87)^*^	1.97 (1.47 – 2. 63)^*^
Unspecified	2.52 (1.90 – 3.34)^*^	1.43 (1.02 – 2.03)^*^
**Family PIR**		
0 – 1.3	4.10 (2.52 – 6.67)^*^	1.97 (1.13 – 3.42)^*^
1.31 – 1.85	2.84 (1.57 – 5.16)^*^	1.80 (0.93 – 3.48)
1.86 – 2.50	1.85 (1.04 – 3.30)^*^	1.20 (0.62 – 2.32)
2.51 – 4.99	1.17 (0.69 – 1.99)	0.84 (0.49 – 1.45)
>5.00	1	1
Undisclosed	2.41 (1.39 – 4.18)^*^	1.67 (0.91 – 3.07)
**Health insurance**		
Insured	1	1
Uninsured	1.44 (1.16 – 1.79)^*^	1.03 (0.80 – 1.33)
**Body Mass Index**		
Underweight	1.79 (0.97 – 3.29)	0.96 (0.48 – 1.91)
Normal weight	1	1
Overweight	1.29 (0.93 – 1.79)	1.17 (0.84 – 1.63)
Obese	1.88 (1.44 – 2.45)^*^	1.43 (1.09 – 1.88)^*^
**Smoking**		
Never	1	1
Former	1.83 (1.30 – 2.58)^*^	1.61 (1.12 – 2.33)^*^
Current	4.67 (3.63 – 5.60)^*^	3.19 (2.42 – 4.19)^*^
**Alcohol**		
Low-risk drinker	1	1
High-risk drinker	1.93 (1.46 – 2.54)^*^	1.10 (0.82 – 1.47)
**Illicit drug use**		
Yes	1.97 (1.60 – 2.43)^*^	1.25 (0.98 – 1.60)
No	1	1
**Gestational diabetes**		
Yes	1.04 (0.76 – 1.44)	0.94 (0.65 – 1.35)
No	1	1
**Diabetes Mellitus**		
Yes	2.25 (1.48 – 3.41)	1.75 (1.06 – 2.88)
No	1	1
**Hypertension**		
Yes	2.09 (1.63 – 2.68)	1.58 (1.17 – 2.13)
No	1	1

*NH* Non-Hispanic

^*^Statistically significant *p* < 0.05 CI -confidence intervals

^**^Interaction of race*pregnancy loss added to model showed interaction effect *p*-value=0.0360

^***^Some of the weighted % do not add up to 100% due to approximation

**Table 2 (Abstract P2):** Stratified analysis of depression and previous history of pregnancy loss by racial groups among reproductive aged women (18-49 years), NHANES 2007-2018

Previous history of pregnancy loss	Depression (Outcome variable)^**a**^					
	Non-Hispanic Black		Non-Hispanic Whites		Hispanics	
	Crude OR (95%CI)	Adjusted OR (95%CI)	Crude OR (95%CI)	Adjusted OR (95%CI)	Crude OR (95%CI)	Adjusted OR (95%CI)
No pregnancy loss	1	1	1	1	1	1
1 pregnancy loss	1.82 (1.26-2.61)^*^	1.68 (1.12-2.53)^*^	0.84 (0.58-1.20)	0.82 (0.56-1.20)	1.00 (0.65-1.55)	0.97 (0.63-1.52)
2 or more pregnancy losses	1.57 (0.90-2.72)	1.07 (0.62-1.84)	1.77 (1.15-2.73)^*^	1.56 (1.02-2.38)^*^	1.48 (0.95-2.31)	1.16 (0.71-1.90)

^*^Statistically significant *p*<0.05

^a^Model used to generate adjusted OR controlled for all the covariates in the study; Mixed/Other race not included due to low sample size

## P3: Rural-urban differences in the prevalence of low back pain and pelvic pain during pregnancy

### Songyuan Deng, Kevin Bennett

#### SC Center for Rural and Primary Healthcare, School of Medicine Columbia, University of South Carolina, Columbia, SC, USA

##### **Correspondence:** Songyuan Deng (songyuan@email.sc.edu)

*BMC Proceedings 2024,* **18(8):**P3

**Objectives:** Musculoskeletal (MSK) changes occur during pregnancy, leading to the development of low back pain (PLBP) and pelvic pain (PPP). Both conditions are associated with prescription opioid use among Medicaid-enrolled beneficiaries. Given the rural-urban disparities in access to rheumatologists and obstetricians / gynecologists, prescription opioid use may also differ. This study aims to describe and compare the rural-urban differences in the prevalence of PLBP and PPP for Medicaid enrolled pregnant women in South Carolina.

**Methods:** This study utilized de-identified 2015-2021 Medicaid claims data provided by the South Carolina Revenue and Fiscal Affairs (SCRFA) Office. This study confirmed 152,309 live births during the period of 2016-2021. Five measures were used to assess pregnancy -related musculoskeletal risks (P-MSKR), including obesity, diagnosed excessive weight gain (EWG), diagnosed excessive fetal growth (EFG), diagnosed diastasis of rectus abdominis (DRA), and diagnosed ligamentous laxity (LL). This study applied the International Classification of Diseases v10 codes to identify these risks as well as PLBP and PPP. Rurality was defined using 2010 rural-urban commuting area (RUCA) codes (1.0-3.0, 4.1, 5.1, 7.1, 8.1, and 10.1 as urban, and the rest as rural), at the ZIP Code Tabulation Areas level. During pregnancy, residence was classified into three levels: always rural, sometimes rural and never. Analysis of the prevalence of PLBP and PPP subset between always/sometimes rural and never rural pregnancies.

**Results:** Among the included pregnancies, 30,692 (20.2%) were always rural, 1,041 (0.7%) were sometimes rural, and 120,576 (79.2%) were never rural. The average prevalence of P-MSKR was 65.6% for all included pregnancies, and that of always rural pregnancies were slightly higher than that of never rural (66.5% vs. 65.4%, *p* < .001). The average prevalence of PLBP was 15.3% for all included pregnancies; that of always rural pregnancies were higher than that of never rural (16.7% vs. 15.2%, *p* < .001), and that of sometimes rural pregnancies were higher than that of never rural (17.9% vs. 15.2%, *p* = .018). The average prevalence of PPP was 25.2% for all included pregnancies; that of always rural pregnancies were higher than that of never rural (27.9% vs. 24.5%, *p* < .001), and that of sometimes rural pregnancies were higher than that of never rural (28.3% vs. 24.5%, *p* = .004). The average prevalence of PLBP / PPP was 33.3% for all included pregnancies; that of always rural pregnancies were higher than that of never rural (36.3% vs. 32.5%, *p* < .001), and that of sometimes rural pregnancies were not significantly different from that of never rural (35.4% vs. 32.5%, *p* = .0503).

**Discussion:** This study found a rural disparity in the prevalence of PLBP and PPP during pregnancy among South Carolina Medicaid enrolled beneficiaries. Future studies may investigate the rural disparity in the risk of prescription opioid use, considering the prevalence of PLBP/PPP. Policymakers can address the gap in the supply and demand for rheumatologists and obstetricians / gynecologists, particularly for rural residents.

## P4: Bayesian semiparametric geoadditive modeling of underweight children under the age of five in Ethiopia

### Endeshaw Assefa Derso^1,2^, Maria Gabriella Campolo^1^, Angela Alibrandi^1^

#### ^1^Department of Economics, University of Messina, Messina, Italy; ^2^Department of Statistics, University of Gondar, Gondar, Ethiopia

##### **Correspondence:** Endeshaw Assefa Derso (enduass@gmail.com)

*BMC Proceedings 2024,* **18(8):**P4

**Objectives:** Early childhood malnutrition can have long-term and irreversible effects on a child’s health and development. This study uses the Bayesian method with spatial variation to investigate the flexible trends of metrical covariates and identify communities at high risk of injury.

**Methods:** Cross-sectional data on underweight were collected from the 2016 Ethiopian Demographic and Health Survey (EDHS). The Bayesian geoadditive model is performed. Appropriate prior distributions were provided for scall parameters in the models, and the inference is entirely Bayesian, using Monte Carlo Markov Chain (MCMC) simulation.

**Results:** The results show that metrical covariates like child age, maternal body mass index (BMI), and maternal age affect a child’s underweight non-linearly. Lower and higher maternal BMIs seem to have a significant impact on the children underweight. There was also significant spatial heterogeneity, and based on IDW interpolation of predictive values, the western, central, and eastern parts of the country are hotspot areas.

**Conclusion:** Our analysis supports the flexible modelling of a child’s age, a mother’s BMI, and a mother’s age. In addition to fixed effects and covariates, there is also considerable evidence of a residual influence on underweight.

**Keywords**: Spatial distribution; Malnutrition; Semi-parametric Bayesian analysis; P- splines; BayesX; MCMC: Ethiopia

## P5: Hypersensitivity associations with antibiotics: a pharmacovigilance study of the FDA Adverse Event Reporting System (FAERS)

### Lucy Edwards, Julia Geith, Chengwen Teng

#### College of Pharmacy, University of South Carolina, Columbia, SC, USA

##### **Correspondence:** Lucy Edwards (lucye@email.sc.edu)

*BMC Proceedings 2024,* **18(8):**P5

**Objectives:** Antibiotics are used to treat or prevent many types of bacterial infections. These drugs work by killing bacteria or preventing them from reproducing and spreading. Antibiotic use can result in a variety of adverse drug events which may range from mild to severe reactions. These events impact drug selection and encourage a patient-specific approach in prescribing to minimize harm. Of the adverse drug events associated with antibiotics, hypersensitivity reactions are very concerning. Drug hypersensitivity reactions occur when the body produces inappropriate immunologic or inflammatory responses to a medication, resulting in symptoms such as rash, anaphylaxis, or serum sickness. Patients with drug hypersensitivities should be educated on avoiding the medication and charts must appropriately document the reaction. Some antibiotics are known to be associated with hypersensitivity, but no study has systemically compared hypersensitivity associations for many available antibiotics. The objective of this study was to evaluate the association between hypersensitivity and antibiotics using the FDA Adverse Event Reporting System (FAERS).

**Methods:** FAERS reports from January 1, 2004, to December 31, 2022 were included in the study. The Medical Dictionary for Regulatory Activities (MedDRA) was used to identify hypersensitivity cases. Reporting odds ratios (RORs) and corresponding 95% confidence intervals (95% CI) for the association between antibiotics and hypersensitivity were calculated. ROR was calculated as the ratio of the odds of reporting hypersensitivity versus all other events for a given drug compared with this reporting odds for all other drugs present in FAERS. An association was considered to be statistically significant when the lower limit of the 95% CI was greater than 1.0.

**Results:** A total of 16,009,712 reports (including 144,714 hypersensitivity reports) were considered, after inclusion criteria were applied. 72 antibiotics were evaluated, and 39 of those antibiotics were significantly associated with hypersensitivity. The top 10 antibiotics with the highest number of hypersensitivity reports were trimethoprim-sulfamethoxazole (2,550), amoxicillin (1,483), ciprofloxacin (1,327), moxifloxacin (1,253), levofloxacin (1,170), azithromycin (1,039), amoxicillin-clavulanate (1,012), doxycycline (840), metronidazole (822), and clarithromycin (776). The top 10 antibiotics with the highest hypersensitivity ROR (95% CI) were penicillin G 8.17 (7.45-8.96), dalbavancin 6.78 (5.00-9.19), cefaclor 5.38 (4.40-6.57), moxifloxacin 4.62 (4.36-4.89), oritavancin 4.14 (2.89-5.92), penicillin V 3.79 (3.35-4.30), ceftibuten 3.62 (2.04-6.44), bacitracin 3.43 (2.88-4.10), cefprozil 3.35 (2.37-4.74), and erythromycin 3.22 (2.94-3.51).

**Conclusions:** Out of the 72 antibiotics evaluated in the FAERS database, 39 were significantly associated with hypersensitivity. Trimethoprim-sulfamethoxazole had the highest reported number of hypersensitivity reactions followed by amoxicillin, ciprofloxacin, moxifloxacin, levofloxacin, azithromycin, amoxicillin-clavulanate, doxycycline, metronidazole, and clarithromycin. Penicillin G had the highest ROR with hypersensitivity followed by dalbavancin, cefaclor, moxifloxacin, oritavancin, penicillin V, ceftibuten, bacitracin, cefprozil, and erythromycin.

## P6: Improving cost-effectiveness for data migration in healthcare environments using serverless architecture

### Prashant Duhoon, Can Ersoy, Neset Hikmet

#### Department of Integrated Information Technology, College of Engineering and Computing, University of South Carolina, Columbia, SC, USA

##### **Correspondence:** Prashant Duhoon (duhoon@mailbox.sc.edu)

*BMC Proceedings 2024,* **18(8):**P6

**Issues:** The popularity of serverless technologies and serverless architecture is currently at an all-time high across various industries because of their ability to effectively address the constantly evolving demands of scalability, cost-efficiency, and security. Verified market research indicates that the serverless architecture market is projected to experience a growth of 22.7%, increasing from USD 7,585 million in 2020 to USD 21,105 million by 2025 [1]. This is particularly true in healthcare, as it is a data-driven field. Implementing solutions that can enhance the quality of patient care and operational efficiency is of crucial importance. Exploring these solutions is only possible with the necessary observational tools or environment to effectively work with the data. In this paper, our objective is to extend the applicability of the serverless architecture implemented in a current research project. We aim to generalize its usage across various industries and scenarios, encompassing tasks such as data gathering, ETL processes, data storage, data security, and in the end providing an environment to conduct certain studies with the data.

**Project:** The South Carolina Center for Center for Effectiveness Research in Orthopedics (CERortho) is a joint initiative involving the University of South Carolina and Prisma Health which aim to expand the comparative effectiveness research in the field of orthopedic care. A serverless architecture was designed and implemented to facilitate the creation of the Orthopedic Patient Data Repository (OPDR) and provide an observational environment for the researchers to conduct research, analysis, and development within this repository.

The system operates in a HIPAA compliant AWS (Amazon Web Services) Virtual Private Cloud and the process has been decoupled from each other into different modules. An AWS SFTP transfer instance automates data transfer from Prisma Health EPIC crystal reports into HIPAA compliant S3 buckets with AES 256 encryption at rest and TLS (Transport Layer Security) 2.0 in motion. The movement and access of data is logged and monitored for audits.

Once this pipeline is defined, Extract Transform Load (ETL) is performed using AWS Glue jobs. Glue allows for extracting valuable information from S3 buckets, transforming the data by setting up rules for de-identifying Personally Identifiable Information (PII) and Protected Health Information (PHI) in patient data, and load the extracted and transformed dataset into databases. SQL databases are used in the instances for datasets requiring recreating relationships and NoSQL databases are used for all other kinds of datasets. These databases are themselves hosted on serverless services using DynamoDB and RDS on AWS. Upon requests received by an honest data broker for specific datasets, the data is securely transferred to the local research instance using AWS DataSync. The research server itself is configured to restrict file transfers, clipboard access and certain user read, write, and modify permissions. As a result, an automated end-to-end all serverless data migration system that requires minimal human intervention has been deployed and proven to be highly cost effective and secure. A figure (1) has been provided as a reference to the above.

**Lessons Learned:** Demonstrating cost-effectiveness of serverless data migration in healthcare environments over server environment (Physical and Virtual) has been the highlight of the project. The team demonstrated improved latencies, security, scalability, and availability of the system with reduced costs incurred for data migration between healthcare providers and research environments. Challenges remain in terms of acquiring architects and developers for enhancement and maintenance of the system. The team plans to create a training program to bridge some of the gaps in skillsets.

**Reference**

1. “Research and Markets Offers Report: Serverless Architecture Market.” Entertainment Close-up. June 30, 2020. Gale In Context: Biography. Available from: link.gale.com/apps/doc/A628073078/BIC?u=colu68650&sid=bookmark-BIC&xid=4a9bbc62.

**Fig. 1 (Abstract P6):**
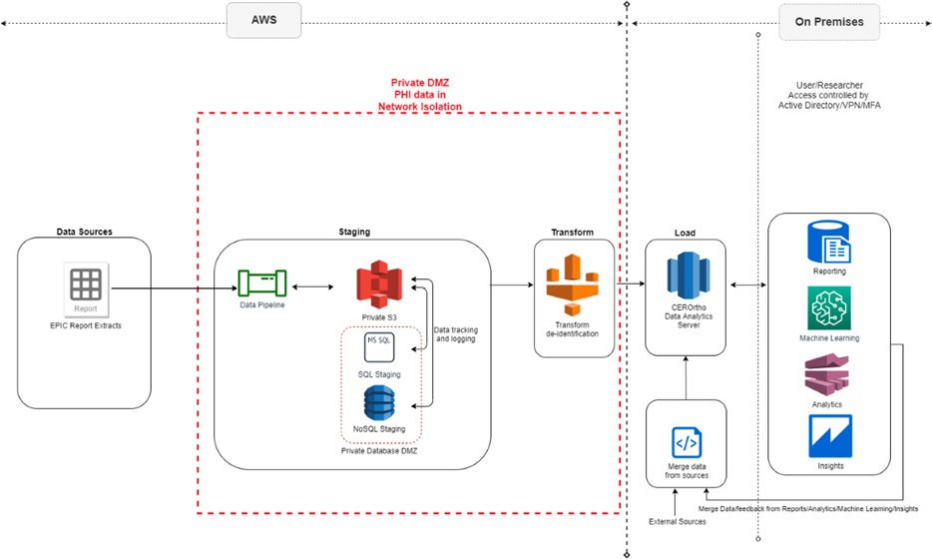
A Serverless architecture for healthcare data migration

## P7: Divergence and intersection of practical and implementable compliance with HIPAA’s Privacy Rule and the FTC’s Health Breach Notification Rule: a case study

### Marilyn Gartley

#### Department of Computer Science and Engineering, College of Engineering and Computing, University of South Carolina, Columbia, SC, USA

##### **Correspondence:** Marilyn Gartley (gartley@email.sc.edu)

*BMC Proceedings 2024,* **18(8):**P7

**Background**

We live in an age where the data practices of today’s surveillance economy can create and exacerbate deep asymmetries of information, which in turn exacerbate imbalances of power—including in the healthcare arena [1–3]. Tools using artificial intelligence (AI) techniques find patterns in big health data, which in turn can be used to make predictions and recommendations in healthcare and thereafter, these new insights are routinely incorporated back into the clinical care pathway—explicitly through practice guidelines or publications or implicitly in the context of recommendations or procedures automatically embedded into electronic health record (EHR) systems [4].

**Case report**

Medical AI applications are particularly data hungry and depend on continued collections of feature-rich consumer health data, where not only are the most valuable datasets those most prone to reidentification but also where such collections and retentions routinely expand beyond their originally intended and subsequent uses [5]. A direct conflict with data minimization as a fundamental principle of data privacy law [6] and as a key protection of the HIPAA Privacy Rule [7-8] is presented by AI applications in that data maximization, rather than minimization, is a central success factor for AI [9].

**Conclusion**

This paper will approach this issue by proposing written procedures to provide for realizable implementations beyond deidentification to facilitate the secure disclosures and exchanges of information held, used, or exchanged by a covered entity or business associate that is deemed not individually identifiable in adherence to the Privacy Rule [10] while avoiding information blocking [10]. Finally, this paper will provide a framework of key questions to analyze in determining whether an entity constitutes a “PHR related entity,” “Third party service provider,” or “Vendor of personal health information” subject to the Health Breach Notification Rule with a focus on elucidating the scope of prohibited disclosures considered to have been made “without the authorization of the individual” [11,12]. In turn, this paper will directly examine what it means to fall within the scope of an app covered by the Health Breach Notification Rule when collecting information directly from consumers with the technical capacity to draw information through an API that enables syncing with a consumer’s fitness tracker [12,13].

**References**

1. Federal Trade Commission (FTC), Statement of Chair Lina M. Khan Regarding the Commercial Surveillance and Data Sec. Advance Notice of Proposed Rulemaking Comm’n File No. R111004 (Aug. 11, 2022).
2. Gianfrancesco M, Tamang S, Yazdany J, Schmajuk G, Potential Biases in Machine Learning Algorithms using Electronic Health Record Data, JAMA Internal Medicine 178(11), 1544–47 (2018), 10.1001/jamainternmed.2018.3763.
3. Obermeyer Z, Powers B, Vogeli C, Mullainathan S, Dissecting Racial Bias in an Algorithm used to Manage the Health of Populations, Science 366, no. 6464, 447–53 (Oct. 2019), 10.1126/science.aax2342.
4. Nicholson W, Cohen IG, Privacy in the Age of Medical Big Data, Nature Medicine 25(1), 37 (2019), 10.1038/s41591-018-0272-7.
5. Cranor L, Frischmann B, Harkins R, Nissenbaum H, Panel I: Disclosure and Notice Practices in Private Data Collection, Cardozo Arts & Entertainment Law Journal 32(3), 784–812, 807 (2014).
6. FTC, Mobile Health App Developers: FTC Best Practices, available at: https://www.ftc.gov/business-guidance/resources/mobile-health-app-developers-ftc-best-practices; HHS, Use of Online Tracking Technologies by HIPAA Covered Entities and Business Associates (Dec. 1, 2022).
7. 45 CFR 164.502(b), 164.514(d) & Office of Civil Rights, Minimum Necessary OCR HIPAA Privacy Paper (revised April 4, 2003), available at: https://www.hhs.gov/sites/default/files/ocr/privacy/hipaa/understanding/coveredentities/minimumnecessary.pdf.
8. Office for Civil Rights, Collection, Use, and Disclosure Limitation, The HIPAA Privacy Rule and Elect.Health Info. Exch. in a Networked Env’t Series, available at: https://www.hhs.gov/sites/default/files/ocr/privacy/hipaa/understanding/special/healthit/collectionusedisclosure.pdf.
9. Tschider C, The HealthCare Privacy-Artificial Intelligence Impasse, Santa Clara Computer & High-Tech. L. J. 36(4), 439–43 (2020).
10. 45 C.F.R. §§ 160.103; 164.502; 164.514; Nat’l Comm. on Vital and Health Statistics, Recommendations on De-identification of Protected Health Information under HIPAA (Feb. 23, 2017); Health Information Tech. Advisory Comm. (HITAC), HITAC Annual Report for Fiscal Year 2019 (Feb. 19, 2020).
11. FTC, Health Breach Notification Rule, Notice of Proposed Rulemaking, Fed. Reg. vol. 88(11) (June 9, 2023) & 16 C.F.R. §§ 318.1 to 318.9.
12. U.S. v. Easy Healthcare Corp., Case No. 1:23-cv-3107 (N.D. Ill.); U.S. v. GOODRX Holdings, Inc., Case No. 3:23-cv-460 (N.D. Cal.); In the Matter of 1Health.io/Vitagene, FTC Docket No. C-4798; In the Matter of Flo Health, Inc., FTC Docket No. C-4747.
13. FTC, FTC Warns Health Apps and Connected Device Companies to Comply with Health Breach Notification Rule, Press Release (Sept. 15, 2021), available at: https://www.ftc.gov/news-events/press-releases/2021/09/ftc-warns-health-apps-connected-device-companies-comply-health; FTC, Statement of the Comm’n on Breaches by Health Apps and Other Connected Devices (Sept. 15, 2021), available at: https://www.ftc.gov/system/files/documents/public_statements/1596364/statement_of_the_commission_on_breaches_by_health_apps_and_other_connected_devices.pdf.

## P8: Visualizing major healthcare breaches

### Sharon Gumina

#### Department of Integrated Information Technology, College of Engineering and Computing, University of South Carolina, Columbia, SC, USA

##### **Correspondence:** Sharon Gumina (gumina@mailbox.sc.edu)

*BMC Proceedings 2024,* **18(8):**P8

**Objectives:** My objectives in this study were to use data visualizations to identify healthcare breach trends and contrast those trends to other industry sectors. I am motivated to analyze this data because the healthcare sector has experienced a surge in ransomware attacks which disrupt the ability of healthcare organizations to provide quality medical care and breach the confidentiality of electronic health records [1]. A ransomware attack occurs when malicious software allows threat actor(s) to restrict access to vital information and then demand some form of payment to lift the restriction. In 2023, ransomware comprised twenty four percent of all breaches across all industries and organization sizes [1].

**Methods:** Companies holding medical record information are required to report any major data breach affecting more than five hundred healthcare records to the U.S. Department of Health and Human Services (DHHS) Office for Civil Rights’ Breach Portal [2]. Using an open-source dataset that contained the DHHS portal’s breach information, I was able to create interactive longitudinal visualizations that depict the millions of individuals affected by these healthcare breaches from 2009 until 2019 [3].

**Results:** The area chart shows an increase in healthcare breaches from 2009 until 2019 with a large spike in 2015 due to a massive breach at Anthem, Inc. in Indiana in 2015 that affected more than 80 million individuals [2] (Figure 1).

Using another open-source dataset available on Kaggle.com that contained over 1000 ransomware breach records [4], I was able to use longitude and latitude of organizations that reported ransomware attacks from 2019 until 2021 using a geospatial visualization (Figure 2).

The locations are color coded according to industry categories that include business, education, government, and healthcare.

**Discussion:** Longitudinal and geospatial visualizations are powerful tools in understanding the impact of ransomware across the US within healthcare and help identify trends including the spike in 2015 when over 80 million medical records were breached at one insurance company in Indiana [2].

These visualizations show an increasing cyber threat to healthcare organizations and patients in the US with ransomware attacks as one of the most disastrous types of attacks because it may disrupt healthcare delivery and put patients at risk.

Currently, there is no systematic documentation of the extent and effect of ransomware attacks so more longitudinal and geospatial analyses of these healthcare ransomware attacks would be vital to understanding the nationwide impact of ransomware on US healthcare organizations and individuals [5].

**References**

1. DBIR report 2023 - summary of findings [Internet]. [cited 2023 Dec 4]. Available from: https://www.verizon.com/business/resources/reports/dbir/2023/summary-of-findings/
2. John Russell IBJ. Indiana leads the nation in medical data breaches, report says [Internet]. 2022 [cited 2023 Dec 4]. Available from: https://www.insideindianabusiness.com/articles/indiana-leads-the-nation-in-medical-data-breaches-report-says
3. Healthcare data breaches - project by Nicole-Mark [Internet]. 2021 [cited 2023 Dec 4]. Available from: https://data.world/nicole-mark/healthcare-data-breaches
4. ransomware-attacks [Internet]. www.kaggle.com. [cited 2023 Dec 4]. Available from: https://www.kaggle.com/datasets/samara2022/ransomware-attack
5. Neprash HT, McGlave CC, Cross DA, Virnig BA, Puskarich MA, Huling JD, Rozenshtein AZ, Nikpay SS. Trends in ransomware attacks on US hospitals, clinics, and other health care delivery organizations, 2016-2021. In JAMA Health Forum 2022 Dec 2 (Vol. 3, No. 12, pp. e224873-e224873). American Medical Association.

**Fig. 1 (Abstract P8):**
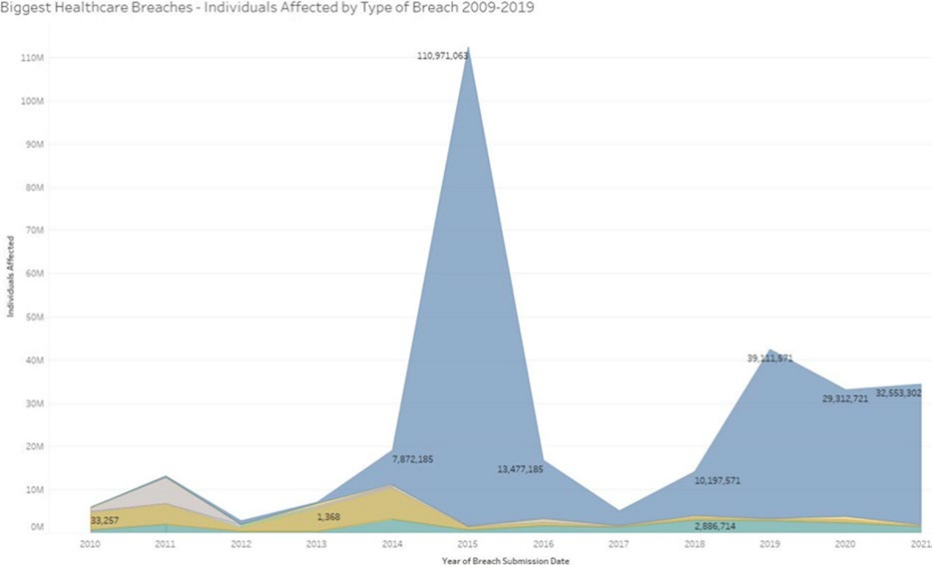
Individuals Affected by Type of Breach 2009-2019

**Fig. 2 (Abstract P8):**
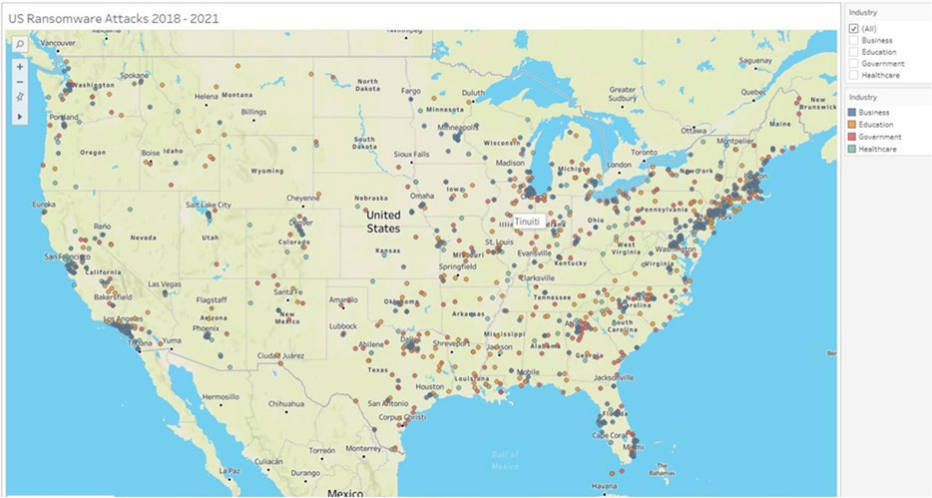
Location of Ransomware Attacks 2018-2021

## P9: The benefits of electronic case reports for reportable disease tracking by public health agencies

### Margaret Iiams, Abdoulaye Diedhiou, Katie Platt, John Sinclair, Claire Youngblood

#### ^1^South Carolina Department of Health and Environmental Control, Columbia, SC, USA

##### **Correspondence:** Margaret Iiams (iiamsm@dhec.sc.gov)

*BMC Proceedings 2024,* **18(8):**P9

**Study Objectives:** The Division of Acute Disease Epidemiology of the South Carolina Department of Health and Environmental Control (DHEC) tracks cases of reportable diseases. Multiple methods exist for cases to be reported to the department, some of which require manual reporting by healthcare providers or organizations. One issue seen with manual reporting methods is missing or incomplete data. Electronic case reports (eCRs) are a method of reporting diseases that comes directly from the patient’s electronic health record. This method does not require manual reporting and has the potential to decrease the occurrence of missing or incomplete data as well as supplying data not usually provided by other reporting methods.

**Methods:** The state of South Carolina DHEC went live for certain eCRs on October 2^nd^, 2023. This study looked at the eCRs that came through for covid cases from a limited set of healthcare organizations. The completion of the data was calculated and compared to other reporting methods.

**Results:** Of the cases that contain data from an eCR so far, all of them contained contact information for the healthcare provider and region information for the patient. In addition, most of them have a known race listed.

**Discussion:** Based on the initial findings, the eCRs that were received were effective at increasing the completion percentage of race data and providing contact and region data to DHEC. This information is vital for the department in its efforts to track diseases in the state. Complete race data is essential for understanding and promoting health equity.

## P10: Utility of South Carolina’s statewide telephone helpline in COVID-19 prevention and control

### Megha Oza^1^, Prema S. Bhattacharjee^2^, Emmanuel Fabrice Julceus^2^, Joseph Robert^3^, Cheryl L. Scott^4^

#### ^1^Department of Environmental Health Sciences, Arnold School of Public Health, University of South Carolina, Columbia, SC, USA; ^2^Department of Epidemiology and Biostatistics, Arnold School of Public Health, University of South Carolina, Columbia, SC, USA; ^3^Centers for Disease Control and Prevention (CDC) Foundation, CDC, Atlanta, GA, USA; ^4^Community Health Services Bureau, South Carolina Department of Health and Environmental Control, Columbia, SC, USA

##### **Correspondence:** Emmanuel Fabrice Julceus (ejulceus@email.sc.edu)

*BMC Proceedings 2024,* **18(8):**P10

**Study Objectives:** Several US states utilized telephone helplines/hotlines for COVID-19 management during 2020-2021. The South Carolina Department of Health and Environmental Control (SCDHEC) expanded use of its helpline, Care Line, to receive and respond to COVID-19 concerns. This study aims to examine the usefulness of Care Line’s COVID-19 data in prevention and control.

**Methods:** This retrospective analysis of the Care Line COVID-19 dataset concerns calls received during May 2020 – April 2021. Call variables included number of calls, reason for call, region, county, age, and gender. COVID-19 cases, hospitalizations, deaths, and socioeconomic variables were abstracted from SCDHEC and other publicly available databases. Correlation between Care Line calls and COVID-19 cases, hospitalizations and deaths, and association of reason for call, number of calls and sociodemographic variables were evaluated using Pearson correlation, cross-correlation, Poisson regression, and chi-square test.

**Results:** Care Line received 119,721 COVID-19-related calls during the study period. There was a positive correlation between distribution of weekly Care Line calls and weekly COVID-19 cases (*r*= 0.71, *p*<0.0001), hospitalizations (*r*= 0.61, *p*<0.0001), or deaths (*r*= 0.70, *p*<0.0001), and call peaks preceded peaks in cases, hospitalizations, and deaths. The best match lag between Care Line calls and COVID-19 cases was two weeks. Call rates in Midlands (1.40, 95% CI 1.07-1.84) and Pee Dee regions (1.45, 95% CI 1.11-1.90) were significantly higher than rates in Upstate. Younger callers and callers from lower income counties or with a lower proportion of non-Hispanic White residents called more regarding testing and less regarding vaccines than their counterparts.

**Discussion:** The overall trend of Care Line COVID-19 calls is correlated closely to cases and describes sociodemographic patterns comparable to COVID-19 cases and vaccinations in South Carolina. The utility of Care Line COVID-19 data in prevention and control appears its early surveillance potential to correlate with COVID-19 cases and vaccination trends in South Carolina.

**Keywords:** Helpline; COVID-19; Call trends; Surveillance; Testing; Vaccination

## P11: Building a dashboard: visualizing South Carolina state health department Cancer Programs efforts

### Jaron King^1,2^, Katelyn Hastie^1,2^

#### ^1^Cancer Programs, South Carolina Department of Health and Environmental Control, Columbia, SC, USA; ^2^Department of Health Promotion, Education, and Behavior, Arnold School of Public Health, University of South Carolina, Columbia, SC, USA

##### **Correspondence:** Jaron King (kingjh@dhec.sc.gov)

*BMC Proceedings 2024,* **18(8):**P11

**Issues:** The South Carolina (SC) Cancer Programs is a new operational unit within the Bureau of Chronic Disease and Injury Prevention at the SC state health department (SC DHEC). The Cancer Programs unit is a unified division that brings all 3 arms of the more than $6.5m CDC-funded Cancer Prevention and Control grant together into a single unit: 1) SC Central Cancer Registry (SCCCR), 2) SC Breast and Cervical Cancer Early Detection Program, Best Chance Network (BCN), and 3) SC Comprehensive Cancer Control Program (“Comp.” Cancer). The Cancer Programs sought a unified system to measure and visualize ongoing efforts.

**Project**: The SC Cancer Programs has upwards of 50 staff members whose roles vary from Data Integrity Analysts, who process tens of thousands of claims annually, to Certified Tumor Registrars who verify all new incidences of and treatments for cancer in the state. According to National Cancer Institute and SCCCR data, there are around 33,000 new cases of cancer diagnosed in SC each year. The near-constant physical, economic, and emotional toll cancer takes on the public calls for efforts that spread across various activities and initiatives. While creating a dashboard to track activities, we identified more than 30 unique activities performed by Cancer Programs staff and an additional six data sources to measure the effectiveness of efforts. The dashboard was created to be updated monthly with unique activity measures and annually or semi-annually with new cancer incidence and mortality data. We used PowerBI to create a dashboard that reflected thousands of unique data points from cancer screenings and treatments to lives touched by public health efforts.

**Lessons Learned**: The dashboard created for the SC Cancer Programs is an example of a hybrid data visualization and tracking tool that can be used and replicated by other areas of public health as well as other states with similar cancer program infrastructure. As is common when creating new digital tools, this project took longer than initially anticipated, however some of the lessons learned included enhanced resources and communication across the cancer programs. While multiple yearly reports were generated regularly to track the progress and impact of cancer programming, the dashboard now allows leadership and evaluators to quickly see how individual teams are performing in the goals and objectives they have set to reduce the impact of cancer on South Carolinians.

## P12: An exploration of lag in payer ascertainment among maternal delivery claims in South Carolina: potential implications for timely maternal care

### Linga Murthy Kotagiri, Sarah Gareau, Ana López-De Fede

#### Institute for Families in Society, College of Social Work, University of South Carolina, Columbia, SC, USA

##### **Correspondence:** Linga Murthy Kotagiri (kotagiri@mailbox.sc.edu)

*BMC Proceedings 2024,* **18(8):**P12

**Background:** The University of South Carolina Institute for Families in Society (IFS) under the auspices of the South Carolina Birth Outcomes Initiative Data Workgroup has reported maternal and newborn health quality trends since 2011. Medicaid is the largest payer of deliveries in SC (62%). Starting in 2021, IFS identified a reduction in the rate of Medicaid coverage by 2-3%, resulting in the need to restrict public reporting by 12 months. Big data management techniques were used to identify the root cause of delay in payer ascertainment as data linkage timing to the Medicaid eligibility file.

**Objective:** As part of providing hospital end users more timely data to aid obstetric quality improvement efforts, we investigated in collaboration with South Carolina Revenue and Fiscal Affairs (SCRFA) decreasing Medicaid rates and the patients impacted by it.

**Materials and Methods:** IFS receives on a quarterly basis from SCRFA all hospital records for any SC delivery patient for 18 months prior to, at the time of delivery, and 18 months after delivery (over a million total records since October 1, 2015). These data are linked to all birth records to derive birth outcomes and key demographics and to Medicaid eligibility to confirm payer.

A single quarter’s delivery UB-04 claims, Q4, 2021 (October – December), were compared across two data time periods: 6-months and 18-months out from the date of service. The later data represented results after testing a fix to maximize Medicaid match across all eligibility files for mothers and newborns. The study compared Medicaid eligibility, age, race, rurality, high social vulnerability (HSV), low birthweight, prematurity, or having a diagnosis of COVID-19 or a chronic physical or behavioral health condition during the 12-months prior to or at the time of delivery using the test for equality of two proportions.

**Results:** The later data showed a 2% increase in Medicaid deliveries confirming the lag in ascertainment of Medicaid as the final payer (61.6% vs 59.5%, *p*<0.05). Compared to all deliveries, lagged Medicaid deliveries had a significantly (*p*<0.05) higher percentage of ages 20-24 years (36% vs 22%), Hispanic ethnicity (20% vs 12%), HSV (25% vs 19%), low birthweight (16% vs 10%) or prematurity (16% vs 11%), COVID-19 (10% vs 5%), and limited benefit (14% vs 11%) or Fee-for-Service coverage (30% vs 17%). Though not significant, lagged Medicaid deliveries also had a higher percentage of avoidable cesareans (30% vs 26%), rurality (29% vs 26%), and chronic physical (30% vs 26%) or behavioral health conditions (24% vs 23%).

**Conclusions:** Given this data lag could result in missed opportunities to identify complex delivery patients for early intervention, we prioritized working with SCRFA to permanently improve data linkages to Medicaid eligibility resulting in nearly complete records as of October 2023 with public reporting now only six months from the date of delivery. The volume of data, linkage across three data sources, and ongoing data has enabled obstetric providers to access real-time robust data for quality improvement. This investigation has ensured that some of the most highly vulnerable delivery patients are now represented.

## P13: Exploring disparities in exposure to fine particulate matter (PM2.5) according to social vulnerability in South Carolina from 2000-2018

### Erin N. Looney^1^, Shirelle H. Hallum^1^, Anna L. Chupak^1^, Kelsey M. Thomas^1^, Dwayne E. Porter^2^, Yesil S. Kim^3^, Andrew T. Kaczynski^1,3^

#### ^1^Department of Health Promotion, Education, and Behavior, Arnold School of Public Health, University of South Carolina, Columbia, SC, USA; ^2^Department of Environmental Health Sciences, Arnold School of Public Health, University of South Carolina, Columbia, SC, USA; ^3^Prevention Research Center, Arnold School of Public Health, University of South Carolina, Columbia, SC, USA

##### **Correspondence:** Erin N. Looney (looneye@email.sc.edu)

*BMC Proceedings 2024,* **18(8):**P13

**Study Objectives:** Air pollution in the US has declined since the 1970s, but disparities in exposure to harmful air pollutants like fine particulate matter (PM2.5) persist. Previous research has examined associations between air pollution exposure and individual factors such as socioeconomic status or race/ethnicity but seldom explored social vulnerability or temporal trends in South Carolina (a state characterized by significant health issues and inequities). The purpose of this study was to investigate the relationship between PM2.5 and overall social vulnerability in SC from 2000-2018.

**Methods:** The CDC/ATSDR Social Vulnerability Index (SVI) was used for all census tracts in SC (*n*=867 as of 2000) based on four components: minority status and language (comprised of two variables), socioeconomic status (four variables), housing type and transportation (five variables), and household composition and disability (four variables). Overall SVI was categorized into tertiles (low=289, moderate=290, high=288). North American estimates of annual surface PM2.5 concentration (μg/m3) from Washington University in St. Louis were ascertained for each tract from 2000 through 2018. A repeated measures ANOVA was used to analyze trends and disparities in PM2.5 for low, moderate, and high SVI tracts (controlling for tract urbanicity).

**Results:** Tracts at the low, moderate, and high vulnerability levels observed respective PM2.5 decreases of 48.1%, 49.1%, and 48.3% from 2000 to 2018. A significant change in mean PM2.5 occurred over the 19-year study period (F=24494.3, *p*<.0001), with a significant tract by SVI tertile interaction (F=3.49, *p*<.0001). Annual PM2.5 differences relative to the 2000 baseline were significant in six of the 19 years analyzed. High-vulnerability tracts consistently exhibited higher average annual PM2.5 concentrations than their moderate and low-vulnerability counterparts, except in 2012 and 2016. Statistically significant findings were also observed for the SVI components.

**Discussion:** The results of this study revealed a significant reduction in average annual PM2.5 concentrations across all levels of social vulnerability (low, moderate, high) from 2000 to 2018. Further, a significant interaction between tract and SVI tertiles indicated that the change in PM2.5 concentrations differed according to social vulnerability level, suggesting that the impact of air quality improvements on communities was not uniform. These findings underscore the ongoing need for environmental justice efforts and highlight the complex interplay between social factors and air quality trends.

*This abstract was selected as the Best Poster Presentation from an Emerging Scholar in the Poster Session 2*

## P14: Understanding the influence of health insurance and medical home environments on healthcare utilization for children with autism spectrum disorder: a logistic regression analysis of NSCH 2016-2021

### Gahssan Mehmood, Jan Ostermann, Ibitein Okeafor

#### Department of Health Services Policy and Management, Arnold School of Public Health, University of South Carolina, Columbia, SC, USA

##### **Correspondence:** Gahssan Mehmood (gmehmood@email.sc.edu)

*BMC Proceedings 2024,* **18(8):**P14

**Background:** Children diagnosed with autism spectrum disorder (ASD) face unique healthcare challenges owing to the diverse nature of the condition. ASD is characterized by a spectrum of medical, developmental, and behavioral needs, often requiring specialized and comprehensive healthcare services. The healthcare needs of this population extend beyond traditional medical services, encompassing behavioral and developmental support to enhance their overall well-being. In this context, the concept of a medical home emerges as a crucial component in delivering holistic care for children with ASD. A medical home refers to a centralized, family-centered approach to healthcare that provides coordinated and continuous care for individuals. This study delves into the intricate dynamics surrounding healthcare utilization for children aged 3 to 17 years with ASD in the United States. Understanding these patterns is essential for addressing the specific needs of this population and improving overall healthcare equity.

**Objectives:** The primary objective of this research is to examine the impact of the interaction between consistent and adequate health insurance coverage and access to a comprehensive medical home environment on various healthcare utilization outcomes, including medical care, emergency room (ER) visits, preventive care utilization, mental healthcare, and instances of forgone healthcare.

**Methods:** Logistic regression models were applied to analyze adjusted odds ratios (AOR) and 95% confidence intervals (CI) using data from the National Survey of Children’s Health (NSCH) spanning the years 2016 to 2021. The NSCH dataset, a nationally representative survey, provided a robust foundation for exploring the multifaceted relationships within the diverse population of children with ASD. In our sample, comprising 5,489 children with ASD weighted to a population of 1,652,862, the logistic regression models considered a range of control variables, ensuring a nuanced and comprehensive analysis.

**Results:** The findings offer valuable insights into the complex interplay of health insurance coverage and medical home environments on healthcare utilization outcomes (Table 1). Children with inconsistent health insurance coverage but residing in a comprehensive medical home environment exhibited increased odds of medical care utilization (AOR=1.64, 95% CI: 1.01-2.67). Notably, older children in the 15-17 age group demonstrated reduced odds of preventive care utilization compared to their younger counterparts (3-7 years) (AOR=0.34, 95% CI: 0.22-0.54), highlighting potential gaps in age-specific healthcare interventions for this population. Instances of forgone healthcare were notably associated with inconsistent health insurance coverage and the absence of a comprehensive medical home environment (AOR=3.43, 95% CI: 1.66-7.12). This suggests that the lack of consistent coverage coupled with the absence of a supportive medical home environment may contribute to challenges in accessing necessary healthcare services, emphasizing the need for targeted interventions to mitigate these barriers.

**Discussion & Conclusion:** The logistic regression analysis provides a comprehensive understanding of healthcare utilization patterns among children with ASD. These findings underscore the importance of consistent health insurance coverage and the presence of a comprehensive medical home environment in facilitating adequate healthcare access and utilization. Tailoring interventions to address age-specific healthcare needs and ensuring consistent coverage and supportive medical home environments are crucial for enhancing healthcare equity and improving overall well-being for this vulnerable population.

**Keywords:** Healthcare utilization; Health insurance; Medical Home; preventive care; medical care; children

**Table 1 (Asbtract P14):** Multivariable logistic regression estimates

	Medical care	ER	Preventive care	Mental Healthcare	Forgone health care
	Adjusted AOR (95% CI)	Adjusted AOR (95% CI)	Adjusted AOR (95% CI)	Adjusted AOR (95% CI)	Adjusted AOR (95% CI)
Health insurance coverage and medical home environment comprehensiveness (Ref: Consistent coverage & comprehensive medical home environment)					
Inconsistent coverage but comprehensive medical home environment	1.64 (1.01-2.67)	1.27 (0.82-1.98)	1.46 (0.94-2.26)	1.23 (0.86-1.76)	1.54 (0.75-3.17)
Consistent coverage but no comprehensive medical home environment	0.71 (0.40-1.27)	0.79 (0.45-1.39)	0.64 (0.37-1.10)	1.03 (0.65-1.61)	0.79 (0.36-1.75)
Inconsistent coverage & no comprehensive medical home environment	0.93 (0.55-1.58)	0.89 (0.58-1.38)	0.68 (0.43-1.07)	0.98 (0.68-1.43)	3.43 (1.66-7.12)
Income Status (Ref: 0-99%)					
100%-199% FPL	1.06 (0.65-1.74)	0.62 (0.37-1.02)	0.71 (0.43-1.18)	0.80 (0.51-1.25)	1.16 (0.64-2.10)
200%-399% FPL	1.02 (0.61-1.68)	0.68 (0.43-1.07)	0.82 (0.50-1.34)	0.61 (0.41-0.91)	0.89 (0.52-1.52)
400% FPL or greater	1.01 (0.58-1.75)	0.49 (0.30-0.80)	0.78 (0.45-1.33)	0.69 (0.45-1.05)	0.43 (0.23-0.80)
Race and Ethnicity of Child (Ref: White)					
Black	0.46 (0.27-0.76)	1.02 (0.69-1.50)	0.63 (0.40-1.01)	0.71 (0.49-1.03)	0.75 (0.39-1.45)
Hispanic	0.48 (0.30-0.78)	0.70 (0.42-1.18)	0.69 (0.45-1.06)	0.76 (0.53-1.11)	1.29 (0.68-2.47)
Others	0.65 (0.39-1.06)	0.68 (0.46-1.01)	0.86 (0.55-1.34)	0.78 (0.58-1.06)	0.97 (0.61-1.55)
Age of child (Ref: 3 - 7 years old)					
8-11 years old	0.65 (0.39-1.09)	0.99 (0.68-1.46)	0.53 (0.33-0.83)	1.21 (0.84-1.75)	0.99 (0.66-1.49)
12-14 years old	0.70 (0.39-1.27)	1.24 (0.80-1.92)	0.72 (0.43-1.21)	1.81 (1.24-2.66)	1.45 (0.84-2.49)
15-17 years old	0.31 (0.19-0.53)	1.01 (0.71-1.43)	0.34 (0.22-0.54)	2.15 (1.53-3.03)	1.80 (1.10-2.95)
Child Sex (Ref: Female)					
Male	0.77 (0.49-1.21)	0.92 (0.65-1.28)	1.10 (0.72-1.66)	0.91 (0.67-1.23)	1.09 (0.73-1.63)
Out-of-Pocket Cost for Childcare (Ref: Less than 250$)					
$250 - $499	1.03 (0.57-1.85)	0.81 (0.52-1.26)	0.98 (0.59-1.63)	0.79 (0.54-1.14)	1.08 (0.51-2.29)
$500 - $999	1.69 (0.88-3.27)	1.12 (0.70-1.77)	1.37 (0.76-2.48)	1.73 (1.18-2.54)	0.82 (0.42-1.59)
More than $1000	2.60 (1.43-4.71)	1.46 (1.01-2.11)	2.33 (1.39-3.92)	2.26 (1.63-3.12)	1.75 (1.07-2.87)
Household Language (Ref: English)					
Spanish/Other	0.43 (0.22-0.84)	1.16 (0.61-2.20)	0.48 (0.27-0.88)	0.78 (0.39-1.58)	0.22 (0.10-0.51)
Family Resilience (Ref: More Resilient Family)					
Less Resilient Family	0.93 (0.62-1.40)	1.04 (0.75-1.46)	0.76 (0.53-1.08)	1.07 (0.81-1.41)	1.36 (0.96-1.93)
Supportive neighborhood (Ref: Yes)					
No	1.63 (1.10-2.41)	1.32 (0.98-1.77)	1.31 (0.94-1.81)	0.96 (0.74-1.24)	1.57 (1.03-2.40)
Highest level of education of any adult in the household (Ref: College degree or higher)					
Less than high school	1.25 (0.55-2.85)	1.80 (0.85-3.80)	0.71 (0.34-1.51)	1.00 (0.51-1.95)	0.53 (0.20-1.43)
High school degree or GED	0.61 (0.38-1.00)	1.20 (0.80-1.79)	0.66 (0.42-1.02)	1.14 (0.78-1.67)	0.89 (0.47-1.68)
Some college or technical school	0.81 (0.52-1.27)	1.34 (0.94-1.92)	0.86 (0.59-1.27)	1.43 (1.07-1.92)	0.90 (0.58-1.39)
Rurality ( Ref : Urban)					
Rural	1.19 (0.73-1.93)	1.17 (0.81-1.70)	1.09 (0.73-1.65)	0.77 (0.57-1.05)	0.99 (0.62-1.59)
Missing	0.83 (0.51-1.36)	1.15 (0.83-1.61)	0.83 (0.55-1.26)	0.93 (0.70-1.22)	1.04 (0.65-1.65)
Region (Ref: West)					
Northeast	0.68 (0.39-1.20)	1.01 (0.65-1.56)	1.13 (0.69-1.85)	1.79 (1.23-2.61)	0.73 (0.38-1.39)
Midwest	0.86 (0.50-1.48)	1.01 (0.67-1.53)	1.57 (0.97-2.52)	1.18 (0.82-1.69)	0.57 (0.35-0.93)
South	0.68 (0.42-1.12)	0.92 (0.62-1.38)	1.15 (0.75-1.76)	0.95 (0.65-1.37)	0.81 (0.48-1.35)
5	0.38 (0.20-0.73)	1.30 (0.78-2.17)	0.64 (0.37-1.11)	1.27 (0.84-1.94)	0.53 (0.30-0.94)

*FPL* Federal Poverty Level, *CI* Confidence intervals

## P15: Exploring the impact of ADHD medication on school absenteeism: a causal analysis of South Carolina Medicaid data

### Zichun Meng^1,2^, Songyuan Deng^1^, Kevin Bennett^1,3^

#### ^1^Center for Rural and Primary Healthcare, Columbia, SC, USA; ^2^Department of Epidemiology and Biostatistics, Arnold School of Public Health, University of South Carolina, Columbia, SC, USA; ^3^Department of Family and Preventive Medicine, School of Medicine Columbia, University of South Carolina, Columbia, SC, USA

##### **Correspondence:** Zichun Meng (zichunm@email.sc.edu)

*BMC Proceedings 2024,* **18(8):**P15

**Study Objective:** Attention-deficit hyperactivity disorder (ADHD) is a prevalent childhood mental health disorder, affecting a significant portion of the pediatric population worldwide. Stimulant medications, commonly prescribed for ADHD, are pivotal in managing symptoms and improving quality of life. Baweja et al. discovered that children who received stimulant treatment experienced reduced rates of school absenteeism and were less prone to be retained [1]. This study aims to build upon previous findings by exploring the nuanced effects of ADHD medication on school absenteeism, particularly focusing on the roles of gender and comorbid conditions, aspects that have not been thoroughly examined in prior research. It’s essential to understand the pivotal role that these medications play in managing ADHD symptoms and their potential influence on educational performance.

**Methods:** This retrospective study analyzed Medicaid claims data from continuously enrolled South Carolina children (2016-2019). We focused on children with at least two ADHD diagnoses, examining the impact of stimulant medications (methylphenidate and amphetamine) on school absenteeism. Gender was considered as a key covariate to explore potential differences in medication effects on absenteeism. Causal forest analysis was employed to estimate the Average Treatment Effect (ATE) and to identify variations in treatment effects across subgroups. In this context, a more negative ATE value is desirable, as it indicates a greater medication effectiveness in reducing absenteeism. This analysis enables a detailed examination of how medication effects may differ across various demographics, including gender.

**Results:** The study focused on 2,881 children aged 7-15 years, diagnosed with ADHD at least twice. Among these, 16% were on methylphenidate and 21% on amphetamine. The Average Treatment Effect (ATE) of ADHD medication on school absenteeism was -0.1569, demonstrating that medication use generally reduced absenteeism. In subgroup analysis, gender-specific differences emerged. For females, the ATE was 0.4947, suggesting that medication was less effective in reducing absenteeism among females compared to males, who had an ATE of -0.2153, indicating a more significant reduction in absenteeism. Furthermore, children with severe asthma showed a substantial decrease in absenteeism with an ATE of -4.7687, demonstrating the effectiveness of ADHD medication in this subgroup.

**Discussion:** The study highlights the nuanced impact of ADHD medication on school absenteeism, varying by gender and comorbid conditions. The findings suggest that medication may be more effective in reducing absenteeism among males and those with severe asthma. These insights emphasize the need for personalized medication strategies and targeted interventions in managing ADHD. Further research should explore the underlying mechanisms of these heterogeneous effects and the role of unobserved confounders.

**Reference**

1. Baweja, R., Mattison, R. E., & Waxmonsky, J. G. (2015). Impact of attention-deficit hyperactivity disorder on school performance: what are the effects of medication?. Pediatric Drugs, 17(6), 459-477. 10.1007/s40272-015-0144-2

## P16: Feasibility of applying big data approaches on diverse institutional research health-related datasets in an open distance higher education institution in South Africa

### Motlatso G. Mlambo, Matseliso P. Molapo, Letlhogonolo M. Marumolwa, Herman J. Visser

#### Department of Institutional Intelligence, University of South Africa, Pretoria, South Africa

##### **Correspondence:** Motlatso G. Mlambo (mlambmg@unisa.ac.za)

*BMC Proceedings 2024,* **18(8):**P16

**Background:** The higher education institution landscape is rapidly evolving; it is complex with increased competitiveness [1] and is facing unprecedented challenges in ensuring the health, well-being and success of its student populations. Therefore, higher education institutions utilize Institutional Research (IR) services for student and staff analyses, aiming to provide evidence-based planning and decision support in teaching, learning, and research. Despite various standalone datasets covering student profiles, teaching experiences, persistence studies, employability, health, climate assessment, and satisfaction, these datasets lack a comprehensive view of attrition and non-persistence learning in distance higher education. The amalgamation of these various datasets presents an opportunity to form a substantial big data repository, offering a consolidated perspective on learning challenges. This, in turn, guides policy and interventions, fostering harmonized data and integrated analyses to enhance student success.

**Aim:** This study explored the feasibility and implications of applying big data methodologies to analyze diverse health-related datasets generated by institutional research in a South African distance higher education institution.

**Methods:** This descriptive desktop study explored an array of institutional research data types and their sources according to calendared, commissioned, and planned research to assess the feasibility of applying big data approaches. Data were sourced from the IR repository, consisting of multiple datasets that informed decision-making processes in the institution. Data were analyzed descriptively by applying Laney’s (2001) big data characteristics of the 5 Vs Model (volume, velocity, veracity, variety and value), which extended from Gartner’s model [2]. In total, 5 staff and 16 student health-related datasets were analyzed (Table 1 and 2).

**Results**

1. Institutional research staff-related health studies
2. Institutional research student-related health studies

**Lessons Learned:** The wealth of health-related data generated through institutional research from student walk and empirical research provides valuable insights worth unravelling in the form of big data. Most non-amalgamated staff and student health-related data did not possess all the five traits of big data; however, the value was high, signifying the relevance of institutional research in higher education. The amalgamated data will require enrichment to apply Big data approaches significantly. Leveraging Big Data technology to gain insights from health IR data sources will be crucial, ultimately contributing to enhancing educational strategies.

**Acknowledgements**

We would like to acknowledge the Specialist Institutional Researchers who led the various studies, producing insightful reports for decision-making purposes. Our appreciation also goes to the analysts in the Business Intelligence Directorate for providing essential datasets enriching the analysis.

*This abstract was selected as the Best Overall Poster Presentation in the Poster Session 2*

**References**

1. Daniel B. Big Data and analytics in higher education: Opportunities and challenges. British journal of educational technology. 2015 Sep;46(5):904-20.
2. Laney D. Data Management: Controlling Data Volume, Velocity, and Variety.–Stamford: META group Inc., 2001. Eng.). Available from: http://blogs.Gartner.com/doug-laney/files/2012/01/ad949-3D-Data-Management-Controlling-Data-Volume-Velocity-and-Variety.

**Table 1 (Abstract P16):** Institutional Research staff related health studies

Focus Area	Dataset		Source	Volume	Velocity	Veracity	Variety	Value
**Health and Well-being**	1	Staff Pulse survey during COVID- 19 (*n*=882)	Survey	Low	Real-time	Low	Structured	High
	2	Staff burnout post Covid 19 (*n*=114)	Survey	Low	Periodic	High	Structured	High
	3	Staff utilisation of health services (5-year trends annual reports)	HR Annual Wellness Reports	Low	Periodic	Low	Unstructured	High
**Satisfaction Levels**	4	Climate research 2012: (*n*=2 027) 2015: (*n*=6 493) 2017: (*n*=3 404) 2021: (*n*=1 226)	Survey	Low	Periodic	High	Structured	High
**Institutional Culture**	5	Workplace bullying (*n*=900)	Survey	Low	Periodic	High	Structured & unstructured	High

**Table 2 (Abstract P16):** Variety of Institutional Research student related health studies

Focus Area	Dataset		Source	Volume	Velocity	Veracity	Variety	Value
**Health and Well being**	1	Student burnout (*n*=5 400)	Survey	Low	Periodic	High	Structured & unstructured	High
	2	Food insecurity among students (*n*=7 464)	Survey	Low	Periodic	High	Structured & unstructured	High
**Satisfaction Levels**	3	Student satisfaction survey: Wave 1- Application & Registration (*n*=88 000). Year: 2005-2023	Survey	Low	Real-time Periodic	High	Structured & unstructured	High
	4	Student satisfaction survey (Wave 2): Teaching & learning and student support (*n*=85 500). Year: 2005-2023	Survey	Low	Periodic	High	Structured & unstructured	High
**Enrolment Trends**	5	International Student Profile	Student Information System	**Medium**	Real-time	Low	Structured	High
	6	Student profile	Student Information System	**High**	Real-time	Low	Structured	High
	7	Application and registration analysis	Student Information System	**High**	Real-time	Low	Structured	High
	8	Application and registration analysis for students with disabilities	Student Information System	Low	Real-time	Low	Structured	High
**Student Engagement Studies**	9	Student Profile Survey (*n*=19 456). Year: 2021 & 2023	Survey		Periodic	High	Structured & unstructured	High
	10	Student Pulse Surveys during Covid-19 (*n*=25 948)	Survey	Low	Real-time	Low	Structured & unstructured	High
	11	Student device and data access (*n*= 224 752)	Survey	Low	Real-time	Low	Structured & unstructured	High
	12	Student Pulse Surveys during Covid-19 (*n*= 25 948)	Survey	Low	Periodic	High	Structured & unstructured	High
	13	Shadowmatch (habits and behaviours) (*n*=80 000)	Survey	Low	Periodic	High	Structured & unstructured	High
	14	Students with disability experiences during Covid 19 (*n*=443)	Survey	Low	Periodic	High	Structured & unstructured	High
	15	First Year Experience study (*n*= 6 853)	Survey	Low	Periodic	High	Structured & unstructured	High
**Employment and career tracer studies**	16	Health Studies Employer survey (*n*=150)	Survey	Low	Periodic	High	Structured & unstructured	High

## P17: Prevention quality indicators and preventable hospital use among adults with traumatic spinal cord injury

### Nicole DiPiro^1^, David Murday^2^, James Krause^1^

#### ^1^College of Health Professions, Medical University of South Carolina, Charleston, SC, USA; ^2^Center for Applied Research and Evaluation, Arnold School of Public Health, University of South Carolina, Columbia, SC, USA

##### **Correspondence:** David Murday (murday@mailbox.sc.edu)

*BMC Proceedings 2024,* **18(8):**P17

**Study Objective:** To assess prevention quality indicators (PQI) developed by the Agency for Healthcare Research and Quality (AHRQ) to identify rates of potentially preventable hospitalizations (ED and inpatient visits) among a population-based cohort of adults with traumatic spinal cord injury (TSCI) in South Carolina (SC).

**Background:** Persons with TSCI are at higher risk for acute and chronic complications, secondary health conditions, and high rates of hospitalization. After TSCI, causes for potentially preventable hospitalization include heart failure, pulmonary edema, pneumonia, urinary tract infections, hypertension, and diabetes [1,2,3,4,5], many of which are avoidable or responsive to high quality primary care [6,7,8]. There is evidence that persons with TSCI receive suboptimal preventive care, but only limited evidence regarding the use of PQI among those with TSCI.

**Methods:** Study procedures were approved by an institutional review board. Participants included all persons in the SC TSCI Surveillance Registry prior to 2015 who were alive and ≥18 years old in 2018 (*n*=2,531). Hospital use during 2016-2018 was tracked by the SC Revenue & Fiscal Affairs Office. Potentially preventable hospital visits were identified using computer code, definitions and framework developed by AHRQ for PQI [9]. National rates for 2018 were calculated by AHRQ; due to smaller numbers, rates for SC and SC residents with TSCI were averaged over 2016-2018.

**Outcome Measures:** PQI are used to identify hospitalizations for acute or chronic ambulatory care sensitive conditions that are potentially preventable with high quality primary/ambulatory care.

**Results:** Hospitalization rates for several conditions for SC adults with chronic TSCI were significantly higher than for those in the general SC population (Table 1). Rates for both SC populations are significantly higher than the national population rates.

**Discussion:** The rates of potentially preventable hospital use for the SC TSCI population are significantly higher for certain conditions than for the SC general population rates. There is a need to better understand these conditions and the primary care needs of patients with TSCI to prevent unnecessary hospitalizations.

**Acknowledgements**

Efforts were supported by a grant from the National Institute on Disability, Independent Living, and Rehabilitation Research (NIDILRR grant number 90IF0119-02-00) and from the SC Spinal Cord Injury Research Fund (SCIRF #2017 SI-02).

**References**

1. Guilcher SJT, Craven BC, et al. Is the emergency department an appropriate substitute for primary care for persons with traumatic spinal cord injury? Spinal Cord. 2013; 51:202-208.
2. Mahmoudi E, Lin P, et al. Preventative services use and risk reduction for potentially preventative hospitalizations among people with traumatic spinal cord injury. Arch Phys Med Rehabil. 2022; 103:1255-62.
3. Khosravi S, Khayyamfar A, et al. Indicators of quality care in individuals with traumatic spinal cord injury: a scoping review. Global Spine J. 2022; 12:166-181.
4. Herrmann AA, Chrenka EA, et al. Potentially preventable readmissions after acute inpatient rehabilitation. Am J Phys Med Rehabil. 2023; 102:1014-1019.
5. Rose S, Stineman M, et al. Potentially avoidable hospitalizations among people at different activity of daily living limitation stages. Health Serv Res. 2017; 52:131-155.
6. Bychkovska O, Tederko P, et al. Does stronger primary care improve access to health services for persons with spinal cord injury? Evidence from eleven European countries. J Spinal Cord Med. 2023; 27:1-11.
7. Lofters A, Chaudhry M, et al. Preventive care among primary care patients living with spinal cord injury. J Spinal Cord Med. 2019; 42:702-708.
8. Milligan J, Lee J, et al. Improving primary care for persons with spinal cord injury: development of a toolkit to guide care. J Spinal Cord Med. 2020; 364-373.
9. Agency for Healthcare Research & Quality. Prevention Quality Indicators (PQI) Benchmark Data Tables, v2021. 2021; http://www.qualityindicators.ahrq.gov.

**Table 1 (Abstract P17):** Comparison of PQI rates (per 100,000) among US and SC populations and the SC population with TSCI

PQI	US	SC	TSCI
	2018	2016-2018	2016-2018
Diabetes Short-Term Complications	82	99	172
Diabetes Long-Term Complications	109	117	172
COPD or Asthma in Older Adults	381	496	917^*^
Hypertension	61	77	120
Heart Failure	430	466	491
Community-Acquired Pneumonia	184	196	504^*^
Urinary Tract Infection	135	154	1,168^*^
Uncontrolled Diabetes	42	52	93
Asthma in Younger Adults	29	30	33
Lower-Extremity Amputation Among Patients with Diabetes	32	39	53
PQI Overall Composite	1,301	1,503	3,303^*^
PQI Acute Composite	318	350	1,672^*^
PQI Chronic Composite	983	1,154	1,631^*^
PQI Diabetes Composite	248	286	477

^*^statistically significant difference (*p*<.05) based on chi square or fisher’s exact test

## P18: Rural-urban and racial differences in cesarean deliveries before and during the COVID-19 pandemic in South Carolina

### Cassie L. Odahowski^1^, Peiyin Hung^1,2,3^, Berry Campbell^4,5^, Jihong Liu^3,6^, Nansi S. Boghossian^6^, Anirban Chatterjee^2^, Yiwen Shih^2^, Chelsea Norregaard^2^, Bo Cai^6^, Xiaoming Li^3,7^

#### ^1^Rural and Minority Health Research Center, Arnold School of Public Health, University of South Carolina, Columbia, SC, USA; ^2^Department of Health Services Policy & Management, Arnold School of Public Health, University of South Carolina, Columbia, SC, USA; ^3^South Carolina SmartState Center for Healthcare Quality, University of South Carolina, Columbia, SC, USA; ^4^Maternal and Fetal Medicine, Obstetrics and Gynecology, PRISMA Health, Columbia, SC USA; ^5^Department of Obstetrics and Gynecology, School of Medicine Columbia, University of South Carolina, Columbia, SC, USA; ^6^Department of Epidemiology and Biostatistics, Arnold School of Public Health, University of South Carolina, Columbia, SC, USA; ^7^Department of Health Promotion, Education, and Behavior, Arnold School of Public Health, University of South Carolina, Columbia, SC, USA

##### **Correspondence:** Cassie L. Odahowski (clo@mailbox.sc.edu)

*BMC Proceedings 2024,* **18(8):**P18

**Objective:** To examine rural and racial/ethnic differences in low-risk cesarean delivery rates before and during the COVID-19 pandemic.

**Methods:** A retrospective cohort of all South Carolina live births delivered from 2018-2021 was identified by linking birth certificate records and all-payer hospital discharge data (*n*=194,393). This study used the data from two low-risk pregnancy cohorts: 1) those with Nulliparous, Term, Singleton, Vertex (NTSV, *n*=65,974) and 2) those without prior cesarean (primary, *n*=167,928). Cesarean outcomes were identified based on birth certificate records and validated using International Classification of Diseases-10^th^ revision (ICD-10), Diagnostic-Related Groups (DRG) and Current Procedural Terminology (CPT) codes. Rural or urban location of birthing hospital was defined using the 2013 Urban Influence Codes. Race and ethnicity of birthing individuals were categorized as non-Hispanic White, non-Hispanic Black, Hispanic, and all other races. Multilevel multivariable logistic regression assessed differences in cesarean outcomes by rural/urban childbirth hospital location and race/ethnicity of birthing people during pre-pandemic (January 2018-February 2020) and peri-pandemic periods (March 2020-December 2021) nested by hospital, adjusting for maternal, infant, and facility characteristics.

**Results:** NTSV and primary cesarean rates differed by rural vs. urban locations of birthing hospital and race/ethnicity but were not exacerbated by the COVID-19 pandemic. A higher proportion of Black birthing people had NTSV and primary cesarean delivery across race/ethnicity groups regardless of hospital location and pandemic period. Adjusted results revealed that Black vs. White disparities remained for NTSV cesarean, but not for primary cesarean. Hispanic individuals had the lowest proportions of NTSV and primary cesarean of all race/ethnicities examined. However, Hispanic individuals had an increased likelihood of NTSV cesarean in rural hospital settings pre-pandemic (aOR=1.28, 95%CI 1.05-1.56), but this disparity was attenuated during the pandemic (aOR=1.13 95%CI 0.93-1.37). The adjusted likelihood of primary cesarean for Hispanic individuals was not different from their White counterparts.

**Discussion:** Linking large-scale health records data allowed us to examine the intersectionality of rural vs. urban birthing location and birthing persons’ race and ethnicity on low-risk cesarean delivery in South Carolina. Our detailed cohort data, representing over 95% of all births in SC, allowed us to identify overlapping rural and racial disparities in low-risk cesarean deliveries. Ongoing surveillance using health records data will be valuable in understanding the temporal trends in disparities and guiding timely development of future interventions to improve equity in South Carolina delivery outcomes.

## P19: Effect of COVID-19 pandemic on medical and preventive healthcare utilization among US children aged 0 to 17 years: evidence from NSCH 2016-2021

### Ibitein Okeafor, Gahssan Mehmood, Syeda Shehirbano Akhtar

#### Department of Health Services Policy and Management, Arnold School of Public Health, University of South Carolina, Columbia, SC, USA

##### **Correspondence:** Ibitein Okeafor (iokeafor@email.sc.edu)

*BMC Proceedings 2024,* **18(8):**P19

**Background:** The multidimensional nature of the Coronavirus-2019 (COVID-19) pandemic, characterized by disruptions in healthcare systems, lockdown measures, and heightened health concerns, has potentially influenced the patterns of medical and preventive healthcare utilization among children [1-3]. Prior research on the effect of the COVID-19 pandemic on healthcare utilization in children employed yearly trend analysis [4-5]. Yearly trends can sometimes mask sudden shifts in data due to gradual changes over time. The pandemic introduced acute disruptions that are not part of a linear trend, thus classifying into two distinct periods (pandemic and pre-pandemic) could more accurately reflect the sudden and profound effects of the pandemic on medical and preventive healthcare utilization.

**Objective:** The study aimed to determine the effect of the COVID-19 pandemic on medical and preventive healthcare utilization among children 0-17 years in the United States.

**Methods:** National Survey of Children’s Health (NSCH) data for 2016-2019 (pre-pandemic period) and 2020-2021 (pandemic period) from the 50 States and Washington DC (*n*= 221,297 representing 71,423,669 children aged 0-17 years) were used to determine the effect of the COVID-19 pandemic (exposure variable) on medical and preventive healthcare utilization (outcome variables). Medical healthcare utilization was defined as any medical care visit from health professionals in the preceding 12 months while preventive healthcare utilization was defined as having one or more preventive healthcare checkups in the preceding 12 months [6]. Multivariable logistic regression analyses were performed to examine the association between exposure and outcome variables after controlling for demographic factors.

**Results:** Medical care visits among children aged 0-17 years were lower in the pandemic period [80.6% (79.9-81.2%)] than pre-pandemic period [83.0% (82.5-83.5%)]. Similarly, preventive care visits showed a decrease in the pandemic period [76.7% (76.0-77.4%)] as compared to the pre-pandemic period [79.8% (79.2-80.3%)] as shown in Table 1. After controlling for demographic variables, the odds of medical care visits [Adjusted Odds Ratio (AOR):0.85;95%CI:0.80-0.90) and preventive care visits [AOR:0.83;95%CI:0.79-0.88] were lower in the pandemic than pre-pandemic periods. Children who were Non-Hispanic Blacks, and Hispanics, resided in rural areas, lived in households with income below 400% Federal Poverty Level, and spoke languages other than English experienced lower odds of having medical and preventive healthcare utilization (Table 2).

**Discussion:** The study reveals lower medical and preventive healthcare utilization among children aged 0-17 years during the COVID-19 pandemic. Despite the need for cautious interpretation, as the 2020 estimates of healthcare utilization in NSCH were based on the preceding 12 months, potentially limiting its sensitivity to the acute effects of the pandemic, the study provides crucial insights on pediatric healthcare utilization. The lower odds of healthcare utilization reported among Non-Hispanic Blacks, Hispanics, those residing in rural areas, households with income below 400% of the Federal Poverty Level, and those with non-English languages signal a critical need for targeted interventions to address and rectify the exacerbated health disparities highlighted by the pandemic. Implementation of public health strategies, policy adjustments, and resource allocation to ensure equitable access to healthcare for all children during crises are hereby advocated.

**Keywords:** Healthcare utilization; preventive care; medical care; children; COVID-19 pandemic

**References**

1. Deolmi M, Pisani F. Psychological and psychiatric impact of COVID-19 pandemic among children and adolescents. Acta Biomed. 2020;91(4):e2020149.
2. Lebrun-Harris LA, Sappenfield OR, Warren MD. Missed and Delayed Preventive Health Care Visits Among US Children Due to the COVID-19 Pandemic. Public Health Rep. 2022 Mar-Apr;137(2):336-343.
3. Patrick SW, Henkhaus LE, Zickafoose JS, Lovell K, Halvorson A, Loch S, Letterie M, Davis MM. Well-being of parents and children during the COVID-19 pandemic: a national survey. Pediatrics. 2020 Oct 1;146(4).
4. Lebrun-Harris LA, Ghandour RM, Kogan MD, Warren MD. Five-year trends in US children’s health and well-being, 2016-2020. JAMA pediatrics. 2022 Jul 1;176(7):e220056.
5. Lyu W, Wehby GL. Changes in Children’s Health Care Access and Utilization in the United States in the First 2 Years of the COVID-19 Pandemic. Academic pediatrics. 2023 Nov 1;23(8):1572-8.
6. US Census Bureau. 2020 National Survey of Children’s Health: methodology report. September 30, 2021. https://www2.census.gov/programs-surveys/nsch/technical-documentation/methodology/2020-NSCH-Methodology-Report.pdf [Accessed Nov 29, 2023].

**Table 1 (Abstract P18):** Summary statistics and proportion of healthcare utilization among children 0-17 years, NSCH 2016-2021

Variables	N (Weighted%)	Medical Care Utilization	Preventive Care Utilization
		Weighted % (95%CI)	Weighted % (95%CI)
**Total**	221297 (100.0%)	82.2 (81.8-82.6)	78.7 (78.3-79.2)
**Pandemic period**			
Pandemic	92680 (33.4)	80.6 (79.9-81.2)	76.7 (76.0-77.4)
Pre-pandemic	128617 (66.6)	83.0 (82.5-83.5)	79.8 (79.2-80.3)
**Age category**			
0 – 3 years	43596 (21.3)	88.7 (87.9-89.5)	86.9 (86.0-87.7)
4 – 7 years	46274 (21.8)	85.1 (84.3-85.9)	82.1 (81.3-83.0)
8 – 11 years	45065 (22.8)	80.0 (79.0-80.8)	75.8 (74.8-76.7)
12 – 14 years	38768 (17.2)	78.7 (77.6-79.8)	74.7 (73.6-75.8)
15 – 17 years	47594 (17.0)	76.9 (75.9-77.8)	72.3 (71.2-73.3)
**Sex**			
Female	106767 (48.9)	82.3 (81.7-82.8)	78.8 (78.1-79.4)
Male	114530 (51.1)	82.2 (81.6-82.7)	78.7 (78.2-79.3)
**Educational attainment**			
Less than high school	5343 (9.1)	61.7 (59.2-64.1)	57.6 (55.1-60.0)
High school degree/GED	28350 (19.2)	75.2 (74.1-76.3)	71.1 (70.0-72.2)
Some college/technical school	49867 (21.5)	81.8 (81.0-82.5)	77.9 (77.0-78.7)
College degree or higher	137737 (50.2)	88.8 (88.4-89.2)	85.9 (85.5-86.3)
**Race-ethnicity**			
NH White	151183 (51.3)	86.5 (86.2-86.9)	82.9 (82.5-83.3)
NH Black	13897 (13.2)	80.0 (78.8-81.2)	77.2 (75.9-78.4)
Hispanic	26978 (24.9)	75.5 (74.2-76.7)	72.1 (70.7-73.3)
Others	29239 (10.9)	80.1 (79.0-81.1)	76.6 (75.5-77.7)
**Family income**			
0 – 99% FPL	25435 (19.3)	72.7 (71.5-73.9)	69.1 (67.8-70.3)
100 – 199% FPL	35552 (21.4)	78.6 (77.5-79.6)	74.7 (73.6-75.8)
200 – 399% FPL	68123 (28.1)	83.6 (82.9-84.2)	79.8 (79.0-80.5)
400% FPL or greater	92187 (31.2)	89.3 (88.9-89.7)	86.6 (86.1-87.0)
**Language spoken at home**			
English	205847 (85.4)	84.5 (84.1-84.9)	81.0 (80.6-81.4)
Spanish/other	15450 (14.6)	68.9 (67.3-70.6)	65.4 (63.7-67.1)
**Rurality**			
Urban	135537 (77.5)	82.3 (81.8-82.8)	79.0 (78.5-79.5)
Rural	27888 (10.4)	79.1 (78.1-80.0)	73.9 (72.9-74.9)
Unspecified	57872 (12.0)	84.1 (83.5-84.7)	81.1 (80.5-81.8)
**Region**			
West	59931 (24.2)	79.4 (78.3-80.4)	75.5 (74.4-76.6)
Northeast	37756 (15.9)	86.2 (85.4-87.0)	84.0 (83.1-84.8)
Midwest	54021 (21.1)	82.9 (82.3-83.5)	79.4 (78.8-80.1)
South	69589 (38.8)	81.9 (81.3-82.6)	78.3 (77.6-79.0)
**Out of pocket care expenses**			
None or <$250	114691 (60.5)	78.8 (78.2-79.4)	75.5 (74.9-76.1)
$250-$499	37325 (14.2)	87.2 (86.4-88.0)	83.1 (82.2-84.0)
$500-$999	28345 (10.3)	88.1 (87.1-89.0)	84.4 (83.4-85.5)
$1000-$5000	38488 (13.6)	89.0 (88.2-89.8)	85.7 (84.8-86.5)
Undisclosed	2448 (1.4)	69.5 (65.3-73.4)	65.0 (60.7-69.0)
**Child overall health status**			
Excellent/good	218675 (98.4)	82.2 (81.8-82.6)	78.8 (78.3-79.2)
Fair/poor	2622 (1.6)	84.2 (81.8-82.6)	77.5 (72.3-81.9)

*FPL* Federal Poverty Level, *NH* Non-Hispanic GED

**Table 2 (Asbtract P18):** Multivariable logistic regression model with medical and preventive healthcare utilization depression among children aged 0-17years, NSCH 2016-2021

Variables^**a**^	Medical Care Utilization		Preventive Care Utilization	
	Crude OR (95%CI)	Adjusted OR (95%CI)	Crude OR (95%CI)	Adjusted OR (95%CI)
**Pandemic period**				
Pandemic	0.847 (0.802-0.896)	0.845 (0.797-0.895)	0.836 (0.794-0.879)	0.833 (0.789-0.878)
Pre-pandemic	1	1	1	1
**Age category**				
0 – 3 years	1	1	1	1
4 – 7 years	0.728 (0.656-0.808)	0.717 (0.645-0.797)	0.693 (0.630-0.763)	0.682 (0.619-0.752)
8 – 11 years	0.507 (0.459-0.560)	0.506 (0.458-0.560)	0.471 (0.429-0.516)	0.469 (0.427-0.515)
12 – 14 years	0.470 (0.424-0.522)	0.465 (0.418-0.516)	0.446 (0.405-0.490)	0.441 (0.401-0.486)
15 – 17 years	0.422 (0.382-0.466)	0.409 (0.369-0.453)	0.392 (0.358-0.429)	0.382 (0.348-0.419)
**Sex**				
Female	1	1	1	1
Male	0.992 (0.939-1.050)	0.996 (0.939-1.056)	0.998 (0.948-1.051)	1.001 (0.948-1.055)
**Educational attainment**				
Less than high school	0.202 (0.181-0.226)	0.371 (0.327-0.425)	0.222 (0.1998-0.248)	0.386 (0.339-0.439)
High school degree/GED	0.382 (0.357-0.409)	0.545 (0.502-0.592)	0.404 (0.379-0.431)	0.556 (0.515-0.600)
Some college/technical school	0.564 (0.528-0.602)	0.706 (0.656-0757)	0.577 (0.544-0.612)	0.710 (0664-0.759)
College degree or higher	1	1	1	1
**Race-ethnicity**				
NH White	1	1	1	1
NH Black	0.625 (0.576-0.677)	0.847 (0.777-0.924)	0.699 (0.648-0.753)	0.931 (0.858-1.010)
Hispanic	0.479 (0.445-0.515)	0.858 (0.787-0.936)	0.533 (0.498-0.571)	0.924 (0.852-1.001)
Others	0.625 (0.582-0.672)	0.701 (0.649-0.757)	0.678 (0.634-0.725)	0.746 (0.695-0.801)
**Income based on FPL**				
0 – 99% FPL	0.319 (0.295-0.345)	0.679 (0.616-0.749)	0.347 (0.322-0.373)	0.679 (0.619-0.744)
100 – 199% FPL	0.439 (0.407-0.475)	0.814 (0.746-0.889)	0.459 (0.428-0.493)	0.793 (0.732-0.859)
200 – 399% FPL	0.609 (0.569-0.652)	0.797 (0.745-0.855)	0.611 (0.575-0.649)	0.779 (0.731-0.829)
400% FPL or greater	1	1	1	1
**Language spoken at home**				
English	1	1	1	1
Spanish/other	0.408 (0.375-0.443)	0.682 (0.613-0.757)	0.443 (0.409-0.479)	0.699 (0.633-0.772)
**Rurality**				
Urban	1	1	1	1
Rural	0.810 (0.758-0.866)	0.826 (0.769-0.886)	0.751 (0.706-0.799)	0.779 (0.731-0.832)
Unspecified	1.135 (1.071-1.202)	1.008 (0.944-1.076)	1.142 (1.083-1.204)	1.029 (0.969-1.092)
**Region**				
West	1	1	1	1
Northeast	1.623 (1.477-1.783)	1.494 (1.355-1.649)	1.698 (1.556-1.854)	1.592 (1.454-1.743)
Midwest	1.258 (1.164-1.359)	1.101 (1.011-1.199)	1.253 (1.166-1.346)	1.150 (1.064-1.243)
South	1.178 (1.089-1.274)	1.166 (1.069-1.273)	1.169 (1.087-1.257)	1.176 (1.085-1.274)
**Out of pocket care expenses**				
None or <$250	1	1	1	1
$250-$499	1.833 (1.689-1.988)	1.371 (1.259-1.492)	1.600 (1.487-1.722)	1.225 (1.135-1.323)
$500-$999	1.993 (1.809-2.197)	1.508 (1.361-1.670)	1.761 (1.616-1.918)	1.366 (1.249-1.494)
$1000-$5000	2.177 (1.991-2.379)	1.563 (1.426-1.714)	1.944 (1.799-2.100)	1.437 (1.325-1.558)
Undisclosed	0.614 (0.506-0.745)	0.668 (0.541-0.824)	0.601 (0.499-0.725)	0.649 (0.530-0.794)
**Child overall health status**				
Excellent/good	1	1	1	1
Fair/poor	1.153 (0.833-1.594)	1.929 (1.391-2.675)	0.927 (0.702-1.223)	1.485 (1.134-1.944)

*FPL* Federal Poverty Level, *NH* Non-Hispanic, *CI* Confidence intervals

^a^Outputs are in 3 decimal places to highlight differences in values

## P20: Leveraging big heterogenous HIV-related data in the era of Protection of Personal Data Act in Sub Saharan Africa: lessons learned from the Boloka project

### Refilwe Nancy Phaswana-Mafuya^1,2^, Edith Phalane^1,2^, Amrita Rao^3^, Kalai Willis^3^, Kate Rucinski^3^, Karen Alida Voet^3^, Amal Abdulrahman^3^, Claris Siyamayambo^1,2^, Betty Sebati^1,2^, Mohlago Seloka^1,2^, Musa Jaiteh^1,2^, Lucia Olifant^1,2^, Katherine Journeay^3^, Haley Sisel^3^, Francois Wolmarans^4^, Xiaoming Li^5^, Bankole Olatosi^5^, Lifutso Motsieloa^6^, Mashudu Rampilo^6^, Stefan David Baral^3^

#### ^1^South African Medical Research Council/University of Johannesburg (SAMRC/UJ) - Pan African Centre for Epidemics Research (PACER) Extramural Unit, Johannesburg, South Africa; ^2^Department of Environmental Health, University of Johannesburg, Johannesburg, South Africa; ^3^Key Populations Program, Center for Public Health and Human Rights, Johns Hopkins Bloomberg School of Public Health, Baltimore, MD, USA; ^4^University of Johannesburg Technology Architecture & Planning, University of Johannesburg, Johannesburg, South Africa; ^5^Department of Health Services Policy and Management, Arnold School of Public Health, University of South Carolina, Columbia, SC, USA; ^6^South African National AIDS Council, Pretoria, South Africa

##### **Correspondence:** Edith Phalane (edithp@uj.ac.za)

*BMC Proceedings 2024,* **18(8):**P20

**Issues:** Big heterogenous HIV-related data has many benefits including data optimization and advancing scientific discoveries as well as ensuring targeted approaches in the HIV response. The Protection of Personal Data Act (POPIA) no.4 of 2013 in South Africa presents unique opportunities in terms of adherence to data sharing standards and data security. However, complexities around confidentiality, privacy, anonymity, and accessibility of data persist. Against this background we share lessons learned so far from The Boloka Project in leveraging big heterogeneous Key Populations (KPs) and HIV-related data in the era of POPIA.

**Project:** The Harnessing big heterogeneous data to evaluate the potential impact of HIV responses among key populations in Sub Saharan Africa Project (henceforth referred to as the Boloka Project) seeks to bring together data of different types across places, periods and populations among KPs for harmonization, synthesis, linkages, and analysis to inform a targeted HIV response in a region that is hardest hit by HIV epidemic. The Boloka Project has five stages (see Figure 1). This paper focuses on Stages 1 and 2 which has to do with the establishment of meaningful data partnerships to secure HIV-related data to answer new research questions that would inform the HIV response. The process of establishing data partnerships involved development of HIV-related indicator list and data catalogue based on the UNAIDS Data Monitoring tool and National Strategic Plan HIV, STI and TB 2023-2028. This was followed by approaching and securing data partners, signing of data sharing and data processing agreements. The data sharing agreement were aligned with the “protection of personal information”, henceforth referred to as the POPI Act 4 of 2014, and any other applicable data protection regulation and institutional legal requirements. The data partnership agreement outlined the obligations, expectations, and roles of both parties, type(s) of data, as well as utilization and expected outcomes from the shared data.

**Lessons Learned:** Adherence and alignment to the institutional alignment. This can make the process of data sharing quite lengthy and tedious, affecting the project progress. Best practices in terms of data sharing are needed to enable advance in scientific inquiry. Training on POPIA and institutional mechanisms to support POPIA regulations are critical. Understanding the data processes, and procedures as set out in POPIA is key. Involving multiple role players including legal experts for ongoing advice and support is necessary. Having Standard Operating Procedures in place is helpful in guiding the implementation of POPIA. Overall, adherence to best practices in data sharing will enhance data optimization and advancement of scientific discoveries.

**Fig. 1 (Abstract P20):**
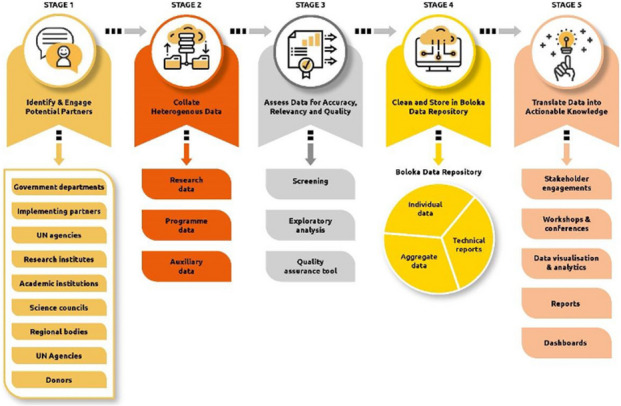
Five stages of the Boloka data repository

## P21: Identifying genetic contributions to adverse drug reactions using big data

### Scott Reed

#### Department of Chemistry, College of Liberal Arts and Sciences, University of Colorado Denver, Denver, CO, USA

##### **Correspondence:** Scott Reed (scott.reed@ucdenver.edu)

*BMC Proceedings 2024,* **18(8):**P21

**Background:** Idiosyncratic adverse drug reactions (ADR) are rare events that nevertheless limit the benefits of drugs and in some cases lead to withdrawal of drugs. Some genetic causes for ADRs are known and rare genetic variants could explain why other ADRs occur after drugs are approved which is not surprising since clinical trials do not have sufficient study size to power the observations of ADRs caused by rare genetic variants. We are using machine learning and computational chemistry to predict what genetic signatures could explain some ADRs.

**Methods:** Considering the number of possible drug and drug metabolite-protein combinations that are possible, it essential to have fast tools and a sorting method to select those likely to yield results. Our tools are a first step toward rapidly identifying candidate ADR-SNV interactions. We are building web applications that facilitate this search.

**Results:** We have created a web application (https://pharmacogenomics.clas.ucdenver.edu/pharmacogenomics/side-effect/) [1] that collects and provides information on pharmaceuticals, their side effects, and the predicted metabolites of those drugs. The application, called Metabolovigilance, is a fast easy way for researchers to access information and chemical structures.

Another web application, GTExome (https://pharmacogenomics.clas.ucdenver.edu/gtexome/), provides on-the-fly creation of protein structures with known mutations to explore as possible sources of ADRs [2]. This tool is built from two primary data sources; the Exome Aggregation Consortium (ExAC)/Gnomad data available from the Broad Institute [3] and the NIH Common fund GTEx database [4]. Drawing from these two large datasets, GTExome provides tissue specific information on the distribution of genetics variants. Using GTExome a list of GeneIDs is produced and from the GeneIDs a list of SNVs is prepared. The ExAC and Gnomad datasets provide information on all known SNVs within the human exome. The tool allows for sorting by frequency, ancestry specific frequency, and other characteristics. By using GTEx as input data for performing our SNV searches we avoid non-coding regions of the genome and filters for missense only. Our web application currently retrieves SNVs based on the proteins listed in our GTEx results and then produces 3D structure files for a mutated protein using FASPR based on either AlphaFold2 structure [5] or an experimental structure (when available) as selected by user after metrics are provided.

**Discussion:** Our open-access, fast screening tools provide suitably formatted three-dimensional structures of drugs and their metabolites combined with protein structures while simplifying the process of performing docking and analyzing the results. Together, the output of these tools can be used for *in silico* analysis of drug interactions as a prelude to experimental validation.

We are seeking to use machine learning techniques to identify false positives in virtual screens for binding of drugs and metabolites to SNVs. The use of high-quality decoys in particular, is likely to help remove false positives from the results. We are seeking to create new bioinformatics tools for identifying the off-target binding sites of pharmaceuticals and their metabolites. Our goal is to build tools that help identify the underlying genetic causes of rare and unexplained ADRs so these effects can be avoided. Together these tools can be used by researchers to prioritize drug protein interactions for further *in silico* or *in vitro* studies.

**References**

1. Tan H, Reed S. Metabolovigilance: Associating Drug Metabolites with Adverse Drug Reactions. *Molecular Informatics.* 2022; *41,* 2100261.
2. Jill Hoffman, Henry Tan, Clara Sandoval-Cooper, Kaelyn de Villiers, Scott M. Reed GTExome: Modeling commonly expressed missense mutations in the human genome. bioRxiv 2023.11.14.567143; doi: 10.1101/2023.11.14.567143
3. Lek M., *et al.* Analysis of protein-coding genetic variation in 60,706 humans *Nature* 2016, *536*, 285–291.
4. Gamazon, E.R., Segrè, A.V., van de Bunt, M. *et al.* Using an atlas of gene regulation across 44 human tissues to inform complex disease- and trait-associated variation. *Nat Genet* 50, 956–967 (2018).
5. Jumper, J. *et al.* Highly accurate protein structure prediction with AlphaFold. *Nature* (2021). 10.1038/s41586-021-03819-2.

## P22: Scoring of pediatric respiratory syncytial virus (RSV) infection severity: a systematic review

### Zoe E. Sanders, Kenny Nguyen, Jacob Estrada, Debbie Barrington, Jennifer Grier

#### School of Medicine Greenville, University of South Carolina, Greenville, SC, USA

##### **Correspondence:** Zoe E. Sanders (sandersz@email.sc.edu)

*BMC Proceedings 2024,* **18(8):**P22

**Study Objectives:** Respiratory syncytial virus (RSV) is the leading cause of acute bronchiolitis in children worldwide and many factors may contribute to infection severity. Several clinical scoring tools have been developed to evaluate the severity of bronchiolitis infections and aid in treatment decisions. However, these tools often lack validation, exhibit parameter variations, and are not designed to specifically address RSV infections. This study aims to systematically review RSV severity scoring systems applied to pediatric patients (<18 years old) in order to establish a comprehensive understanding of RSV infection severity classification for use in the evaluation of patient data sets.

**Methods:** A systematic review of studies describing the development or use of severity scores (≥2 variables) was conducted using PubMed. Search terms included *severity, severe, severity of illness index, classification, RSV, respiratory syncytial virus, respiratory syncytial infection,* and *respiratory syncytial viruses*. Reviews, systematic reviews, meta-analyses, commentaries, letters, and cohort studies with populations aged >18 years old were excluded. Remaining studies were then screened for relevance and the presence of a scoring system. Screening was performed by two independent researchers. Parallel searches are currently being conducted in the Embase, Web of Science, and CINAHL Ultimate databases to reflect a broader range of data.

**Results:** From PubMed, a total of 4884 articles were identified, 334 met initial inclusion criteria and were screened for use of scoring systems; a total of 66 were included in the final analysis. Among the 66 studies included, 28% (19/66) developed independent scores, while 71% (47/66) used or modified four frequently encountered tools. Notably, all tools incorporated clinical and physical exam findings into their scoring criteria. However, results indicate substantial variation in these parameters.

**Discussion:** Our findings demonstrate the extensive variability in classifying RSV infection severity and the need for a standardized comprehensive scoring method. These results will guide the development of an evidence-based severity scoring tool to be applied in the evaluation of pediatric patients in Upstate, South Carolina. RSV infection severity scores will be paired with analysis of patient demographics or other health-associated variables to inform the local community about their RSV risk status. Understanding RSV infection severity and risks in South Carolina children will drive targeted educational efforts, enhance clinical decision-making, and improve patient outcomes.

*This abstract was selected as the Best Poster Presentation from an Emerging Scholar in the Poster Session 1*

## P23: Optimizing big data management in healthcare databases: experimental approaches for efficient data engineering

### Ehsan Soltanmohammadi, Neset Hikmet

#### Department of Integrated Information Technology, College of Engineering and Computing, University of South Carolina, Columbia, SC, USA

##### **Correspondence:** Ehsan Soltanmohammadi (ehsans@email.sc.edu)

*BMC Proceedings 2024,* **18(8):**P23

**Background:** In the healthcare sector, a pronounced technical proficiency gap poses a challenge, impeding the efficient utilization of extensive datasets for meaningful analysis and decision-making. Despite possessing domain expertise, healthcare professionals often lack the necessary technical skills in advanced big data engineering, hindering resource optimization and innovative approaches for disease diagnosis, patient monitoring, and remote healthcare. Addressing this challenge involves solving the issue of big data management in healthcare, aiming to bridge the proficiency gap and enhance the utilization of data for improved healthcare outcomes.

**Methods of Research:** This presentation outlines our research on optimizing big data management in healthcare databases, focusing on experimental approaches.

1. Experimentation on Different Relational Database Engines:
  Purpose: Assess the efficacy of relational database engines (e.g., MySQL, PostgreSQL, Microsoft SQL Server) in handling healthcare data.
  Approach: Conducted experiments using diverse database engines to identify the most suitable one for different bigdata healthcare use cases.
  First Step: Selection of representative healthcare datasets and experimental design.
  Significance for Presentation: Illustrate the importance of the initial steps in selecting databases and designing experiments.
2. Customized Methods of Data Management:
  Purpose: Implement and evaluate tailored data management methods for efficient healthcare data engineering within relational databases.
  Approach: Developed methods include optimizing data indexing, partitioning, normalization strategies, and query optimization.
  First Step: Design and implementation of customized data management methods.
  Significance for Presentation: Emphasize the critical role of method design and implementation in achieving efficient healthcare data management.

**Results and Findings:** Key findings include the development of experimental data engineering methods enhancing data quality, computational efficiency, and scalability in diverse healthcare use cases. Tailored approaches for different healthcare scenarios underscore the need for efficient big data storage and pipelines. Practical experimentation, comparing data engineering pipelines in containerized applications against traditional pipelines, highlights tangible benefits in real-world scenarios, demonstrating practical applicability in modern healthcare data management.

**Lessons Learned:**

1. Focus on Efficiency in Different Scenarios:
  Customized data management methods exhibit varying efficiencies in different healthcare scenarios.
  Tailoring approaches to specific use cases enhances the overall efficiency of big data management in healthcare databases.
2. Interdisciplinary Collaboration:
  Collaboration between healthcare professionals and data experts is crucial for effective data management and analysis in big data engineering.
3. Ethical Considerations:
  Responsible handling of patient data is emphasized, maintaining trust in healthcare systems in the evolving landscape of data-driven healthcare.

## P24: Covid-19 pandemic associate with opioid overdose

### Wenjie Sun

#### Center for Health Sciences, Oklahoma State University, Stillwater, OK, USA

##### **Correspondence:** Wenjie Sun wenjie.sun@okstate.edu

*BMC Proceedings 2024,* **18(8):**P24

**Introduction/Objectives:** Covid-19, which has been a global pandemic for 3 years, as exposure to fatal death, is associated with an increase in the development of both stress and mental health events. The current opioid epidemic has often been portrayed as a white problem. Opioid overdose deaths were overwhelmingly dominated by non-Hispanic white people. As the opioid crisis continues to evolve, several studies have called for attention to the spread of opioid overdoses among nonwhite populations. To explore the relationship between social isolation and opioid overdose, and age, gender, and race/ethnic variation on the relationship of social isolation and opioid overdose.

**Methods:** The opioid over-dose related emergency room (ER) admission records with ICD-10 code will be pulled from the EHR (The EHR database (ORACLE Cerner©). Time series analytics method was applied to analysis to data from three time periods which were categorized into three stages by control measurements during the pandemic from 2019-2022. The study measures opioid overdose using emergency department (ED) visit data. Visits are classified as heroin poisoning, non-heroin poisoning, and opioid use disorder (OUD) ED visits. Measures of opioid overdose including emergency admission (including daily count of opioid overdose in emergency admission, death of opioid overdose, and percentage of opioid overdose of emergency admission).

**Results:** The rates of ER visits related to opioid use significantly increased during the pandemic after Covid-19 measures were implemented, compared to the period before the pandemic (*P*<0.05). Opioid-related causes accounted for approximately 1 in 100 ER visits. The percentage of ER admissions due to opioid overdose also increased during the three stages of the pandemic, according to time-series analysis (*P*<0.05). Furthermore, health disparities across race/ethnicity were observed in ER admissions due to opioid overdose, with minorities being significantly higher compared to others.

**Conclusions:** Long time social distance for prevent the virus spread including shut down school and is a kind of isolation. It contributes to the opioid overdose which was the most important public health issue across the states before pandemic.

**Keywords:** COVID-19; Opioid; time series

## P25: Physical activity, sedentary behavior and BMI among adolescents: the mediate role of weight concern regulated by body image perception

### Shuoyuan Tan^1^, Libo Zhou^2^, Gulqihra Abdukerima^1^, Wei Yin^3^, Pauline Sung-Chan^4^, Ning Chen^1^, Ling Yuan^1^, Ya Gao^1^, Zhaoxin Wang^5,6^, Jianwei Shi^7,8^

#### ^1^School of Public Health, Shanghai Jiaotong University School of Medicine, Shanghai, China; ^2^Hainan Province Anning Hospital, Haikou, China; ^3^Department of Social and Behavioral Sciences, College of Liberal Arts and Social Sciences, City University of Hong Kong, Hong Kong, China; ^4^Hong Kong Institute of Economics & Business Strategy, HKU School of Business, The University of Hong Kong, Hong Kong, China; ^5^Department of Dermatology, The Fifth People’s Hospital of Hainan Province, Hainan Medical University, Haikou, China; ^6^School of Management, Hainan Medical University, Haikou, China; ^7^Department of General Practice, Yangpu Hospital, Tongji University School of Medicine, Shanghai, China; ^8^Department of Social Medicine and Health Management, School of Public Health, Shanghai Jiaotong University School of Medicine, Shanghai, China

##### **Correspondence:** Jianwei Shi (shijianwei_amy@126.com)

*BMC Proceedings 2024,* **18(8):**P25

**Objects:** Obesity significantly elevates the risk of non-communicable diseases and contributes to heightened feelings of body dissatisfaction. China, in particular faces a notable challenge with high obesity rates. Physical activity is widely acknowledged as a potent intervention in addressing adolescent obesity and sedentary behavior are recognized as integral components of physical inactivity. While it is widely acknowledged that physical activity is associated with Body Mass Index (BMI) status, the psychological factors underlying this relationship remain poorly understood. This study aimed to develop a moderated mediation model to investigate whether weight concern mediates this association, conditional on body image perception.

**Methods:** In 2015, this survey initially recruited 937 adolescents in grades 6 to 8, and after one year, a subset of 508 participants (54.216%) was followed up. In 2016, These 508 participants completed a battery of questionnaires assessing their physical activity level, sedentary behavior, weight concern and body image perception. Path analysis was conducted to examine the moderated mediation model, and the indirect effects were evaluated using the bootstrap procedures with bias‑corrected 95% confidence intervals.

**Results:** Our findings revealed that weight concern played a mediating role in the link between sedentary behavior and BMI (*β* =0.044, *P* < 0.001), which was moderated by body images perception (Figure 1). Specifically, the conditional indirect effect between sedentary behavior and BMI was significant (Table 1), regardless of whether body image perception was accurate (effect = 0.050, 95% CI= [0.003, 0.105]) or inaccurate (effect = 0.142, 95% CI = [0.008, 0.288]).

**Conclusions:** Weight concern operates as a mediator in the link between sedentary behavior and BMI, indicating that sedentary behavior exerts an indirect influence on BMI through weight concern. Body image perception moderates this mediated pathway through weight concern, with the indirect effect being notably more pronounced among adolescents who hold an inaccurate perception of their body image.

**Discussion:** This study is the first to employ structural equation modeling to elucidate the complex interplay between physical activity levels and BMI from a psychological perspective, with the intention of developing more suitable weight management programs for Chinese adolescents. In addition, the inclusion of sedentary behaviors contributes to a more holistic profile of the physical activity habits of adolescents. Our findings hold practical implications, particularly in the treatment of obese adolescents, reducing sedentary behaviors and fostering a healthy body image perception, alongside decreasing the frequency of self-weighing, are advisable strategies to mitigate BMI.

**Fig. 1 (Abstract P25):**
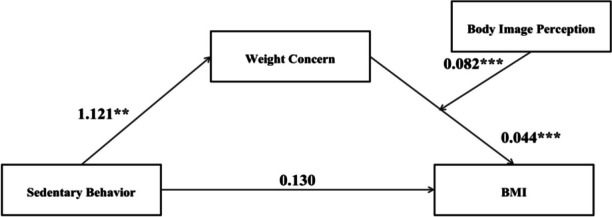
Path coefficients of the moderated mediation model. * Indicates significant paths: *p < 0.05, **p < 0.01, ***p < 0.001

**Table 1 (Abstract P25):** Conditional indirect effect of weight concern mediated the association between sedentary behavior and BMI moderated by body image perception. (Bias-corrected percentile bootstrap analysis)

Mediator	Body image perception	Effect	BootSE	BootLLCI	BootULCI
Weight concern	0	0.050	0.026	0.003	0.105
	1	0.142	0.071	0.008	0.288

## P26: Neighborhood disinvestment and racial/ethnic disparities in peripartum cardiomyopathy in California, from 2004-2019

### Curisa Tucker^1^, Chen Ma^2^, Mahasin Mujahid^3^, Afshan Hameed^4^, Audrey Lyndon^5^, Elliot Main^2^, Suzan Carmichael^2^

#### ^1^College of Nursing, University of South Carolina, Columbia, SC, USA; ^2^School of Medicine, Stanford University, Palo Alto, CA, USA; ^3^School of Public Health at the University of California, Berkeley, CA, USA; ^4^School of Medicine, University of California, Irvine, CA, USA; ^5^Rory Myers College of Nursing, New York University, NY, USA

##### **Correspondence:** Curisa Tucker (tuckercm@mailbox.sc.edu)

*BMC Proceedings 2024,* **18(8):**P25

**Introduction:** Peripartum cardiomyopathy (PPCM) is a rare life-threatening medical condition presenting as idiopathic heart failure in late pregnancy or during the early postpartum period in otherwise healthy patients. Racial disparities in the rates of morbidity and mortality of PPCM have been extensively documented where higher rates occur in Black patients. There is minimal research examining the relationship between racial/ethnic disparities in PPCM and social determinants of health such as neighborhood social disadvantage.

**Hypothesis:** We hypothesize that neighborhood social disadvantage is associated with PPCM and it contributes to racial/ethnic disparities in PPCM, pre-pregnancy hypertension contributes to disparities, and that the timing of PPCM mostly occurs during the postpartum period.

**Methods:** We used data obtained from the California Department of Health Care Access and Information, which included vital records longitudinally linked with hospital discharge records for mothers and infants up to 9 months postpartum for births in California from 1997-2018. We created and standardized the Neighborhood Deprivation Index (NDI) as a proxy measure for neighborhood social disadvantage and categorized it into quartiles, with quartile 1 indicating the least deprivation and quartile 4 indicating the most deprivation. We examined the distribution of population characteristics for non-Hispanic White, non-Hispanic Black, non-Hispanic Asian and Hispanic births in the sample by PPCM status. We sequentially adjusted multivariable logistic regression models by maternal and clinical characteristics to estimate the association of NDI and race/ethnicity with PPCM using non-Hispanic White as the reference group. We report odds ratios (OR) and 95% confidence intervals (CI) that reflect the total change in odds of PPCM, and calculated the timing of PPCM diagnosis.

**Results:** Our study included 6,970,681 births and 862 (0.012%) PPCM cases. After adjustment, in the NDI regression models, quartile 2 (Q2) through quartile 4 (Q4) had elevated risk for PPCM (Q2: OR 1.2 (CI 1.0-1.5); Q3: OR 1.7 (CI 1.4-2.1); Q4: OR 1.5 (CI 1.2-1.9)). When adjusting for NDI in the race/ethnicity models, the odds of PPCM slightly decreased for each race/ethnicity. After adjusting for pre-pregnancy hypertension in both the NDI and race/ethnicity models, the odds of PPCM slightly decreased for quartile 3 and 4 and in NH Black births. Most PPCM cases (60.4%) were identified during a postpartum hospital encounter.

**Conclusions:** Our results show that neighborhood deprivation and pre-pregnancy hypertension partially explain the racial/ethnic disparities in PPCM. Future research should examine the impact of specific measures of neighborhood deprivation (e.g., access to care, employment levels etc.) on the racial/ethnic disparity in outcomes such as PPCM.

## P27: Leveraging big data in affective neuroscience

### Xuan Yang^1,2^, Christian O’Reilly^2,3,4^, Svetlana V. Shinkareva^1,2^

#### ^1^Department of Psychology, College of Arts and Sciences, University of South Carolina, Columbia, SC, USA; ^2^Institute for Mind and Brain, College of Arts and Sciences, University of South Carolina, Columbia, SC, USA; ^3^Department of Computer Science and Engineering, College of Engineering and Computing, University of South Carolina, Columbia, SC, USA; ^4^Artificial Intelligence Institute, College of Engineering and Computing, University of South Carolina, Columbia, SC, USA

##### **Correspondence:** Svetlana V. Shinkareva (shinkareva@sc.edu)

*BMC Proceedings 2024,* **18(8):**P27

**Background:** Affective processing plays a crucial role in every aspect of human psychological functioning, including perception, memory, judgment, and mental health. A better understanding of its neural mechanisms would provide a conceptual framework for current psychopathological therapies and inspire new applications [1]. Conventional functional magnetic resonance imaging (fMRI) studies use small sample sizes (*N*<30), often yielding inconsistent results. Moreover, the existing research often relies on controlled laboratory stimuli and has left a gap in our understanding of affective processing in real-life contexts. Listening to narratives in the scanner resembles some daily activities and is well-suited to study affective processing in an ecologically-valid way. However, behavioral methods for obtaining affective ratings of narratives are time-consuming and costly. To address these limitations, the current study harnesses the power of big data by leveraging a large fMRI database and the lexical-level affective ratings from a psycholinguistic metabase to localize the affective neural network during naturalistic narrative listening.

**Materials and Methods:** We combined seven datasets from the Narrative fMRI data collection [2], for a total of 213 fMRI scans of healthy young adults listening to one of ten narratives (8-55 min). The lexical-level affective ratings from the South Carolina Psycholinguistic Metabase (SCOPE) [3] were used as an alternative to traditional subjective assessments. We used statistical parametric modeling to identify brain regions correlating with these affective ratings. The reliability of this method was evaluated on a smaller subset of data (128 scans) associated with four narratives for which we have collected phrase-level affective ratings in independent behavioral experiments (*N* = 157).

**Results:** Brain regions sensitive to valence according to both the phrase-level and lexical-level ratings were consistent with the core affect neural network [4]. Brain regions identified using phrase-level ratings included the amygdala/hippocampus, medial prefrontal cortex (mPFC), superior temporal gyrus (STG), anterior temporal lobe (ATL), and precuneus. Brain regions identified using the lexical-level ratings included mPFC, STG, ATL, and precuneus, which suggested that the lexical-level ratings from large corpora provide a reliable measure of the human perceived affect during naturalistic narrative listening.

**Conclusion:** Our findings support the feasibility and reliability of replacing human behavioral ratings with lexical ratings from large corpora in naturalistic contexts. This approach could greatly enhance efficiency in any fields needing affective assessment and support high-throughput, large-scale brain decoding from wearable sensors (e.g., EEG) during daily, out-of-the-lab exposure to speech (e.g., through automatic transcription followed by lexical ratings).

**References**

1. Harrison, N. A., & Critchley, H. D. The British Journal of Psychiatry. Affective neuroscience and psychiatry. 2017; 191(3), 192-194.
2. Nastase, S.A., Liu, YF., Hillman, H. et al. Scientific Data. The “Narratives” fMRI dataset for evaluating models of naturalistic language comprehension. 2021; 8:250.
3. Gao, C., Shinkareva, S.V. & Desai, R.H. Behavior Research Methods. SCOPE: The South Carolina psycholinguistic metabase. 2023; 55: 2853–2884.
4. Lindquist KA, Wager TD, Kober H, Bliss-Moreau E, Barrett LF. Behavioral and Brain Sciences. The brain basis of emotion: a meta-analytic review. 2012; 35(3):121-43.

## P28: The association between physical activity and memory loss

### Fanli Yi^1^, Chih-Hsiang Yang^2,3^

#### ^1^Department of Epidemiology and Biostatistics, Arnold School of Public Health, University of South Carolina, Columbia, SC, USA; ^2^Department of Exercise Science, Arnold School of Public Health, University of South Carolina, Columbia, SC, USA; ^3^TecHealth Center to Promote Healthy Lifestyles, Arnold School of Public Health, University of South Carolina, Columbia, SC, USA

##### **Correspondence:** Fanli Yi (yif@email.sc.edu)

*BMC Proceedings 2024,* **18(8):**P28

**Objective:** The study aims to study how the association between physical activity (PA) and memory loss changed by age in White, Blacks and Hispanics using time-varying effect model (TVEM).

**Methods:** The study implemented a time-varying logistic regression model to study the association between physical activity and memory loss, using the subsets of Behavioral Risk Factor Surveillance System (BRFSS) from the 2015, 2017, and 2019 cycles for the older adults aged 45-70 years old. PA is the exposure, classified as “meeting aerobic recommendations” and “not meeting aerobic recommendations”. The outcome is memory loss, dichotomized as “Having memory loss” and “Not having memory loss”. The covariates adjusted in the model were sex, race, education, number of adults in household, marital status, BMI, current smoker, binge drinker, health insurance, fruit and vegetable consumed, overall mental health, overall physical health, history of depression, and BRFSS cohort.

**Statistical analysis:** To handle the weighted design in the national BRFSS dataset, the “%WeightedTVEM SAS macro v2.6” was used to model the population-level surveys with weightings. Each individual in the three cycles will contribute to one specific age group. The odds ratio was used to describe the association, which indicated the percentage change of memory loss corresponding to one-year increase of age. TVEM connected the time-varying coefficients using a smooth function. All the analysis was stratified by racial groups. For the covariates significantly associated with the memory loss, the association was studied in the subgroups of covariates.

**Results:** Overall, PA was associated with an improvement in memory loss from 29.09% to 44.87% in white participants 58-64 years old, 43.00%-74.24% in black participants 49-60 years old, 51.08%-99.90% and 38.70%-95.63% of Hispanic participants aged 45-49 years old and 62-70 years old respectively (Figure 1).

The association differed in the genders of White participants, with a 50% improvement in memory loss at 46-48 years old for males, and a 41.30%-62.06% improvement found in 59-64 years old females. So were the Hispanic participants, for who there appeared to be 38.70%-58.69% improvement of memory loss in males aged 62-69 years old, while a higher improvement from 48.67%-75.28% has been witnessed in women aged 60-70 years old. The association also varied in healthy BMI and overweight or obese. The white participants in healthy BMI had a higher improvement of memory loss from 58.17%-70.68% at 61-65 years old than an improvement from 41.31%-44.62% for the participants overweight or obese at 56-61 years old.

Having more than 7 days of poor physical health was associated with a greater improvement in memory loss by 56.16%-63.50% among older white individuals at 64-68 years old than 36.92%-48.25% improvement in younger individuals at 46-52 years old with less than 7 days of poor physical health. Having more than 7 days of poor mental health was associated with a 57.22%-61.43% improvement in memory loss in white participants aged 47-49 years old, and an improvement at older ages of 51-57 years old for Hispanic participants.

**Fig. 1 (Abstract P28):**
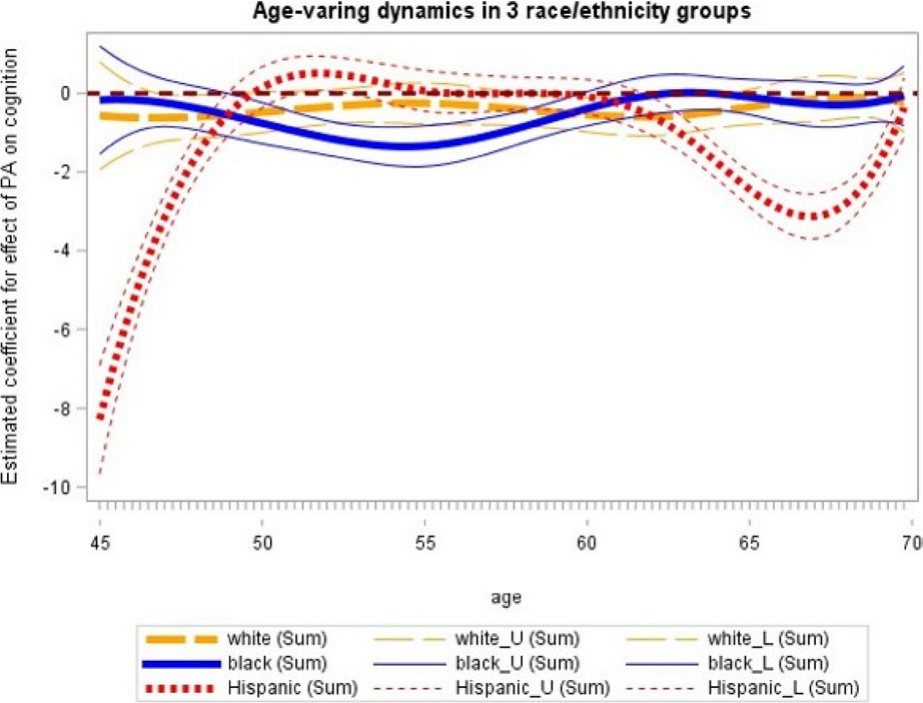
How did the memory loss change according to whether the people aged 45-70 years met aerobic recommendations or not in the White, Black, and Hispanic population

## P29: Lesion distribution in stroke

### Jiaying Yi^1^, Feresteh Kavandi Ghezeljeh^2^, Nicholas Riccardi^3^, Yuan Wang^1^, Rutvik H. Desai^2^

#### ^1^Department of Epidemiology and Biostatistics, Arnold School of Public Health, University of South Carolina, Columbia, SC, USA; ^2^Department of Psychology, College of Arts and Sciences, University of South Carolina, Columbia, SC, USA; ^3^Department of Communication Sciences and Disorders, Arnold School of Public Health, University of South Carolina, Columbia, SC, USA

##### **Correspondence:** Jiaying Yi (jiayingy@email.sc.edu)

*BMC Proceedings 2024,* **18(8):**P28

**Study Objective:** Lesion-symptom mapping (LSM) has been the gold standard approach for linking stroke lesions with behavioral deficits. LSM can lead to a better understanding of the neural bases of syndromes such as aphasia or neglect, and also provide insights into functions of specific brain regions. However, standard lesion symptom mapping analysis assumes that lesions are random and evenly distributed across the brain. However, it is known that stroke lesions are spatially related to the neurovascular structure, which can result in the co-occurrence of lesions in multiple regions. This may make it difficult to identify the independent contribution of a specific region, when it is commonly lesioned with certain other regions. Such clusters of lesions can lead to confounds in interpretation, and conflicting results between studies. This study is aimed at identifying statistically co-occurring lesions in a group of stroke survivors using a clustering approach.

**Methods:** Lesion information from magnetic resonance imaging (MRI) scans of 249 left hemisphere stroke survivors were provided by the Center for the Study of Aphasia Recovery (C-STAR) at the University of South Carolina. Lesions were traced on the anatomical scan by a neurologist, and each region from the Johns Hopkins University (JHU) atlas was assigned a score to reflect percentage of damage. We used regions that were lesioned in at least 10% of the participants and excluded the ventricles. Clusters of lesions in these JHU regions of interest (ROIs) in the left hemisphere were determined using both k-means and hierarchical clustering (Ward linkage) methods.

**Results:** By inspecting ROI clusters across multiple clustering schemes (# clusters = 5, 10, 15, 20), we observed the common patterns of co-occurring lesions. Lateral and medial temporal lobe areas (MTG, ITG, PHG, Hippocampus) were clustered together. Superior and middle temporal areas (pSTG, pMTG) were also clustered. Similarly, clusters in the frontal lobe (SFG, MFG, PrCG, PoCG), and in occipital and ventral temporal lobe (SOG, MOG, IOG, FuG) were observed across multiple clustering schemes.

**Discussion:** The clusters of lesions reflect multiple areas that are functionally relevant to multiple syndromes. For example, the language network implicated in aphasia includes frontal, temporal, and inferior parietal perisylvian regions. Our results suggest that in LSM analyses, it is difficult to distinguish contributions of temporal lobe areas, such as MTG, ITG, and PHG, since they tend to be lesioned together. Similarly, frontal areas such as IFG and MFG, PrCG also tend to be lesioned simultaneously. The controversy surrounding these regions, highly relevant to language, may partially result from this co-occurrence pattern. The spatial resolution of LSM may be limited by these clustering patterns. We suggest that LSM studies take into account these clustering when interpreting their results and report the co-occurrence structure within their data.

## P30: Unveiling inequity: state-by-state disparities in years of potential life lost by race

### Ahmeed Yinusa^1^, Misagh Faezipour^2^

#### ^1^Computational and Data Science Program, Middle Tennessee State University, Murfreesboro, TN, USA; ^2^Department of Engineering Technology, Middle Tennessee State University, Murfreesboro, TN, USA

##### **Correspondence:** Misagh Faezipour (misagh.faezipour@mtsu.edu)

*BMC Proceedings 2024,* **18(8):**P30

**Background:** Health disparities in the United States, particularly among Black, American Indian/Alaska Native (AI/AN), and Hispanic populations, pose a significant challenge [1]. These disparities manifest in premature mortality, measured by YPLL (Years of Potential Life Lost) [2]. YPLL is a summary measure of premature mortality, considering the age of death. It serves as a valuable tool for understanding and addressing health disparities by providing a more comprehensive measure of premature death’s impact compared to simply looking at death rates [3].

Methodology: The study used a descriptive, cross-sectional design to examine YPLL disparities by race and ethnicity across US states, considering social determinants of health (SDOH) as key factors. Data on YPLL rates and SDOH were obtained from the 2023 County Health Rankings [4]. The analysis involved data compilation, descriptive statistics, correlation analysis, regression analysis, and visual representation.

**Results**

Figure 1 reveals the significant disparities in premature mortality across racial and ethnic groups. Black and AI/AN individuals bear the highest YPLL rates, followed by Hispanics, while whites exhibit the lowest rates.

The graph underscores the substantial role of SDOH in YPLL disparities. Individuals facing unemployment, poverty, or limited access to healthy food and exercise opportunities experience higher YPLL rates.

Specific examples of health disparities highlighted by the graph include:

- Black people have an YPLL rate 1.2 times higher than whites, partly due to higher chronic disease rates among Black individuals.
- AI/AN people have a YPLL rate 1.3 times higher than whites, partly due to higher accident and injury rates among AI/AN individuals.
- Hispanic people have an YPLL rate 1.1 times higher than whites.

The YPLL Rates by Race in each state in Figure 2 highlights the substantial disparities in premature mortality among different racial groups in the United States. It illustrates the number of years of life lost prematurely due to mortality among individuals under 75 years of age, categorized by race and state.

The graph underscores the disproportionate impact of premature mortality on Black, AI/AN, and Hispanic populations. Black individuals exhibit the highest YPLL rates across most states, followed by AI/AN and Hispanic individuals. White individuals generally have the lowest YPLL rates.

These disparities stem from various factors, including higher rates of chronic diseases among Black individuals, higher accident and injury rates among AI/AN individuals, and lower health insurance coverage and healthcare access among Hispanic individuals.

**Conclusions:** This study revealed significant racial disparities in premature mortality across the U.S., with Black, American Indian/Alaska Native, and Hispanic individuals experiencing substantially higher rates of years of potential life lost compared to Whites. These inequities likely stem from the disproportionate impacts of social determinants of health, like poverty and limited healthcare access in minority communities. Targeted policy initiatives promoting economic opportunity, education, culturally competent healthcare, and community development in marginalized areas are needed to address the root causes driving health disparities.

**References**

1. Bauer UE, Plescia M. Addressing disparities in the health of American Indian and Alaska Native people: the importance of improved public health data. Am J Public Health. 2014;104(S3):S255–S257. Available from: 10.2105/ajph.2013.301602
2. Xu JJ, Chen JT, Belin TR, Brookmeyer RS, Suchard MA, Ramirez CM. Racial and ethnic disparities in years of potential life lost attributable to COVID-19 in the United States: an analysis of 45 states and the District of Columbia. Int J Environ Res Public Health. 2021;18(6):2921. Available from: 10.3390/ijerph18062921
3. Gardner JW, Sanborn JS. Years of potential life lost (YPLL)—what does it measure? Epidemiology. 1990;1(4):322–329. Available from: 10.1097/00001648-199007000-00012
4. County Health Rankings & Roadmaps. 2023 County health rankings national findings report. County Health Rankings & Roadmaps. Available from: https://www.countyhealthrankings.org/reports/2023-county-health-rankings-national-findings-report

**Fig. 1 (Abstract P30):**
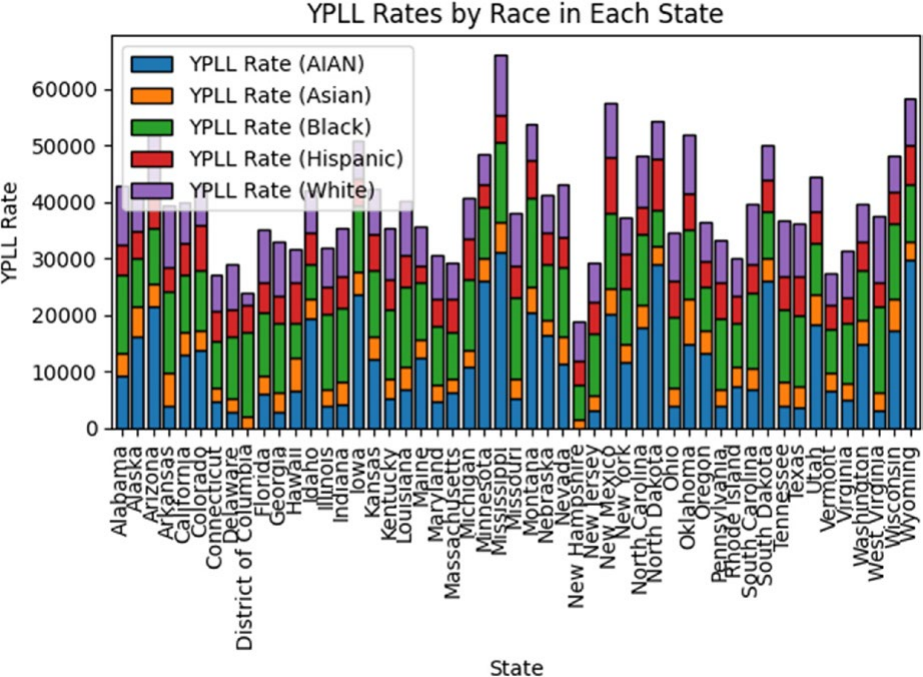
YPLL and SDOH in the United States

**Fig. 2 (Abstract P30):**
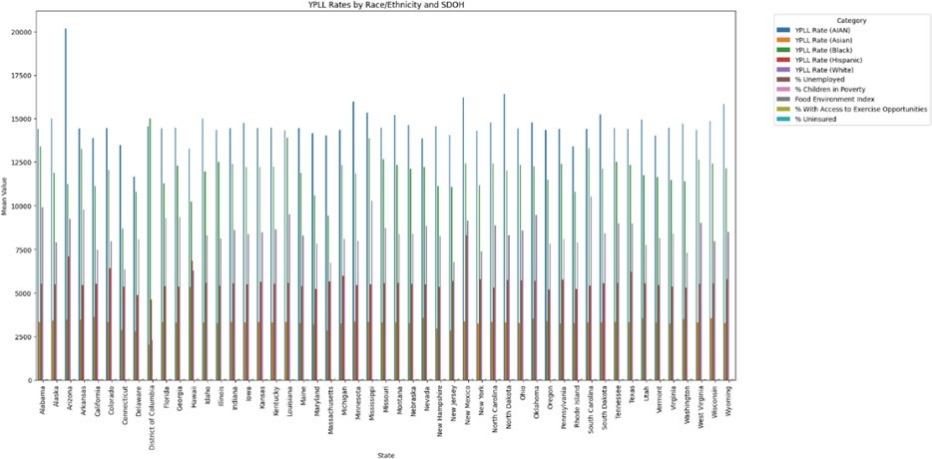
Disparities in premature deaths by race and state

### Supplementary Information

**Additional file 1:** Appendix 1

