# Supplementary material for: Meeting Abstracts from the 5th National Big Data Health Science Conference
Source: BMC Proc. 2024 May 16;18(Suppl 8):9. doi: 10.1186/s12919-024-00292-3 (PMC11097451; doi:10.1186/s12919-024-00292-3)
Supplement: Supplementary file 1 — Additional file 1: Appendix 1 [file 12919_2024_292_MOESM1_ESM.docx]

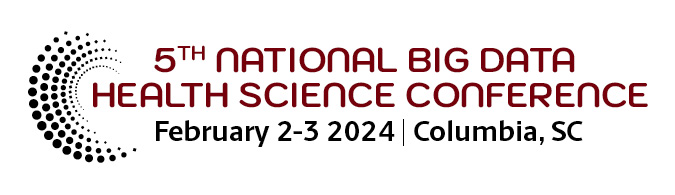


Thursday, February 1

Exploring Data Horizons: Using SAS Viya for Dynamic Exploratory Modeling

Optional Pre-Conference Workshop | University of South Carolina Campus

Pre-registration required

1:00pm – 5:00pm

Linda Jordan, SAS

Friday, February 2

Registration & Breakfast | Ballroom B

8:00am – 9:00am

Plenary Sessions | Ballroom A

Opening & Welcoming Remarks

9:00am – 9:15am

Donna Arnett, Provost, University of South Carolina

Announcement of Case Competition Awards

9:15am – 9:30am

G. Thomas Chandler, Dean, Arnold School of Public Health, University of South Carolina

Enhancing Institutional Data Science Capacity

9:30am – 10:15am

Raphael D. Isokpehi, Office of Data Science Strategy, National Institutes of Health

Real-World Big Data and Analytics

10:15am – 11:00am

John Hsu, Harvard Medical School & Massachusetts General Hospital

Coffee Break | Pre-function

11:00am – 11:15am

Boosting Data Analytics Through High-Fidelity
Synthetic Data

11:15am – 12:00pm

Xiaotong Shen, University of Minnesota

Exploring Bias in AI & Machine Learning

12:00pm – 12:45pm

Jim Box, SAS

Underrepresented Minorities in Data Science Applied to Health Career Development Luncheon
Congaree Room | Pre-registration required

12:00-1:30

Raphael D. Isokpehi, Office of Data Science Strategy, National Institutes of Health

Kay Thomas, Assistant Dean for Student Affairs, South Carolina Honors College, University of South Carolina

Julian R. Williams, Vice President, Office of Access and Opportunity, University of South Carolina

Lunch | Ballroom B

12:45pm – 1:30pm

Breakout Sessions (S1-S3)

1:30pm – 3:30pm

S1. Electronic Health Records Core Breakout Session 1 Ballroom A

Moderator: Jiajia Zhang (University of South Carolina)

Assessing the Properties of Patient-Specific Treatment Effect Estimates from Causal Machine Learning Algorithms Under Essential Heterogeneity

John Brooks (University of South Carolina)

Black Maternal Care: A Call to Action for Engineering, Data Science, Electronic Health Records and Justice

Fay Cobb Payton (North Carolina State University)

Harmonization of Multiple HIV Related Data Sources in Sub Saharan Africa: Lessons Learned from the Boloka Project

Refilwe Nancy Phaswana-Mafuya (SAMRC/UJ Pan African Centre for Epidemics Research Extramural Unit)

Disparities in Prenatal Telehealth Uptake Among Birthing Individuals in The United States, June 2018-May 2022: Data from The National COVID-19 Cohort Collaborative (N3C)

Peiyin Hung (University of South Carolina)

Barriers to Unlocking Big Data to Explore LGBTQ+ Populations in South Carolina

Jennifer May (University of South Carolina)

S2. Genomic Core Breakout Session | Senate Room

Moderators: Homayoun Valafar and Hui Chen (University of South Carolina)

The Heterogeneity of Macrophages in SEB-Induced Acute Respiratory Distress Syndrome

Kiesha Wilson (University of South Carolina)

An Algorithm for the Constrained Longest Common Subsequence and Substring Problem

Rao Li (University of South Carolina, Aiken)

miRGD: miRNA-Gene-Disease Association Prediction Based on GCN

Yuhang Chen (Yale University)

A Robust Rarefaction Method for Evaluating Alpha Diversities in TCR Sequencing Data

Mo Li (University of Louisiana at Lafayette)

Wastewater-Based Microbiomics Unveils Novel Insights into Population-Level Human Gut Microbial Interactions to Support Precision Public Health Strategies

Devin Bowes (University of South Carolina)

Integration of Molecular and Clinical Data from Cancer Patients

Anna Blenda (University of South Carolina, Greenville)

S3. Geospatial Core Breakout Session
Hall of Fame Meeting Room

Moderator: Melissa Nolan (University of South Carolina)

Disparities in Pedestrian and Bicycle Crashes by Social Vulnerability Across South Carolina

Andrew Kaczynski (University of South Carolina)

The Impact of Wildfires on Mental Health

Tamara Sheldon (University of South Carolina)

Integrating GIS into Criminal Justice Research

Hunter Boehme & Cory Schnell (University of South Carolina)

A Two-Pronged Big Data Approach to Critically Analyze Strongyloides Stercoralis Infections Among Rural, Impoverished South Carolina Residents

Matthew Haldeman (University of South Carolina)

Classifying Urban and Rural Gentrified Neighborhoods in the United States: A Repeated Measures Latent Profile Analysis

Parthenia Luke (University of South Carolina)

Identifying Geographic Disparities in The Relationship Between Neighborhood Walkability and Active Transportation Crashes Within South Carolina

Anna Chupak (University of South Carolina)

Coffee Break | Pre-function

3:00pm – 3:45pm

Breakout Sessions (S4-S6)

3:45pm – 5:45pm

S4. AI for Sensing & Diagnosis Core Breakout Session
Senate Room

Moderator: Christopher Sutton (University of South Carolina)

Benchmarking Zero-Shot Inference of Advanced Oncology Concepts and Relations with Large Language Models and Expert-Curated Dataset

Madhumita Sushil (University of California, San Francisco )

Toward Defining a Taxonomy for Ostomy Nursing Care Using Natural Language Processing

LaToya McDonald (Clemson University)

Leveraging Microbiome Big Data to Connect Gut Microbial Metabolites to Inflammatory Bowel Disease

Jie Li (University of South Carolina)

Improving Healthcare Delivery with Artificial Intelligence: A Diagnostic and Prescription Recommender System

Damilare Ogungbesan (Middle Tennessee State University)

S5. Electronic Health Records Core Breakout Session 2
Ballroom A

Moderator: Xueying Yang (University of South Carolina)

Postpartum Telehealth Uptake among Birthing Individuals in the United States, June 2018-May 2022: Data from the National COVID-19 Cohort Collaborative

Jiani Yu (Cornell University)

South Carolina Breast and Cervical Cancer Data in Action

Beth Williams (South Carolina Department of Health & Environmental Control)

Optimizing the Monitoring of RMNCAH+N Program Implementation in Northern Nigeria using a Data Visualization Dashboard: The Kaduna State Experience

Collins Imarhiagbe (Centre for Integrated Health Programs, Abuja, Nigeria)

The Impacts of “Baby-Friendly” Hospital Designations

Lindsey Woodworth (University of South Carolina)

Big Data and Nursing Wisdom: The Key to Unlock Healthcare Innovation

Ramya Govindarajan (Emory University)

Deconstructing Multi-Institutional EHR Data: Experiences from a Pediatric Diabetes Surveillance Effort in South Carolina

Angela Liese (University of South Carolina)

S6. Social Media Core Breakout Session
Hall of Fame Meeting Room

Moderators: Shan Qiao (University of South Carolina) & Zhenlong Li (Pennsylvania State University)

What do Foster Parent Associations Communicate on Facebook? Analyses Using Unsupervised Machine-Learning Method

Anli Xiao (University of South Carolina)

Identifying Perinatal Opioid-Related Tweet Themes on Twitter

Dezhi Wu (University of South Carolina)

Association Between Immigrant Concentration and Mental Health Service Utilization in the United States Over Time: A Geospatial Big Data Analysis

Zhenlong Li (Pennsylvania State University)

Utilizing Pre-Trained Language Models for Identifying Vaping-Related Discussions on Reddit During the EVALI Outbreak

Yang Ren (University of South Carolina)

Estimating Hourly Neighborhood Population using Mobile Phone Data

Huan Ning (Pennsylvania State University)

Qualitative Coding of Interview using Generative AI

Yuhao Kang (University of South Carolina)

Networking Reception
Columbia Metropolitan Convention Center

6:00pm – 8:00pm

Saturday, February 3

Registration & Breakfast | Ballroom B

8:30am – 9:30am

Plenary Sessions | Ballroom A

Empowering Public Health Response: A Path Towards Harnessing the Power of Big Data through KHIE

9:30am – 10:15am

Andrew Bledsoe, Commonwealth of Kentucky Cabinet for Health & Family Services

Novel Applications of Large Language Models in Healthcare

10:15am – 11:00am

Ioannis Paschalidis, Boston University

Coffee Break | Pre-function

11:00am – 11:15am

Mining Large Data to Guide Policy and

Improve Equity of Care

11:15am – 12:00pm

Marcela Horvitz-Lennon, RAND Corporation

Lunch | Ballroom B

12:00pm – 1:00pm

Poster Sessions & Dessert | Ballroom C

1:00pm – 3:00pm

Poster Session 1

1:00pm – 1:50pm

P1. A System Dynamics View of Patient’s Perception of AI and Big Data Adoption in Healthcare

Ashiat Adeogun (Middle Tennessee State University)

P2. History of Pregnancy Loss and Depression Among Women of Reproductive Age: Evidence from NHANES 2007-2018

Syeda Shehirbano Akhtar (University of South Carolina)

P3. Rural-Urban Differences in The Prevalence of Low Back Pain and Pelvic Pain During Pregnancy

Songyuan Deng (University of South Carolina)

P4. Improving Cost-Effectiveness for Data Migration in Healthcare Environments Using Serverless Architecture

Prashant Duhoon (University of South Carolina)

P5. Hypersensitivity Associations with Antibiotics: A Pharmacovigilance Study of The FDA Adverse Event Reporting System (FAERS)

Lucy Edwards (University of South Carolina)

P6. Utility Of South Carolina’s Statewide Telephone Helpline In COVID-19 Prevention and Control

Emmanuel Julceus (University of South Carolina)

P7. Understanding the Influence of Health Insurance and Medical Home Environments on Healthcare Utilization for Children with Autism Spectrum Disorder: A Logistic Regression Analysis of NSCH 2016-2021

Gahssan Mehmood (University of South Carolina)

P8. Rural-Urban and Racial Differences in Cesarean Deliveries Before and During The COVID-19 Pandemic In South Carolina

Cassie Odahowski (University of South Carolina)

P9. Effect Of COVID-19 Pandemic on Medical and Preventive Healthcare Utilization Among US Children Aged 0 To 17 Years: Evidence from NSCH 2016-2021

Ibitein Okeafor (University of South Carolina)

P10. Leveraging Big Heterogenous HIV-Related Data in The Era of Protection of Personal Data Act in Sub Saharan Africa: Lessons Learned from The Boloka Project

Edith Phalane (SAMRC/UJ Pan African Centre for Epidemics Research Extramural Unit)

P11. Scoring of Pediatric Respiratory Syncytial Virus (RSV) Infection Severity: A Systematic Review

Zoe Sanders (University of South Carolina)

P12. Association of Mental Health Service Utilization and Mental Health Diagnosis with Placement Instability among Foster Children in a Southeastern State

Nelis Soto-Ramirez (University of South Carolina)

P13. Neighborhood Disinvestment and Racial/Ethnic Disparities in Peripartum Cardiomyopathy in California, from 2004-2019

Curisa Tucker (University of South Carolina)

P14. The Association Between Physical Activity and Memory Loss

Fanli Yi (University of South Carolina)

P15. Lesion Distribution in Stroke

Jiaying Yi (University of South Carolina)

P16. Unveiling Inequity: State-By-State Disparities in Years of Potential Life Lost by Race

Ahmeed Yinusa (Middle Tennessee State University)

P17. Cholinesterase Inhibitor-Anticholinergic Prescribing Cascades and Incidence of Delirium and Falls in Patients with Alzheimer’s Disease and Related Dementias

Ismaeel Yunusa (University of South Carolina)

Poster Session 2

2:00pm – 2:50pm

P1. Divergence and Intersection of Practical and Implementable Compliance with HIPAA’s Privacy Rule and The FTC’s Health Breach Notification Rule: A Case Study

Marilyn Gartley (University of South Carolina)

P2. Visualizing Major Healthcare Breaches

Sharon Gumina (University of South Carolina)

P3. The Benefits of Electronic Case Reports for Reportable Disease Tracking by Public Health Agencies

Margaret Iiams (South Carolina Department of Health & Environmental Control)

P4. Building A Dashboard: Visualizing South Carolina Cancer Program Efforts

Jaron King (South Carolina Department of Health & Environmental Control)

P5. An Exploration of Lag in Payer Ascertainment Among Maternal Delivery Claims In South Carolina: Potential Implications for Timely Maternal Care

Linga Murthy Kotagiri (University of South Carolina)

P6. Uncertainty Measurement in Medical Image Interpretation

Shuge Lei (University of South Carolina)

P7. Using ChatGPT in Literature Review: A Validation Pilot Study in The Areas of HIV Medicine Adherence

Naser Mohammad Lessani (Pennsylvania State University)

P8. Exploring Disparities in Exposure to Fine Particulate Matter (PM2.5) According to Social Vulnerability In South Carolina From 2000-2018

Erin Looney (University of South Carolina)

P9. Exploring the Impact of ADHD Medication on School Absenteeism: A Causal Analysis Of South Carolina Medicaid Data

Zichun Meng (University of South Carolina)

P10. Feasibility of Applying Big Data Approaches on Diverse Institutional Research Health-Related Datasets in an Open Distance Higher Education Institution in South Africa

Motlatso Mlambo (University of South Africa)

P11. Prevention Quality Indicators and Preventable Hospital Use Among Adults with Traumatic Spinal Cord Injury

David Murday (University of South Carolina)

P12. Cyclical Learning Rates (CLR’S) For Improving Training Accuracies and Lowering Computational Cost

Shrikant Pawar (Claflin University)

P13. Identifying Genetic Contributions to Adverse Drug Reactions Using Big Data

Scott Reed (University of Colorado Denver)

P14. Augmented Intelligence (AI) At the Point-Of-Care: The FQHC As the Ideal Site for AI In Primary Care

Dean Slade (Cooperative Health Centers, Inc.)

P15. Health Big Data Pipeline Optimization: Efficiency of Containerized Applications in Experimental Data Engineering with Distributed Computing

Ehsan Soltanmohammadi (University of South Carolina)

P16. Leveraging Big Data in Affective Neuroscience

Xuan Yang (University of South Carolina)

Additional e-Posters found in the app

Bayesian Semiparametric Geoadditive Modelling of Underweight Malnutrition of Children Under-5 Years in Ethiopia

Endeshaw Assefa Derso (University of Messina, Italy)

Association of Joint Exposure to Air Pollutants, Genetic Risk, and Incident Thyroid Nodules: A Prospective Cohort Study

Yu Bao (Guangxi Medical University, Nanning, Guangxi, China)

Association Between Work Stress and Mental Health in Chinese Public Health Workers: Mediating Role of Social Support and Self-Efficacy

Yinqiao Dong (Shanghai Jiao Tong University School of Medicine, Shanghai, China)

A Data Science Approach to Precision Medicine: Allostatic Load as a Predictor of Cardiovascular Disease

Otto Ikome (Jackson State University, Jackson, Mississippi)

Moderate-to-Vigorous Physical Activity and Depressive Symptoms using Compositional Data: The Moderating Role of Socioeconomic Status and Race

Yujie Liu, (Shanghai Jiao Tong University School of Medicine, Shanghai, China)

Physical Activity, Sedentary Behavior and BMI Among Adolescents: The Mediate Role of Weight Concern Regulated by Body Image Perception

Shuoyuan Tan (Shanghai Jiao Tong University School of Medicine, Shanghai, China)

NIH Trainee Session | Ballroom A

1:00pm – 3:00pm

R25 Big Data Health Science Fellow Junior Faculty

**Tad Dallas**, Department of Biological Sciences, University of South Carolina

**Caroline Derrick**, School of Medicine-Columbia, University of South Carolina

**Jie Li**, Department of Chemistry and Biochemistry, University of South Carolina

**Cheuk Chi Tam**, Department of Health Promotion, Education, and Behavior

R25 Big Data Health Science Community Scholars

**Adelero Adebajo**, Cooperative Health

**Ashley Green**, South Carolina Department of Health & Environmental Control

**Carmen Julious**, PALSS, Inc.

**Andrew Lynch**, Hope Health

Plenary Sessions | Ballroom A

The Impact of Deep Learning for Creating Novel Effectors of Biological Functions

3:00pm – 3:45pm

Gaetano T. Montelione, Rensselaer Polytechnic Institute

Data Science Research & Training Opportunities across the NIH: The NIH Funders Panel

3:45pm – 4:30pm

Moderator: Lori Scott-Sheldon, Division of AIDS Research, National Institute of Mental Health, National Institutes of Health

This special panel features six program officers/directors from NIH institutes including the National Institute of Mental Health; the National Heart, Lung, and Blood Institute; the National Institute of Allergy and Infectious Diseases; the Office of Data Science Strategy, and the All of Us Program. You are welcome to submit your questions ahead of time on the Whova app.

**Reed Shabman**, Office of Data Science and Emerging Technologies, National Institute of Allergy and Infectious Disease, National Institutes of Health

**Raphael D. Isokpehi**, Training, Workforce Initiatives, and Community Engagement (TWICE), Office of Data Science Strategy, National Institutes of Health

**Rosemary McKaig**, Division of AIDS, National Institute of Allergy and Infectious Diseases, National Institute of Health

**Sidd Shenoy**, National Heart, Lung, and Blood Institute, National Institutes of Health

**Romuladus Azuine**, All of Us Research Program, National Institutes of Health

Closing Remarks & Announcement of Awards

4:30pm – 4:45pm
